# Supplementary material for: Enhancing phosphate-solubilising microbial communities through artificial selection
Source: Nat Commun. 2024 Feb 23;15:1649. doi: 10.1038/s41467-024-46060-x (PMC10884399; doi:10.1038/s41467-024-46060-x)
Supplement: Supplementary file 1 — Supplementary Information [file 41467_2024_46060_MOESM1_ESM.pdf]

Supplementary Tables and Figure

Supplementary Table 1: Results of Analysis of Variance (ANOVA) and Generalised Linear Model (GLM) comparing the phosphate solubilised by the fractions of the source soil microbial community

**ANOVA**		**GLM**	
**Effect**	**df; F value; p value**	**Coefficient**	**Estimate ± SE; *t* value, p value**
Fraction	2,6; 0.217; 0.811	Intercept	390.156 ± 12.044; 32.394; <0.001
		HE	-7.058 ± 17.033; -0.414; 0.693
		NE	-11.092 ± 17.033; -0.651; 0.539

df: degrees of freedom, SE: standard error. Fractioned communities: EE: easy-to-extract, HE: hard-to-extract, NE: not-extractable. All statistical tests were two-sided.

Supplementary Table 2: Results of ANOVA and GLM comparing the phosphate solubilised by the dilutions of the source soil microbial community

**ANOVA**		**GLM**	
**Effect**	**df; F value; p value**	**Coefficient**	**Estimate ± SE; *t* value; p value**
Dilution	2,6; 1.799; 0.244	Intercept	394.179 ± 6.986; 56.425; <0.001
		D1	-15.125 ± 9.880; -1.531; 0.177
		D2	-17.142 ± 9.880; -1.735; 0.133

df: degrees of freedom, SE: standard error. Diluted communities: D0: undiluted, D1: diluted 1:2, D2: diluted 1:4. All statistical tests were two-sided.

Supplementary Table 3: Results of ANOVA and GLM comparing the phosphate solubilised by the dilutions and fractions of the parental phase

ANOVA		GLM	
**Effect**	**df; F value; p value**	**Coefficient**	**Estimate ± SE; *t* value; p value**
Dilution Fraction	5, 184; 1.478; 0.199	Intercept	392.364 ± 3.086; 127.155; <0.001
		HE	4.010 ± 4.234; 0.947; 0.345
		NE	4.808 ± 4.329; 1.111; 0.268
		D0	-5.338 ± 4.300; -1.243; 0.216
		D1	-0.3643 ± 4.329; -0.084; 0.933
		D2	0.712 ± 4.300; 0.166; 0.868

df: degrees of freedom, SE: standard error. Fractioned communities: EE: easy-to-extract, HE: hard-to-extract, NE: not-extractable. Diluted communities: D0: undiluted, D1: diluted 1:2, D2: diluted 1:4. All statistical tests were two-sided.

Supplementary Table 4: Results of ANOVA, GLM and Linear mixed-effect model (LMM) comparing the phosphate solubilised by the communities of the active and random selection line of the environmental perturbation phase

ANOVA		GLM		LMM	
**Effect**	**df; F value; p value**	**Coefficient**	**Estimate ± SE; *t* value; p value**	**Coefficient**	**Estimate ± SE; *t* value**
Selection type	1, 201; 0.057; 0.812	Intercept	0.678 ± 0.057; 11.870; <0.001	Intercept	0.706 ± 0.064; 10.897
		Random selection	-0.024 ± 0.099; -0.238; 0.812	Random selection	0.126 ± 0.113; 1.113

df: degrees of freedom, SE: standard error. All statistical tests were two-sided.

Supplementary Table 5: Results of ANOVA and GLM comparing the phosphate solubilised by the communities of the parental phase and environmental perturbation phase

**ANOVA**		**GLM**		**LMM**	
**Effect**	**df; F value; p value**	**Coefficient**	**Estimate ± SE; *t* value; p value**	**Coefficient**	**Estimate ± SE; *t* value**
Phase	1, 207; 8.093; 0.005	Intercept	1.345 ± 0.300; 4.547; < 0.001	Intercept	1.347 ± 0.300; 5.881
Selection type	1, 207; 0.054; 0.817	OE	-0.669 ± 00.301; -2.219; 0.028	OE	-1.249 ± 0.240; -5.206
Phase × Selection type	1, 207; 0.003; 0.953	R	0.005 ± 0.483; 0.011; 0.9910	R	0.004 ± 0.374; 0.010
		R × OE	-2.029 ± 0.493; -0.059; 0.953	R × OE	0.326 ± 0389; 0.837

df: degrees of freedom, SE: standard error. R: Random selection, OE: Environmentally perturbed phase. All statistical tests were two-sided.

Supplementary Table 6: Results of the post hoc tests following ANOVA and GLM and LMER comparing the phosphate solubilised by the communities of the environmentally perturbed offspring phase to the parental phase

		**ANOVA + Tukey**	**GLM + Estimated marginal means (EMMs)**	**LMM + EMMs**
**Effect**	**Contrast**	***t* ratio; p value**	***t* ratio; p value**	***t* ratio; p value**
Phase	PP – OE	2.219; 0.028	2.770; 0.006	3.090; 0.002
Selection type	R – B	0.011; 0.991	0.037; 0.971	0.254; 0.7998
Interaction	PB – OBE	-; 0.364	2.219; 0.083	2.741; 0.041
	PB – PR	-; 0.999	-0.011; 0.9910	-0.010; 0.992
	PB – ORE	-; 0.364	2.257; 0.0827	2.222; 0.067
	OBE – PR	-; 0.454	-1.745; 0.124	-2.145; 0.67
	OBE – ORE	-; 0.999	0.239; 0.973	-0.98.; 0.421
	PR – ORE	-; 0.454	1.787; 0.124	1.794; 0.112

PP: Parental phase, OE: Environmental perturbed phase. B: Active selection line, R: Random selection line. PB: Parental actively selected communities, PR: Parental randomly selected communities. OBE: environmentally perturbed actively selected communities, ORE: environmentally perturbed randomly selected communities. All statistical tests were two-sided and p values were corrected for multiple comparison using the Benjamini-Hochberg procedure.

Supplementary Table 7: Results of ANOVA and GLM comparing the phosphate solubilised by the communities of parental phase and propagation phase

ANOVA		GLM		LMM	
**Effect**	**df; F value; p value**	**Coefficient**	**Estimate ± SE; *t* value; p value**	**Coefficient**	**Estimate ± SE; *t* value**
Phase	1,26; 0.007; 0.933	Intercept	2.022 ± 0.432; 4.680; <0.001	Intercept	2.022 ± 0.608; 3.325
Selection type	1,26; 14.835; 0.001	OP	0.468 ± 0.516; 0.906; 0.373	OP	1.324 ± 1.122; 1.180
Phase × Selection type	1,26; 2.100; 0.159	R	-0.6713 ± 0.748; -0.897; 0.378	R	-0.671 ± 0.883; -0.760
		OP × R	-1.296 ± 0.8944; -1.449; 0.159	OP × R	-2.2617 ± 1.766; -1.281

df: degrees of freedom, SE: standard error. R: Random selection, OP: Propagation phase. All statistical tests were two-sided.

Supplementary Table 8: Results of the post hoc tests following ANOVA and GLM comparing the phosphate solubilised by the communities of parental phase and propagation phase

**Effect**	**Contrast**	**ANOVA + Tukey *t* ratio; p value**	**GLM + EMMs t ratio; p value**	**LMM + EMMs *t* ratio; p value**
Phase	PP – OP	0.906; 0.373	0.404; 0.690	0.284; 0.779
Selection type	R – B	-0.897; 0.378	-2.950; 0.001	-2.501; 0.025
Interaction	PB – OBP	-; 0.806	-0.906; 0.378	-0.663; 0.513
	PB – PR	-; 0.806	0.897; 0.378	0.760; 0.513
	PB – ORP	-; 0.227	2.547; 0.051	1.923; 0.202
	OBP – PR	-; 0.696	1.692; 0.205	1.309; 0.410
	OBP – ORP	-; 0.014	4.016; 0.003	3.404; 0.026
	PR – ORP	-; 0.806	1.135; 0.378	0.889; 0.513

PP: Parental phase, OP: Propagation phase. B: Active selection line, R: Random selection line. PB: Parental actively selected communities, PR: Parental randomly selected communities. OBP: propagated, actively selected communities, ORP: propagated, randomly selected communities. All statistical tests were two-sided and p values were corrected for multiple comparison using the Benjamini-Hochberg procedure.

Supplementary Table 9: Results of ANOVA and GLM comparing the phosphate solubilization between the artificial selection methods

ANOVA		GLM		LMM	
**Effect**	**df; F value; p value**	**Coefficient**	**Estimate ± SE; *t* value; p value**	**Coefficient**	**Estimate ± SE; *t* value**
Method	1,221; 12.498; <0.001	Intercept	1.635 ± 0.019; 87.569; <0.001	Intercept	1.834 ± 0.030; 61.851
Selection type	1,221; 1.702; 0.193	OP	0.378 ± 0.072; 5.246; <0.001	OP	0.184 ± 0.180; 1.027
Method × Selection type	1,221; 15.140; <0.001	R	-0.004 ± 0.032; -0.136; 0.892	R	-0.061 ± 0.075; -0.802
		OP × R	-0.441 ± 0.113; -3.891; <0.001	OP × R	-0.404 ± 0.262; -1.544

df: degrees of freedom, SE: standard error. R: Random selection, OP: Propagated communities. All statistical tests were two-sided.

Supplementary Table 10: Results of the post hoc tests following ANOVA and GLM comparing the phosphate solubilised between the artificial selection methods

**Effect**	**Contrast**	**ANOVA + Tukey** *t* **ratio; p value**	**GLM + EMMs** *t* **ratio; p value**	**LMM + EMMs** *t* **ratio; p value**
Method	OP – OE	5.246; <0.001	2.787; 0.006	2.821; 0.008
Selection type	R – B	-0.136; 0.892	-3.969; <0.001	-4.340; <0.001
Interaction	OBP – OBE	-; < 0.001	5.246; <0.001	6.063; <0.001
	ORE – OBE	-; 0.999	-0.136; 0.892	0.838; 0.423
	ORP – OBE	-; 0.999	-0.785; 0.570	-1.054; 0.361
	ORE – OBP	-; < 0.001	-5.135; <0.001	-5.162; <0.001
	ORP - OBP	-; < 0.001	-4.102; <0.001	-4.986; <0.001
	ORP – ORE	-; 0.999	-0.716; 0.570	-1.386; 0.265

OE: Environmental perturbed phase, OP: propagation phase. B: Active selection line, R: Random selection line. OBE: environmentally perturbed actively selected communities, ORE: environmentally perturbed randomly selected communities. OBP: propagated, actively selected communities, ORP: propagated, randomly selected communities. All statistical tests were two-sided and p values were corrected for multiple comparison using the Benjamini-Hochberg procedure.

Supplementary Table 11: Results of ANOVA and GLM comparing the total plant fresh and dry biomass per inoculated community (treatment)

	ANOVA		GLM	
**Biomass**	**Effect**	**df; F value; p value**	**Coefficient**	**Estimate ± SE; *t* value, p value**
Fresh	Treatment	7, 125; 1.058; 0.395	Intercept	0.199 ± 0.005; 41.484; <0.001
			C2	-0.002 ± 0.005; -0.383; 0.702
			OBE	-0.004 ± 0.005; -0.696; 0.488
			OBP	0.003 ± 0.005; 0.521; 0.603
			ORE	-0.008 ± 0.005; -1.374; 0.172
			ORP	-0.001 ± 0.005; -0.178; 0.859
			PB	-0.007 ± 0.005; -1.323; 0.188
			PR	-0.006 ± 0.05; -1.109; 0.269
Dry	Treatment	7, 125; 1.322; 0.245	Intercept	3.191 ± 0.014; 222.255; 0.001
			C2	0.018 ± 0.016; 1.137; 0.258
			OBE	-0.017 ± 0.016; -1.026; 0.307
			OBP	-0.014 ± 0.016; -0.890; 0.3753
			ORE	0.004 ± 0.016; 0.250; 0.803
			ORP	-0.023 ± 0.016; -1.454; 0.148
			PB	-0.003 ± 0.016; -0.753; 0.4527
			PR	-0.012 ± 0.016; -0.753; 0.453

df: degrees of freedom, SE: standard error. C1: uninoculated nutrient solution containing insoluble iron phosphate. C2: uninoculated, sterile control with nutrient solution containing soluble KH_2PO_4. PB: parental best-performing community, PR: parental randomly selected community. OBE: environmentally perturbed best-performing community, ORE: environmentally perturbed randomly selected community. OBP: Propagated best-performing community, ORP: propagated randomly selected community. All statistical tests were two-sided.

Supplementary Table 12: Results of ANOVA and GLM comparing the shoots plant fresh and dry biomass per inoculated community (treatment)

	ANOVA		GLM	
**Biomass**	**Effect**	**df; F value; p value**	**Coefficient**	**Estimate ± SE; *t* value, p value**
Fresh	Treatment	7, 125; 1.270; 0.271	Intercept	1.148 ± 0.036; 32.230; <0.001
			C2	0.017 ± 0.038; 0.432; 0.667
			OBE	0.055 ± 0.038; 1.443; 0.152
			OBP	-0.015 ± 0.039; -0.388; 0.699
			ORE	0.075 ± 0.039; 1.932; 0.056
			ORP	0.022 ± 0.039; 0.566; 0.573
			PB	0.058 ± 0.038; 1.508; 0.134
			PR	0.043 ± 0.039; 1.113; 0.268
Dry	Treatment	7, 125; 1.213; 0.300	Intercept	0.609 ± 0.004; 136.651; <0.001
			C2	-0.004 ± 0.005; -0.912; 0.363
			OBE	0.003 ± 0.005; 0.529; 0.598
			OBP	0.003 ± 0.005; 0.560; 0.576
			ORE	-0.004 ± 0.005; -0.918; 0.361
			ORP	0.006 ± 0.005; 1.224; 0.223
			PB	-0.001 ± 0.005; -0.262; 0.794
			PR	0.002 ± 0.005; 0.515; 0.608

df: degrees of freedom, SE: standard error. C1: uninoculated nutrient solution containing insoluble iron phosphate. C2: uninoculated, sterile control with nutrient solution containing soluble KH_2PO_4. PB: parental best-performing community, PR: parental randomly selected community. OBE: environmentally perturbed best-performing community, ORE: environmentally perturbed randomly selected community. OBP: Propagated best-performing community, ORP: propagated randomly selected community. All statistical tests were two-sided.

Supplementary Table 13: Results of ANOVA and GLM comparing the roots plant fresh and dry biomass per inoculated community (treatment)

	ANOVA		GLM	
**Biomass**	**Effect**	**df; F value; p value**	**Coefficient**	**Estimate ± SE; *t* value, p value**
Fresh	Treatment	7, 122; 1.257; 0.277	Intercept	0.547 ± 0.006; 93.117; <0.001
			C2	-0.004 ± 0.007; -0.570; 0.570
			OBE	0.008 ± 0.007; 1.111; 0.269
			OBP	-0.001 ± 0.007; -0.177; 0.860
			ORE	-0.006 ± 0.007; -0.948; 0.345
			ORP	0.008 ± 0.007; 1.171; 0.244
			PB	-0.003 ± 0.007; -0.477; 0.634
			PR	-0.005 ± 0.007; -0.694; 0.489
Dry	Treatment	7, 122; 0.892; 0.515	Intercept	6.500e-01 ± 2.962e-03; 219.424; <0.001
			C2	-4.613e-03 ± 3.326e-03; -1.387; 0.168
			OBE	1.574e-03 ± 3.447e-03; 0.457; 0.649
			OBP	1.728e-04 ± 3.276e-03; 0.053; 0.958
			ORE	-8.233e-05 ± 3.326e-03; -0.025; 0.980
			ORP	7.394e-04 ± 3.276e-03; 0.226; 0.822
			PB	3.398e-03 ± 3.326e-03; 1.022; 0.309
			PR	8.897e-04 ± 3.440e-03; 0.259; 0.796

df: degrees of freedom, SE: standard error. C1: uninoculated nutrient solution containing insoluble iron phosphate. C2: uninoculated, sterile control with nutrient solution containing soluble KH_2PO_4. PB: parental best-performing community, PR: parental randomly selected community. OBE: environmentally perturbed best-performing community, ORE: environmentally perturbed randomly selected community. OBP: Propagated best-performing community, ORP: propagated randomly selected community. All statistical tests were two-sided.

Supplementary Table 14: Results of ANOVA and GLM comparing the soluble phosphate in the nutrient solution per inoculated community (treatment) per timepoint

	ANOVA		GLM	
**Days after inoculation**	**Effect**	**df; F value; p value**	**Coefficient**	**Estimate ± SE; *t* value, p value**
4 days	Treatment	6,30; 3.040; 0.0192	Intercept	3.145 ± 0.200; 15.686; <0.001
			PB	-0.272 ± 0.217; -1.257; 0.218
			PR	-0.282 ± 0.217; -1.304; 0.202
			OBE	-0.222 ± 0.217; -1.024; 0.314
			ORE	-0.171 ± 0.217; -0.792; 0.435
			OBP	0.908 ± 0.217; 4.191; <0.001
			ORP	0.928 ± 0.217; 4.284; <0.001
7 days	Treatment	6,30; 5.521; 0.001	Intercept	1.066 ± 0.151; 7.080; <0.001
			PB	-0.033 ± 0.163; -0.206; 0.839
			PR	-0.185 ± 0.163; -1.135; 0.265
			OBE	-0.256 ± 0.163; -1.573; 0.126
			ORE	-0.133 ± 0.163; -0.817; 0.421
			OBP	0.366 ± 0.163; 2.247; 0.032
			ORP	0.438 ± 0.163; 2.693; 0.012
21 days	Treatment	6,30; 3.140; 0.017	Intercept	0.951 ± 0.112; 8.484; <0.001
			PB	-0.146 ± 0.121; -1.209; 0.236
			PR	0.038 ± 0.121; 0.311; 0.758
			OBE	-0.091 ± 0.121; -0.753; 0.457
			ORE	-0.133 ± 0.121; -1.094; 0.283
			OBP	-0.416 ± 0.121; -3.432; 0.002
			ORP	-0.232 ± 0.121; -1.919; 0.065

df: degrees of freedom, SE: standard error. PB: parental best-performing community, PR: parental randomly selected community. OBE: environmentally perturbed best-performing community, ORE: environmentally perturbed randomly selected community. OBP: Propagated best-performing community, ORP: propagated randomly selected community. All statistical tests were two-sided.

Supplementary Table 15: Results of the post hoc tests following ANOVA and GLM comparing the soluble phosphate in the nutrient solution per inoculated community (treatment) per timepoint

Effect (days after inoculation)	Contrast	**ANOVA + Tukey** *t* **ratio; p value**	**GLM + EMMs** *t* **ratio; p value**
**Treatment (4)**	PB – C1	1.045; 0.639	-1.257; 0.382
	PR – C1	-0.167; 0.901	-1.304; 0.382
	OBE – C1	0.753; 0.739	-1.024; 0.507
	ORE – C1	0.627; 0.803	-0.792; 0.652
	OBP – C1	3.554; 0.013	4.191; 0.001
	ORP – C1	1.171; 0.585	4.284; <0.001
	PR – PB	-1.213; 0.585	-0.047; 0.963
	OBE – PB	-0.293; 0.900	0.233; 0.904
	ORE – PB	-0.418; 0.839	0.466; 0.846
	OBP – PB	2.509; 0.075	5.448; <0.001
	ORP – PB	0.125; 0.901	5.541; < 0.001
	OBE – PR	0.920; 0.697	0.279; 0.904
	ORE – PR	0.795; 0.739	0.512; 0.846
	OBP – PR	3.722; 0.013	5.495; < 0.001
	ORP – PR	1.388; 0.573	5.588; < 0.001
	ORE – OBE	-0.125; 0.901	0.233; 0.904
	OBP – OBE	2.802; 0.046	5.215; < 0.001
	ORP - OBE	0.418; 0.839	5.308; < 0.001
	OBP – ORE	2.927; 0.045	4.982; < 0.001
	ORP – ORE	0.544; 0.827	5.076; < 0.001
	ORP - OBP	-2.384; 0.083	0.093; 0.963
**Treatment (7)**	PB – C1	-0.206; 0.839	0.206; 0.839
	PR – C1	-1.135; 0.429	-1.135; 0.429
	OBE – C1	-1.573; 0.241	-1.573; 0.241

	ORE – C1	-0.817; 0.589	-0.817; 0.589
	OBP – C1	2.247; 0.068	2.247; 0.068
	ORP – C1	2.693; 0.030	2.693; 0.030
	PR – PB	-0.930; 0.540	-0.930; 0.540
	OBE – PB	-1.367; 0.318	-1.367; 0.318
	ORE – PB	-0.611; 0.674	-0.611; 0.674
	OBP – PB	2.453; 0.047	2.453; 0.047
	ORP – PB	2.898; 0.021	2.898; 0.021
	OBE – PR	-0.438; 0.735	-0.438; 0.735
	ORE – PR	0.319; 0.790	0.319; 0.790
	OBP – PR	3.383; 0.009	3.383; 0.009
	ORP – PR	3.828; 0.004	3.828; 0.004
	ORE – OBE	0.756; 0.598	0.756; 0.598
	OBP – OBE	3.820; 0.004	3.820; 0.004
	ORP - OBE	4.266; 0.004	4.266; 0.004
	OBP – ORE	3.064; 0.016	3.064; 0.016
	ORP – ORE	3.509; 0.008	3.509; 0.008
	ORP - OBP	0.445; 0.735	0.445; 0.735
**Treatment (21)**	PB – C1	1.209; 0.443	1.209; 0.443
	PR – C1	-0.311; 0.796	-0.311; 0.796
	OBE – C1	0.753; 0.597	0.753; 0.597
	ORE – C1	1.094; 0.443	1.094; 0.443
	OBP – C1	3.432; 0.019	3.432; 0.019
	ORP – C1	1.919; 0.194	1.919; 0.194
	PR – PB	-1.520; 0.328	-1.520; 0.328
	OBE – PB	-0.455; 0.761	-0.455; 0.761
	ORE – PB	-0.115; 0.909	-0.115; 0.909
	OBP – PB	2.223; 0.119	2.223; 0.119
	ORP – PB	0.710; 0.597	0.710; 0.597

	OBE – PR	1.065; 0.443	1.065; 0.443
	ORE – PR	1.405; 0.358	1.405; 0.358
	OBP – PR	3.743; 0.016	3.743; 0.016
	ORP – PR	2.230; 0.119	2.230; 0.119
	ORE – OBE	0.341; 0.796	0.341; 0.796
	OBP – OBE	2.679; 0.083	2.679; 0.083
	ORP - OBE	1.165; 0.443	1.165; 0.443
	OBP – ORE	2.338; 0.119	2.338; 0.119
	ORP – ORE	0.824; 0.583	0.824; 0.583
	ORP - OBP	-1.514; 0.328	-1.514; 0.328

C1: uninoculated nutrient solution containing insoluble iron phosphate. PB: parental best-performing community, PR: parental randomly selected community. OBE: environmentally perturbed best-performing community, ORE: environmentally perturbed randomly selected community. OBP: Propagated best-performing community, ORP: propagated randomly selected community. All statistical tests were two-sided and p values were corrected for multiple comparison using the Benjamini-Hochberg procedure.

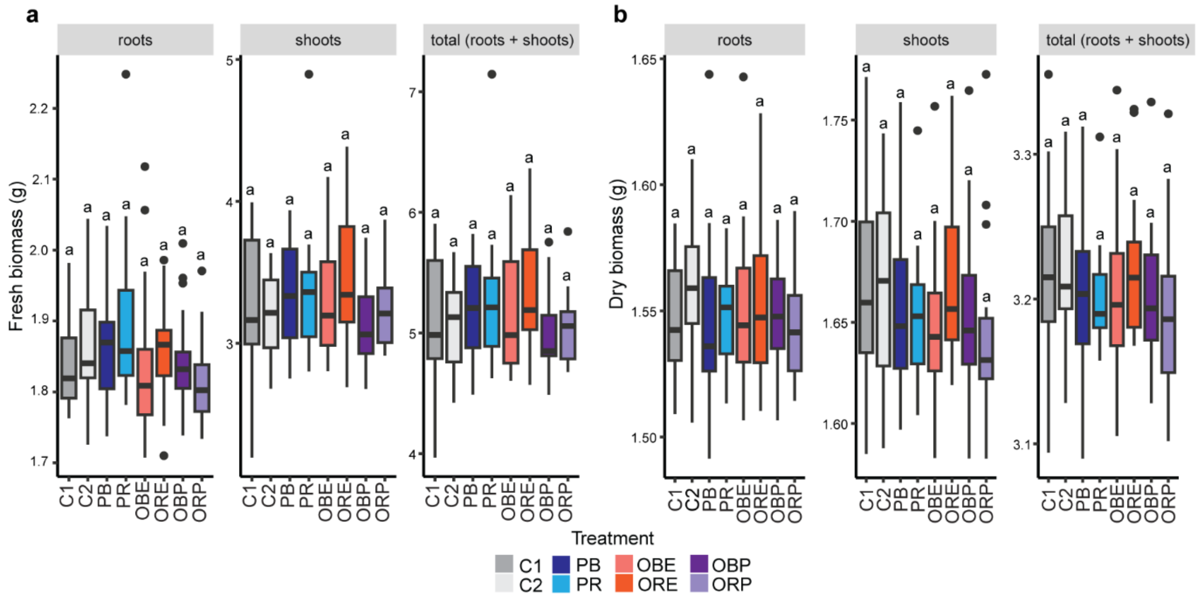

Supplementary Fig. 1: Plant biomass of *Chrysanthemum indicum* L. grown in a hydroponics system in the post-selection experiment. (a) Fresh biomass, and **(b)** dry biomass of Chrysanthemum indicum L. cuttings separated in roots, shoots and total biomass. Community members represented by ASVs and labelled with the most specific known taxonomic level. Box plots: box spans 25th to 75th percentiles (Interquartile Range, IQR) with median as central line, whiskers cover values within 1.5 times IQR, and outliers as dots beyond whiskers. Letters in box plot: groups with different letters are statistically different based on pairwise comparison (p values adjusted via Benjamini-Hochberg procedure). Treatments (for each treatment, n=6 biologically independent replicates): C1: uninoculated, sterile control with nutrient solution containing insoluble $FePO_4 \cdot 2H_2O$. C2: uninoculated, sterile control with nutrient solution containing soluble KH_2PO_4. PB: parental best-performing community, PR: parental randomly selected community. OBE: environmentally perturbed best-performing community, ORE: environmentally perturbed randomly selected community. OBP: Propagated best-performing community, ORP: propagated randomly selected community. All treatments in 6 replicates with 3 cuttings per replicate. Source data are provided as Source Data file.

Supplementary code

```
library(tidyr)
library(dplyr)
library(car)
library(MuMIn)
library(ISLR)
library(ggpmisc)
library(readxl)
library(ExpDes)
library(MASS)
library(sandwich)
library(multcomp)
library(emmeans)


####Statistical analysis Artificial selection of microbial
communities####
#Note: Generation = Phase, Performance = Selection type, Cycle =
Passage, Generation (propagation) = cycle, B2 = propagation, B2B = OBP,
B2R = ORP
#Note: In hydroponics experiment: t1 = 4 days, t2 = 7 days, t3=21 days
after inoculation

####Parental Phase####
PG <- read_excel("PsolubilizationPlant_data.xlsx",
                 sheet = "Parental Generation")
View(PG)

#####Fractions G0C1=PGP1#####
#note; here the factor "Dilution" is the fractions
F_PGP1 <- PG %>%
  filter(Dilution!="S", Dilution!="D0", Dilution!="D1", Dilution!="D2",
Dilution!="Control") %>%
  filter(Cycle=="C1") %>%
  mutate(Dilution=factor(Dilution, levels=c("EE", "HE", "NE"))) %>%
  as.data.frame()

shapiro.test(F_PGP1$ppmpSample) #normally distributed
qqPlot(F_PGP1$ppmpSample)
leveneTest(ppmpSample~Dilution, data = F_PGP1) #assumption of
homogeneity of variances across groups is met
#anova
F_PGP1.anova <- aov(ppmpSample~Dilution, data= F_PGP1)
summary(F_PGP1.anova)
#glm
F_PGP1.glm <- glm(ppmpSample~Dilution, data = F_PGP1)
summary(F_PGP1.glm)
F_PGP1.glm.W <- wald.test(F_PGP1.glm,terms =
length(F_PGP1.glm$coefficients))

#####Dilutions G0C1=PGP1#####
D_PGP1 <- PG %>%
  filter(Dilution!="S", Dilution!="EE", Dilution!="HE", Dilution!="NE",
Dilution!="Control") %>%
  filter(Cycle=="C1") %>%
  mutate(Dilution=factor(Dilution, levels=c("D0", "D1", "D2"))) %>%
  as.data.frame()
```

```
shapiro.test(D_PGP1$ppmpSample) #normally distributed
qqPlot(D_PGP1$ppmpSample)
leveneTest(ppmpSample~Dilution, data = D_PGP1) #assumption of
homogeneity of variances across groups is met
#anova
D_PGP1.anova <- aov(ppmpSample~Dilution, data= D_PGP1)
summary(D_PGP1.anova)
#glm
D_PGP1.glm <- glm(ppmpSample~Dilution, data = D_PGP1)
summary(D_PGP1.glm)
D_PGP1.glm.W <- wald.test(D_PGP1.glm,terms =
length(D_PGP1.glm$coefficients))


#####Whole parental generation = parental phase#####
PG <- read_excel("PsolubilizationPlant_data.xlsx",
                 sheet = "Parental Generation")
View(PG)

PG <- PG %>%
  filter(Dilution!="S", Dilution!="Control") %>%
  mutate(Dilution=factor(Dilution, levels=unique(Dilution))) %>%
  as.data.frame()

shapiro.test(1/(F_totalPG$ppmpSample)) #data not normally distributed
qqPlot(F_totalPG$ppmpSample)
isnt_out_tukey <- function(x, k = 1.5, na.rm = TRUE) {
  quar <- quantile(x, probs = c(0.25, 0.75), na.rm = na.rm)
  iqr <- diff(quar)

  (quar[1] - k * iqr <= x) & (x <= quar[2] + k * iqr)
}
totalPG.stats<-PG %>%
  mutate(is_outlier = !isnt_out_tukey(ppmpSample)) %>%
  filter(is_outlier != "TRUE") %>%
  mutate(Dilution=factor(Dilution, levels= unique(Dilution)))
shapiro.test(totalPG.stats$ppmpSample) #now normally distributed
leveneTest(ppmpSample~Dilution, data = totalPG.stats) #assumption of
homogeneity of variances across groups is met
#anova
totalPG.anova <- aov(ppmpSample~Dilution, data=totalPG.stats)
summary(totalPG.anova)
#glm
totalPG.glm <- glm(ppmpSample~Dilution, data=totalPG.stats, family =
gaussian)
summary(totalPG.glm)
totalPG.glm.W <- wald.test(totalPG.glm,terms =
length(totalPG.glm$coefficients))
#lmer
totalPG.lmer <- lmer(ppmpSample~Dilution*Cycle+(1 | Cycle),
data=totalPG.stats)
summary(totalPG.lmer)
emm_Dilutions <- contrast(emmeans(totalPG.lmer, ~Dilution), "pairwise")
summary(emm_Dilutions, adjust="BH")

####Environmental perturbed offspring phase####
#####Comparison of OBE vs ORE#####
```

```
G1 <- read_excel("PsolubilizationPlant_data.xlsx",
                 sheet = "Stable_Environ.Perturbation")

shapiro.test(((G1_C8C9C10$z_score))) #not normally distributed
qqPlot(G1_C8C9C10$z_score)
isnt_out_tukey <- function(x, k = 1.5, na.rm = TRUE) {
  quar <- quantile(x, probs = c(0.25, 0.75), na.rm = na.rm)
  iqr <- diff(quar)

  (quar[1] - k * iqr <= x) & (x <= quar[2] + k * iqr)
}
G1_C8C9C10.stats <- G1_C8C9C10 %>%
  mutate(zt= z_score+2) %>%
  mutate(is_outlier = !isnt_out_tukey(zt)) %>%
  filter(is_outlier != "TRUE") %>%
  mutate(Performance=factor(Performance)) %>%
  mutate(Treatment=factor(Treatment)) %>%
  data.frame()
shapiro.test(G1_C8C9C10.stats$z_score) #normally distributed
leveneTest(G1_C8C9C10.stats$z_score~G1_C8C9C10.stats$Performance)
#assumption of homogeneity of variances across groups is met

#anova
G1_C8C9C10.anova <- aov(z_score~Performance, data=G1_C8C9C10.stats)
summary(G1_C8C9C10.anova)
#glm
G1_C8C9C10.glm <- glm(z_score~Performance, data=G1_C8C9C10.stats,
family = gaussian)
summary(G1_C8C9C10.glm)
G1_C8C9C10.glm.W <- wald.test(G1_C8C9C10.glm,terms =
length(G1_C8C9C10.glm$coefficients))
#lmer
G1_C8C9C10.lmer <- lmer(z_score~Performance*Cycle+(1 |
Treatment/Cycle), data=G1_C8C9C10.stats)
summary(G1_C8C9C10.lmer)
emm_Performance <- contrast(emmeans(G1_C8C9C10.lmer, ~Performance),
"pairwise")
summary(emm_Performance, adjust="BH")


#####OE vs P######
OE_vs_P <- read_excel("PsolubilizationPlant_data.xlsx",
                      sheet = "Environ.Perturbation_vs_Parent")
OE_vs_P <- OE_vs_P %>%
  mutate(Treatment=factor(Treatment, levels=c("PB", "PR", "OBE",
"ORE"))) %>%
  mutate(Generation=factor(Generation, levels=c("PG", "OG"))) %>%
  mutate(Performance=factor(Performance, levels=c("B", "R"))) %>%
  mutate(Cycle=factor(Cycle)) %>%
  mutate(Replicate=factor(Replicate)) %>%
  mutate(SL=factor(SL))

shapiro.test(OE_vs_P$z_score) #not normally distributed
OE_vs_P.stats <- OE_vs_P %>%
  mutate(zt= z_score+2) %>%
  mutate(is_outlier = !isnt_out_tukey(zt)) %>%
  filter(is_outlier != "TRUE") %>%
  data.frame()
```

```
shapiro.test(OE_vs_P.stats$z_score) #normally distributed
leveneTest(OE_vs_P.stats$z_score~OE_vs_P.stats$Generation) #assumption
of homogeneity of variances across groups is met
leveneTest(OE_vs_P.stats$z_score~OE_vs_P.stats$Performance) #assumption
of homogeneity of variances across groups is met

#anova + tukey
OE_vs_P.anova <- aov(z_score~Generation*Performance,
data=OE_vs_P.stats)
summary(OE_vs_P.anova)
OE_vs_P.anova_Generation_P.Tukey <- glht(model = OE_vs_P.anova,
linfct=mcp(Generation="Tukey"))
summary(OE_vs_P.anova_Generation_P.Tukey, test =adjusted("BH"))
OE_vs_P.anova_Performance_P.Tukey <- glht(model = OE_vs_P.anova,
linfct=mcp(Performance="Tukey"))
summary(OE_vs_P.anova_Performance_P.Tukey, test =adjusted("BH"))
OE_vs_P.anova_tukey_results <- TukeyHSD(OE_vs_P.anova)
p.adjust(OE_vs_P.anova_tukey_results$`Generation:Performance`[,4],
method = "BH",
n=length(OE_vs_P.anova_tukey_results$`Generation:Performance`[,4]))

#glm + emmeans
OE_vs_P.glm <- glm(z_score~Generation*Performance, data=OE_vs_P.stats)
summary(OE_vs_P.glm)
OE_vs_P.glm.W <- wald.test(OE_vs_P.glm,terms =
length(OE_vs_P.glm$coefficients))
OE_vs_P.glm_emm_Generation <- contrast(emmeans(OE_vs_P.glm,
~Generation), "pairwise")
summary(OE_vs_P.glm_emm_Generation, adjust = "BH")
OE_vs_P.glm_emm_Performance <- contrast(emmeans(OE_vs_P.glm,
~Performance), "pairwise")
summary(OE_vs_P.glm_emm_Performance, adjust = "BH")
OE_vs_P.glm_emm_interaction <- contrast(emmeans(OE_vs_P.glm,
~Generation:Performance), "pairwise")
summary(OE_vs_P.glm_emm_interaction, adjust = "BH")

#lmer + emmeans
OE_vs_P.lmer <- lmer(z_score~Generation*Performance*Cycle+(1 |
SL/Cycle), data=OE_vs_P.stats)
summary(OE_vs_P.lmer)
OE_vs_P.lmer_emm_Generation <- contrast(emmeans(OE_vs_P.lmer,
~Generation), "pairwise")
summary(OE_vs_P.lmer_emm_Generation, adjust = "BH")
OE_vs_P.lmer_emm_Performance <- contrast(emmeans(OE_vs_P.lmer,
~Performance), "pairwise")
summary(OE_vs_P.lmer_emm_Performance, adjust = "BH")
OE_vs_P.lmer_emm_interaction <- contrast(emmeans(OE_vs_P.lmer,
~Generation:Performance), "pairwise")
summary(OE_vs_P.lmer_emm_interaction, adjust = "BH")


####Propagation####
OP_vs_P <- read_excel("PsolubilizationPlant_data.xlsx",
                      sheet = "Propagation_vs_Parent")
OP_vs_P <- OP_vs_P %>%
  mutate(Treatment=factor(Treatment, levels=c("PB", "PR", "OBP",
"ORP"))) %>%
  mutate(Generation=factor(Generation, levels=c("PG", "OG"))) %>%
```

```
  mutate(Performance=factor(Performance, levels=c("B", "R"))) %>%
  mutate(Cycle=factor(Cycle)) %>%
  mutate(SL=factor(SL))
shapiro.test(OP_vs_P$z_score) #normally distributed
leveneTest(OP_vs_P$z_score~OP_vs_P$Generation) #assumption of
homogeneity of variances across groups is met
leveneTest(OP_vs_P$z_score~OP_vs_P$Performance) #assumption of
homogeneity of variances across groups is met

#####OP vs P######
#anova + tukey
OP_vs_P.anova <- aov(z_score~Generation*Performance, data=OP_vs_P)
summary(OP_vs_P.anova)
OP_vs_P.anova_Generation_P.Tukey <- glht(model = OP_vs_P.anova,
linfct=mcp(Generation="Tukey"))
summary(OP_vs_P.anova_Generation_P.Tukey, test =adjusted("BH"))
OP_vs_P.anova_Performance_P.Tukey <- glht(model = OP_vs_P.anova,
linfct=mcp(Performance="Tukey"))
summary(OP_vs_P.anova_Performance_P.Tukey, test =adjusted("BH"))
OP_vs_P.anova_tukey_results <- TukeyHSD(OP_vs_P.anova)
OP_vs_P.anova_tukey_results$Performance
p.adjust(OP_vs_P.anova_tukey_results$`Generation:Performance`[,4],
method = "BH",
n=length(OP_vs_P.anova_tukey_results$`Generation:Performance`[,4]))

#glm + emmeans
OP_vs_P.glm <- glm(z_score~Generation*Performance, data=OP_vs_P,
family=gaussian)
summary(OP_vs_P.glm)
with(summary(OP_vs_P.glm), 1-deviance/null.deviance)
OP_vs_P.glm.W <- wald.test(OP_vs_P.glm, terms =
length(OP_vs_P.glm$coefficients))
OP_vs_P.glm_emm_Generation <- contrast(emmeans(OP_vs_P.glm,
~Generation), "pairwise")
summary(OP_vs_P.glm_emm_Generation, adjust="BH")
OP_vs_P.glm_emm_Performance <- contrast(emmeans(OP_vs_P.glm,
~Performance), "pairwise")
summary(OP_vs_P.glm_emm_Performance, adjust="BH")
OP_vs_P.glm_emm_interaction <- contrast(emmeans(OP_vs_P.glm,
~Generation:Performance), "pairwise")
summary(OP_vs_P.glm_emm_interaction, adjust = "BH")

#lmer + emmeans
OP_vs_P.lmer <- lmer(z_score~Generation*Performance*Cycle+(1 | Cycle),
data=OP_vs_P)
summary(OP_vs_P.lmer)
OP_vs_P.lmer_emm_Generation <- contrast(emmeans(OP_vs_P.lmer,
~Generation), "pairwise")
summary(OP_vs_P.lmer_emm_Generation, adjust = "BH")
OP_vs_P.lmer_emm_Performance <- contrast(emmeans(OP_vs_P.lmer,
~Performance), "pairwise")
summary(OP_vs_P.lmer_emm_Performance, adjust = "BH")
OP_vs_P.lmer_emm_interaction <- contrast(emmeans(OP_vs_P.lmer,
~Generation:Performance), "pairwise")
summary(OP_vs_P.lmer_emm_interaction, adjust = "BH")


####Comparison of environmental perturabtion and propagation####
```

```
OE_vs_OP <- read_excel("PsolubilizationPlant_data.xlsx",
                       sheet = "Environ.Perturb._vs_Propagation")
OE_vs_OP <- OE_vs_OP %>%
  mutate(Treatment=factor(Treatment, levels=c("OBE", "ORE", "OBP",
"ORP"))) %>%
  mutate(Method=factor(Method, levels=c("E", "P"))) %>%
  mutate(Performance=factor(Performance, levels=c("B", "R"))) %>%
  mutate(Cycle=factor(Cycle)) %>%
  mutate(SL=factor(SL))
shapiro.test((OE_vs_OP$z_score)) #not normally distributed
OE_vs_OP.stats <- OE_vs_OP %>%
  mutate(zt= z_score+2) %>%
  mutate(is_outlier = !isnt_out_tukey(zt)) %>%
  filter(is_outlier != "TRUE") %>%
  data.frame()
shapiro.test((OE_vs_OP.stats$z_score)) #not normally distributed
shapiro.test(sqrt(OE_vs_OP.stats$zt)) #normally distributed
leveneTest(sqrt(OE_vs_OP.stats$zt)~OE_vs_OP.stats$Method) #assumption
of homogeneity of variances across groups is met
leveneTest(sqrt(OE_vs_OP.stats$zt)~OE_vs_OP.stats$Performance)
#assumption of homogeneity of variances across groups is met

#anova + tukey
OE_vs_OP.anova <- aov(sqrt(zt)~Method*Performance, data=OE_vs_OP.stats)
summary(OE_vs_OP.anova)
OE_vs_OP.anova_Method_P.Tukey <- glht(model = OE_vs_OP.anova,
linfct=mcp(Method="Tukey"))
summary(OE_vs_OP.anova_Method_P.Tukey, test =adjusted("BH"))
OE_vs_OP.anova_Performance_P.Tukey <- glht(model = OE_vs_OP.anova,
linfct=mcp(Performance="Tukey"))
summary(OE_vs_OP.anova_Performance_P.Tukey, test =adjusted("BH"))
OE_vs_OP.anova_tukey_results <- TukeyHSD(OE_vs_OP.anova)
p.adjust(OE_vs_OP.anova_tukey_results$`Method:Performance`[,4], method
= "BH",
n=length(OE_vs_OP.anova_tukey_results$`Method:Performance`[,4]))

#glm + emmeans
OE_vs_OP.glm <- glm(sqrt(zt)~Method*Performance, data=OE_vs_OP.stats)
summary(OE_vs_OP.glm)
OE_vs_OP.glm.W <- wald.test(OE_vs_OP.glm, terms =
length(OE_vs_OP.glm$coefficients))
OE_vs_OP.glm_emm_Method <- contrast(emmeans(OE_vs_OP.glm, ~Method),
"pairwise")
summary(OE_vs_OP.glm_emm_Method, adjust="BH")
OE_vs_OP.glm_emm_Performance <- contrast(emmeans(OE_vs_OP.glm,
~Performance), "pairwise")
summary(OE_vs_OP.glm_emm_Performance, adjust="BH")
OE_vs_OP.glm_emm_interaction <- contrast(emmeans(OE_vs_OP.glm,
~Method:Performance), "pairwise")
summary(OE_vs_OP.glm_emm_interaction, adjust = "BH")

#LMER + emmeans
OE_vs_OP.lmer <- lmer(sqrt(zt)~Method*Performance*Cycle+(1 | SL/Cycle),
data=OE_vs_OP.stats)
summary(OE_vs_OP.lmer)
OE_vs_OP.lmer_emm_Method <- contrast(emmeans(OE_vs_OP.lmer, ~Method),
"pairwise")
summary(OE_vs_OP.lmer_emm_Method, adjust = "BH")
```

```
OE_vs_OP.lmer_emm_Performance <- contrast(emmeans(OE_vs_OP.lmer,
~Performance), "pairwise")
summary(OE_vs_OP.lmer_emm_Performance, adjust = "BH")
OE_vs_OP.lmer_emm_interaction <- contrast(emmeans(OE_vs_OP.lmer,
~Method:Performance), "pairwise")
summary(OE_vs_OP.lmer_emm_interaction, adjust = "BH")


####Hydroponics experiment####
#####Plant biomass######
G_biomass <- read_excel("Plant experiment.excel",
                        sheet = "Chrysanthemum_Biomass")

#####Plant biomass total######
total <- G_biomass %>%
  filter(Part == "total") %>%
  mutate(Treatment = factor(Treatment)) %>%
  mutate(Replicate = substr(SampleID, nchar(SampleID)-1,
nchar(SampleID))) %>%
  mutate(is_outlier = !isnt_out_tukey(`Dry weight`)) %>%
  filter(is_outlier != "TRUE") %>%
  mutate(is_outlier2 = !isnt_out_tukey(`Fresh weight`)) %>%
  filter(is_outlier2 != "TRUE") %>%
  data.frame()
shapiro.test((total$Dry.weight)) #normally distributed
shapiro.test(1/(total$Fresh.weight)) #normally distributed
leveneTest((total$Dry.weight)~total$Treatment) #assumption of
homogeneity of variances across groups is met
leveneTest(1/(total$Fresh.weight)~total$Treatment) #assumption of
homogeneity of variances across groups is met

#anova
rbd(total$Treatment, factor(total$Replicate),
    ((total$Dry.weight)), quali = TRUE, sigT = 0.05,sigF = 0.05)
rbd(total$Treatment, factor(total$Replicate),
    (1/(total$Fresh.weight)), quali = TRUE, sigT = 0.05,sigF = 0.05)

#glm
total.glm <- glm(Dry.weight~Treatment+factor(Replicate), data = total)
summary(total.glm)
total.glmW <- wald.test(total.glm, terms =
length(total.glm$coefficients))
total.glm <- glm(1/(Fresh.weight)~Treatment+factor(Replicate), data =
total)
summary(total.glm)
total.glmW <- wald.test(total.glm, terms =
length(total.glm$coefficients))

#####Plant biomass shoots######
shoots <- G_biomass %>%
  filter(Part == "shoots") %>%
  mutate(Treatment = factor(Treatment)) %>%
  mutate(Replicate = substr(SampleID, nchar(SampleID)-1,
nchar(SampleID))) %>%
  mutate(is_outlier = !isnt_out_tukey(`Dry weight`)) %>%
  filter(is_outlier != "TRUE") %>%
  mutate(is_outlier2 = !isnt_out_tukey(`Fresh weight`)) %>%
  filter(is_outlier2 != "TRUE") %>%
```

```
  data.frame()
shapiro.test(1/(shoots$Dry.weight)) #normally distributed
shapiro.test(log(shoots$Fresh.weight)) #normally distributed
leveneTest(1/(shoots$Dry.weight)~shoots$Treatment) #assumption of
homogeneity of variances across groups is met
leveneTest(log(shoots$Fresh.weight)~shoots$Treatment) #assumption of
homogeneity of variances across groups is met

#anova
rbd(shoots$Treatment, factor(shoots$Replicate),
    (log(shoots$Fresh.weight)), quali = TRUE, sigT = 0.1,sigF = 0.1)
rbd(shoots$Treatment, factor(shoots$Replicate),
    (1/(shoots$Dry.weight)), quali = TRUE, sigT = 0.1,sigF = 0.1)

#glm
shoots.glm <- glm(log(Fresh.weight)~Treatment+factor(Replicate), data =
shoots)
summary(shoots.glm)
shoots.glm <- glm(1/(Dry.weight)~Treatment+factor(Replicate), data =
shoots)
summary(shoots.glm)
shoots.glmW <- wald.test(shoots.glm, terms =
length(shoots.glm$coefficients))
shoots.glm_emm_Treatment <- contrast(emmeans(shoots.glm, ~Treatment),
"pairwise")
summary(shoots.glm_emm_Treatment, adjust="BH")

#######Plant biomass roots########
roots <- G_biomass %>%
  filter(Part == "roots") %>%
  mutate(Treatment = factor(Treatment)) %>%
  mutate(Replicate = substr(SampleID, nchar(SampleID)-1,
nchar(SampleID))) %>%
  mutate(is_outlier = !isnt_out_tukey(`Dry weight`)) %>%
  filter(is_outlier != "TRUE") %>%
  mutate(is_outlier2 = !isnt_out_tukey(`Fresh weight`)) %>%
  filter(is_outlier2 != "TRUE") %>%
  mutate(Treatment = relevel(Treatment, ref = "C1")) %>%
  data.frame()
shapiro.test(1/(roots$Dry.weight)) #normally distributed
shapiro.test(1/(roots$Fresh.weight)) #normally distributed
leveneTest(1/(roots$Dry.weight)~roots$Treatment) #assumption of
homogeneity of variances across groups is met
leveneTest(1/(roots$Fresh.weight)~roots$Treatment) #assumption of
homogeneity of variances across groups is met

#anova
rbd(roots$Treatment, factor(roots$Replicate),
    (1/(roots$Fresh.weight)), quali = TRUE, sigT = 0.1,sigF = 0.1)
rbd(roots$Treatment, factor(roots$Replicate),
    (1/(roots$Dry.weight)), quali = TRUE, sigT = 0.1,sigF = 0.1)

#glm
roots.glm <- glm(1/(Fresh.weight)~Treatment+factor(Replicate), data =
roots)
summary(roots.glm)
roots.glm.W <- wald.test(roots.glm, terms =
length(roots.glm$coefficients))
```

```
roots.glm <- glm(1/(Dry.weight)~Treatment+factor(Replicate), data =
roots)
summary(roots.glm)
roots.glm <- wald.test(roots.glm, terms =
length(roots.glm$coefficients))


#####Hoagland solution#####
HSG <- read_excel("PsolubilizationPlant_data.xlsx",
                        sheet = "Chrysanthemum_HoaglandSolution")
###4 days###
data.HSG.t1 <- HSG %>%
  group_by(Treatment,Time) %>%
  filter(Treatment!="C2") %>%
  mutate(ppmpt = ppmp+1) %>%
  mutate(Treatment=factor(Treatment, levels=c("C1", "PB", "PR", "OB",
"OR", "B2B", "B2R"))) %>%
  mutate(Generation=factor(Generation, levels=c("C1", "P", "OE",
"OP"))) %>%
  mutate(Performance=factor(Performance, levels=c("C1", "B", "R"))) %>%
  mutate(Time=factor(Time, levels=c("t1", "t2", "t3"))) %>%
  filter(Time== "t1")

shapiro.test((data.HSG.t1$ppmpt)) #normally distributed
qqPlot(log(data.HSG$ppmpt))
leveneTest((data.HSG.t1$ppmpt)~data.HSG.t1$Treatment) #assumption of
homogeneity of variances across groups is met

#anova + tukey
HSG.t1.anova <- aov(ppmpt~Treatment+factor(Replicate), data = data.HSG)
summary(HSG.t1.anova)
HSG.t1.anova_P.Tukey <- glht(model = HSG.t1.anova,
linfct=mcp(Treatment="Tukey"))
summary(HSG.t1.anova_P.Tukey, test =adjusted("BH"))

#glm + emmeans
HSG.t1.glm <- glm(ppmpt~Treatment+factor(Replicate), data =
data.HSG.t1)
summary(HSG.t1.glm)
HSG.t1.glmW <- wald.test(HSG.t1.glm, terms =
length(HSG.t1.glm$coefficients))
HSG.t1.glm_emm_Treatment <- contrast(emmeans(HSG.t1.glm, ~Treatment),
"pairwise")
summary(emm_Treatment, adjust="none")

###7d###
data.HSG.t2 <- HSG %>%
  group_by(Treatment,Time) %>%
  filter(Treatment!="C2") %>%
  mutate(ppmpt = ppmp+1) %>%
  mutate(Treatment=factor(Treatment, levels=c("C1", "PB", "PR", "OB",
"OR", "B2B", "B2R"))) %>%
  mutate(Generation=factor(Generation, levels=c("C1", "P", "OE",
"OP"))) %>%
  mutate(Performance=factor(Performance, levels=c("C1", "B", "R"))) %>%
  mutate(Time=factor(Time, levels=c("t1", "t2", "t3"))) %>%
  filter(Time== "t2")
```

```
shapiro.test(log(data.HSG.t2$ppmpt)) #normally distributed
qqPlot(log(data.HSG.t2$ppmpt))
leveneTest(log(data.HSG.t2$ppmpt)~data.HSG.t2$Treatment) #assumption of
homogeneity of variances across groups is met

#anova + tukey
HSG.t2.anova <- aov((log(ppmpt))~Treatment+factor(Replicate), data =
data.HSG.t2)
summary(HSG.t2.anova)
HSG.t2.anova_P.Tukey <- glht(model = HSG.t2.glm,
linfct=mcp(Treatment="Tukey"))
summary(HSG.t2.anova_P.Tukey, test =adjusted("BH"))
cld(HSG.t2.anova_P.Tukey, decreasing = TRUE)

#glm + emmeans
HSG.t2.glm <- glm((log(ppmpt))~Treatment+factor(Replicate), data =
data.HSG.t2)
summary(HSG.t2.glm)
HSG.t2.glmW <- wald.test(HSG.t2.glm, terms =
length(HSG.t2.glm$coefficients))
HSG.t2.glm_emm_Treatment <- contrast(emmeans(HSG.t2.glm, ~Treatment),
"pairwise")
summary(emm_Treatment, adjust="BH")

###21 days###
data.HSG.t3 <- HSG %>%
  group_by(Treatment,Time) %>%
  filter(Treatment!="C2") %>%
  mutate(ppmpt = ppmp+1) %>%
  mutate(Treatment=factor(Treatment, levels=c("C1", "PB", "PR", "OB",
"OR", "B2B", "B2R"))) %>%
  mutate(Generation=factor(Generation, levels=c("C1", "P", "OE",
"OP"))) %>%
  mutate(Performance=factor(Performance, levels=c("C1", "B", "R"))) %>%
  mutate(Time=factor(Time, levels=c("t1", "t2", "t3"))) %>%
  filter(Time== "t3")
shapiro.test(1/(data.HSG.t3$ppmpt)) #normally distributed
qqPlot(1/(data.HSG.t3$ppmpt))
leveneTest(1/(data.HSG.t3$ppmpt)~data.HSG.t3$Treatment) #assumption of
homogeneity of variances across groups is met

#anova + tukey
HSG.t3.anova <- aov((1/(ppmpt))~Treatment+factor(Replicate), data =
data.HSG.t3)
summary(HSG.t3.anova)
HSG.t3.anova_P.Tukey <- glht(model = HSG.t3.anova,
linfct=mcp(Treatment="Tukey"))
summary(HSG.t3.anova_P.Tukey, test =adjusted("BH"))
cld(HSG.t3.anova_P.Tukey, decreasing = TRUE)

#glm + emmeans
HSG.t3.glm <- glm((1/(ppmpt))~Treatment+factor(Replicate), data =
data.HSG.t3)
summary(HSG.t3.glm)
HSG.t3.glmW <- wald.test(HSG.t3.glm, terms =
length(HSG.t3.glm$coefficients))
HSG.t3.glm_emm_Treatment <- contrast(emmeans(HSG.t3.glm, ~Treatment),
"pairwise")
```

```
summary(emm_Treatment, adjust="BH")

########GJAM for analysis of sequences##########
#####GJAM for Fractions#######
library(dplyr)
library(tidyr)

####treating of PS###########
#7000taxa if we need all of them so we check

apply(tax_table(ps), 2, unique)
lapply(apply(tax_table(ps), 2, unique), length)
#Kingdom: 4
#Phylum: 37
#Class: 95
#Order: 182
#Family: 234
#Genus: 429
#Species:96

#filter from Kingoms
ps1<-subset_taxa(ps, Kingdom %in% c("Bacteria", "Archaea"))
apply(tax_table(ps1), 2, unique)

#filter further: NA in phylum, "Cyanobacteria" because they are likely
to be chloroplasts
ps2<-subset_taxa(ps1, Phylum!="Cyanobacteria")
ps3<-subset_taxa(ps2, !is.na(Phylum))

apply(tax_table(ps3), 2, unique)
lapply(apply(tax_table(ps3), 2, unique), length)
#Kingdom: 2
#Phylum: 35
#Class: 92
#Order: 178
#Family: 231
#Genus: 426
#Species:94

#different experiment ps data
ps_Fractions <- subset_taxa(ps3, Experiment=="F")
lapply(apply(tax_table(ps_Fractions), 2, unique), length)
#Kingdom: 2
#Phylum: 23
#Class: 57
#Order: 108
#Family: 135
#Genus: 173
#Species:26


pdata<-tax_glom(ps3, taxrank="Genus", NArm=FALSE)
View(data.frame(tax_table(pdata)))

#######GJAM modelling Fractions & parental phase######
library(gjam)

xdata  <- data.frame(sample_data(pdata)) %>%
```

```
  filter(Experiment == "F") %>%
  dplyr::select(-Timepoint) %>%
  dplyr::rename(Fraction = Performance)

otu    <- data.frame(otu_table(pdata)) %>%
  filter(Experiment == "F")


TaxInfo<-data.frame(tax_table(pdata)) %>%
  unite("GenInfor", Kingdom:Genus, sep="_", remove=FALSE) %>%
  mutate(GenCode=paste0("Tax", 1:nrow(. )))
colnames(otu)<-TaxInfo$GenCode
View(otu)

hist( as.matrix(otu), ylab = "Reads", main = "OTU's")
#histogram looks weird HELP

min.ob<-ifelse(nrow(otu)*0.05<5,5, round(nrow(otu)*0.05,0))
nobs <- gjamTrimY( otu, minObs = min.ob, OTHER = F )$nobs   #minObs
either 5 or 5% of samples
hist( nobs/ncol(otu), nclass=100, xlab = 'Fraction of observations',
      ylab = 'Frequency', main='Incidence' ) #how common to find in
relation to all number of species



#trim by abundance instead of number of observations
tmp <- gjamTrimY(otu, minObs = min.ob)
y   <- tmp$y

TaxInfo %>%
  filter(!GenCode %in% colnames(y)) %>%
  apply(.,2,unique)

dim(otu)                      # all OTUs; 701
dim(y)                        # trimmed data; 10
tail(colnames(y))             # 'other' class added

#type names to microbial composition
ppmp  <- as.vector(xdata$ppmp)
ydata <- cbind(y, ppmp)
head(ydata)
S     <- ncol(ydata)
typeNames    <- c(rep('CC',S-1), "CON")   # composition count data and
ppmp as continuous data

#set level for xdata; density
xdata$Fraction<-factor(xdata$Fraction, levels = c("EE","HE", "NE"))

#run model
ml <- list( ng = 10000, burnin = 4000, typeNames = typeNames, REDUCT =
F)
output <- gjam(~Fraction, xdata, ydata, modelList = ml)
#1st variable is intercept, Performance
#warning message
#View(otu)
#check GJAM output with
View(output$parameters$betaTable)
```

```
#check model fit
fit <- gjamPlot(output_Fractions, plotPars = list(GRIDPLOTS=T,
SAVEPLOTS=TRUE))
#Warning messages:
#1: In min(x) : no non-missing arguments to min; returning Inf
#2: In max(x) : no non-missing arguments to max; returning -Inf

output_Fractions<-output
fit_Fractions<-fit


###diagnosis of MCMC chains to see if model is okay to continue

#Add here your settings for Gibbs sampling
ng <- 10000
burning <- 4000

#Add here you output from the GJAM analysis
selected.model <- output_Fractions

plot(cumsum(selected.model$chains$bgibbs[,1]-
mean(selected.model$chains$bgibbs[,1])),
     type="l", xlab="iteration", ylab="y")
plot(selected.model$chains$bgibbsUn[,1]-
mean(selected.model$chains$bgibbsUn[,1]),
     type="l", xlab="iteration", ylab="y")

plot(selected.model$chains$bgibbs[,1])

# center with 'apply()'
center_apply <- function(x) {
  apply(x, 2, function(y) y - mean(y))
}

# apply it
dfplot <-
data.frame(center_apply(data.frame(selected.model$chains$bgibbs)))
#dfplot <-
data.frame(center_apply(data.frame(selected.model$chains$bgibbs[burning
:ng,])))
#dfplot <-
data.frame(center_apply(data.frame(selected.model$chains$sgibbs)))

plot(dfplot[,5],type="l", xlab="iteration", ylab="y")
dim(dfplot)

#bgibbs
DiagTab <- dfplot %>%
  #  data.frame() %>%
  mutate(Iteration = 1:nrow(dfplot)) %>%
  gather(key = "Parameter", value = "value", -Iteration) %>%
  # filter(Iteration>burning) %>%
  group_by(Parameter) %>%
  summarise(Median = mean(value, na.rm = TRUE), CIlow = quantile(value,
0.025, na.rm = TRUE),
            CIhigh = quantile(value, 0.975, na.rm = TRUE),IQR =
IQR(value)) %>%
```

```
  ungroup() %>%
  filter(IQR>quantile(IQR,0.975)) %>% #Q3
  # filter(IQR>10) %>%
  separate(Parameter, into = c("Taxa","Treatment"),sep = "_", remove =
FALSE)

#sgibbs
#DiagTab <- dfplot %>%
#  data.frame() %>%
# mutate(Iteration = 1:nrow(dfplot)) %>%
#gather(key = "Parameter", value = "value", -Iteration) %>%
#filter(Iteration>burning) %>%
#group_by(Parameter) %>%
#summarise(Median = mean(value, na.rm = TRUE), CIlow = quantile(value,
0.025, na.rm = TRUE),
#           CIhigh = quantile(value, 0.975, na.rm = TRUE),IQR =
IQR(value)) %>%
# ungroup() %>%
#filter(IQR>quantile(IQR,0.975))

dim(DiagTab)
View(DiagTab)    #DiagTab gives the samples with the highest divergent
summary(DiagTab)

summary(DiagTab$IQR)

hist(DiagTab$IQR)
boxplot(DiagTab$IQR)     #important to see in which range the variation
happens; if small go on
length(unique(DiagTab$Parameter))
Notconverged <- unique(DiagTab$Parameter)
unique(DiagTab$Taxa) #?

plot(dfplot[,Notconverged[1]]-mean(dfplot[,Notconverged[1]]),type="l",
xlab="iteration", ylab="y")
plot(cumsum(dfplot[,Notconverged[1]]),type="l", xlab="iteration",
ylab="y")

View(selected.model$inputs$y[,unique(DiagTab$Taxa)])
heatmap(scale(selected.model$inputs$y[,unique(DiagTab$Taxa)]))
apply(selected.model$inputs$y[,unique(DiagTab$Taxa)],2,hist)

library(ggplot2)
#Looking to the chains that did not converged
dfplot %>%
  # mutate_all(list(cumsum)) %>%
  #  data.frame() %>%
  mutate(ng = 1:nrow(dfplot)) %>%
  # filter(ng>burning) %>%
  tidyr::gather(key = VarName, value = MCMC, -ng) %>%
  # filter(VarName %in% sample(Notconverged,20)) %>%
  filter(VarName %in% Notconverged) %>% #If smaller than 20
  ggplot(aes(x = ng, y = MCMC, color = VarName))+
  geom_line()+
  # scale_y_log10()+
  theme(panel.grid.major = element_blank(), panel.grid.minor =
element_blank(), panel.background = element_blank(), axis.line =
element_line(colour="black"))
```

```
#Randomly checking the converged chains
SampleRandom <- sample(colnames(dfplot),20)
dfplot %>%
  #  data.frame() %>%
  mutate(ng = 1:nrow(dfplot)) %>%
  filter(ng>burning) %>%
  tidyr::gather(key = VarName, value = MCMC, -ng) %>%
  filter(VarName %in% SampleRandom) %>%
  ggplot(aes(x = ng, y = MCMC, color = VarName))+
  geom_line()+
  theme(panel.grid.major = element_blank(), panel.grid.minor =
element_blank(), panel.background = element_blank(), axis.line =
element_line(colour="black"))
#theme(legend.position = "none")

dfplot %>%
  #  data.frame() %>%
  mutate(ng = 1:nrow(dfplot)) %>%
  filter(ng>burning) %>%
  tidyr::gather(key = VarName, value = MCMC, -ng) %>%
  filter(VarName %in% SampleRandom) %>%
  ggplot(aes(x = VarName, y = MCMC, color = VarName))+
  geom_boxplot()+
  theme(legend.position = "none")

#if stable save data for next step
save(output_Fractions,file="gjamOutput_Fractions.RData")

####ONE WAY ANALYSIS####
#Gjam standard plot

pl  <- list(SAVEPLOTS = TRUE, GRIDPLOTS =TRUE)
gjamPlot( output_Fractions, plotPars = pl)
selected.model <- output_Fractions
selected.model$inputs$y
ynames <- colnames(selected.model$inputs$y)
ynames
View(TaxInfo[TaxInfo$GenCode %in% ynames,])

#Coefficients
heatmap(selected.model$parameters$betaStandXWmu)
library("gplots")
netTab <- selected.model$parameters$betaStandXWmu[,1:10]
hT <- heatmap.2(netTab, scale = "column", col = rev(bluered(100)),
cexRow =0.6, cexCol=1, #lhei=lhei, lwid=lwid,lmat =lmat,
                trace = "none", density.info = "none", labCol =
LabelsCode$AbbTax)


LabelsCode <- data.frame(GenCode = colnames(netTab)) %>%
  left_join(TaxInfo,by = c("GenCode")) %>%
  mutate(AbbTax = paste(Genus)) %>%
  mutate(AbbTax = gsub("NA","un",AbbTax)) %>%
  mutate(AbbTax = ifelse(AbbTax == "un", paste(Family), AbbTax)) %>%
  mutate(AbbTax = ifelse(AbbTax == "NA", paste(Order), AbbTax)) %>%
  mutate(AbbTax = ifelse(AbbTax == "NA", paste(Class), AbbTax)) %>%
```

```
  mutate(AbbTax = ifelse(AbbTax == "NA", paste(Phylum), AbbTax)) %>%
  mutate(AbbTax=ifelse(GenCode=="ppmp", paste("soluble P"), AbbTax))

dendVar <- hT$colDendrogram
labels(dendVar) <- LabelsCode$AbbTax
plot(as.hclust(dendVar), hang = -1, cex = 0.6)



#Null significant effect
coeffTabfinal <- selected.model$parameters$betaStandXWTable
NullSigEff <- coeffTabfinal %>%
  mutate(VarEffect =
rownames(selected.model$parameters$betaStandXWTable)) %>%
  tidyr::separate(VarEffect, into = c("DepVar", "Treat"), sep = "_")
%>%
  #tidyr::separate(DepVar, into = c("Phylum", "Class")) %>%
  # mutate(DepVar = ifelse(DepVar == "HAl", "H_Al", DepVar)) %>% #View
  mutate(DepVar = factor(DepVar, levels = unique(DepVar))) %>%
  filter(sig95 == "*")

View(NullSigEff)

LabelsCode.sig<-LabelsCode %>%
  filter(GenCode %in% NullSigEff$DepVar)

unique(NullSigEff$DepVar)
heatmap.2(netTab[,unique(NullSigEff$DepVar)], scale = "column", col =
rev(bluered(100)),cexCol = 1, cexRow =1,#lhei=lhei, lwid=lwid,lmat
=lmat,
          trace = "none", density.info = "none", labCol =
LabelsCode.sig$AbbTax)

###Bubble Plot###
library(zCompositions)
selected.model<-output_Fractions
ydata<-selected.model$inputs$y
d.czm <- cmultRepl(ydata[,1:9],  label=0, method="CZM")
colnames(d.czm)
# The table needs to be transposed again (samples as COLUMNS)
#log - log means division ; relative abudnance
d.clr <- t(apply(d.czm, 1, function(x){log(x) - mean(log(x))}))

ControlData <- cbind(output_Fractions$inputs$xdata, d.clr) %>% #View
  gather(key = "GenCode", value = "CLR",
contains("Tax"),contains("other")) %>%
  left_join(TaxInfo, by = "GenCode") %>% #View
  rename(Fraction=Performance) %>%
  filter(Fraction=="EE") %>%
  mutate(AbbTax = paste0(Genus,Species)) %>%
  mutate(AbbTax = gsub("g__","",AbbTax)) %>%
  mutate(AbbTax = gsub("s__"," ",AbbTax)) %>%
  mutate(AbbTax = gsub("NA"," un",AbbTax)) %>%
  mutate(AbbTax = ifelse(AbbTax == " un un", GenCode, AbbTax)) %>%
  dplyr::select(-SampleID) %>% #colnames()
  group_by(Kingdom, Phylum, Class, Order, Family, Genus, Species,
AbbTax,Fraction,  GenCode) %>%
  summarise(CLR.mean = mean(CLR)) %>%
```

```
  ungroup() %>%
  mutate(Fraction = gsub("S", "", Fraction)) %>%
  rename(DepVar=GenCode)
View(ControlData)


#Buble plot
#RtN0
#t(EstimateEffRtN0[,-c(1,8)])
CoeffValues <- NullSigEff %>%
  dplyr::select(Estimate,DepVar, Treat) %>%
  mutate(Treatment = gsub("Fraction", "", Treat)) %>%
  mutate(Treatment = gsub("Performance", "", Treat)) %>%
  filter(Treat != "PerformanceEE")


CoeffValues %>%

  mutate(Size = ifelse(Estimate<0, -1*Estimate,Estimate)) %>%
  left_join(ControlData, by = "DepVar") %>% #View

  mutate(NameG = ifelse(Estimate %in%
tail(sort(CoeffValues$Estimate),5), AbbTax, NA)) %>% #View
  mutate(NameG = ifelse(Estimate %in%
head(sort(CoeffValues$Estimate),5), AbbTax, NameG)) %>% #View
  mutate(Treatment = factor(Treatment, levels = c("HE", "NE"))) %>%
  ggplot(aes(x=CLR.mean, y=Estimate, size=Size,
             color=NameG)) +
  geom_point(alpha=0.5) +
  #geom_text(aes(label=NameG, size = 3),hjust=0.1, vjust=0.1)+
  facet_wrap(~Treatment, scales = "free")+
  geom_hline(yintercept = 0, linetype = "dashed")+
  geom_vline(xintercept = 0, linetype = "dashed")+
  theme_bw()

#####GJAM Dilutions######
####treating of PS###########
#7000taxa if we need all of them so we check

apply(tax_table(ps), 2, unique)
lapply(apply(tax_table(ps), 2, unique), length)
#Kingdom: 4
#Phylum: 37
#Class: 95
#Order: 182
#Family: 234
#Genus: 429
#Species:96

#filter from Kingoms
ps1<-subset_taxa(ps, Kingdom %in% c("Bacteria", "Archaea"))
apply(tax_table(ps1), 2, unique)

#filter further: NA in phylum, "Cyanobacteria" because they are likely
to be chloroplasts
ps2<-subset_taxa(ps1, Phylum!="Cyanobacteria")
ps3<-subset_taxa(ps2, !is.na(Phylum))

```

```
apply(tax_table(ps3), 2, unique)
lapply(apply(tax_table(ps3), 2, unique), length)
#Kingdom: 2
#Phylum: 35
#Class: 92
#Order: 178
#Family: 231
#Genus: 426
#Species:94

#different experiment ps data


ps_Dilutions <- subset_taxa(ps3, Experiment=="D")
lapply(apply(tax_table(ps_Dilutions), 2, unique), length)
#Kingdom: 2
#Phylum: 27
#Class: 59
#Order: 106
#Family: 136
#Genus: 186
#Species:27




pdata<-tax_glom(ps3, taxrank="Genus", NArm=FALSE)
View(data.frame(tax_table(pdata)))

#######GJAM modelling Dilutions######
library(gjam)

xdata  <- data.frame(sample_data(pdata)) %>%
  filter(Experiment == "D") %>%
  dplyr::select(-Timepoint)

otu    <- data.frame(otu_table(pdata)) %>%
  filter(Experiment == "D")


TaxInfo<-data.frame(tax_table(pdata)) %>%
  unite("GenInfor", Kingdom:Genus, sep="_", remove=FALSE) %>%
  mutate(GenCode=paste0("Tax", 1:nrow(. )))
colnames(otu)<-TaxInfo$GenCode
View(otu)

hist( as.matrix(otu), ylab = "Reads", main = "OTU's")
#histogram looks weird HELP

min.ob<-ifelse(nrow(otu)*0.05<5,5, round(nrow(otu)*0.05,0))
nobs <- gjamTrimY( otu, minObs = min.ob, OTHER = F )$nobs   #minObs
either 5 or 5% of samples
hist( nobs/ncol(otu), nclass=100, xlab = 'Fraction of observations',
      ylab = 'Frequency', main='Incidence' ) #how common to find in
relation to all number of species
```

```
#trim by abundance instead of number of observations
tmp <- gjamTrimY(otu, minObs = min.ob)
y   <- tmp$y

TaxInfo %>%
  filter(!GenCode %in% colnames(y)) %>%
  apply(.,2,unique)

dim(otu)                      # all OTUs; 701
dim(y)                        # trimmed data; 8
tail(colnames(y))             # 'other' class added

#type names to microbial composition
ppmp  <- as.vector(xdata$ppmp)
ydata <- cbind(y, ppmp)
head(ydata)
S     <- ncol(ydata)
typeNames    <- c(rep('CC',S-1), "CON")   # composition count data and
ppmp as continuous data

#set level for xdata; density
xdata<-xdata %>%
  rename(Dilution = Performance)
xdata$Dilution<-factor(xdata$Dilution, levels = c("D0","D1", "D2"))

#run model
ml <- list( ng = 10000, burnin = 4000, typeNames = typeNames, REDUCT =
F)
output <- gjam(~ Dilution, xdata, ydata, modelList = ml)
#1st variable is intercept, Perfromance(=Dilution)
#warning message:
#In .setupReduct(modelList, S, Q, n) : dimension reduction
#check GJAM output with
View(output$parameters$betaTable)

#check model fit
fit <- gjamPlot(output, plotPars = list(GRIDPLOTS=T, SAVEPLOTS=TRUE) )


output_Dilutions<-output
fit_Dilutions<-fit

###diagnosis of MCMC chains to see if model is okay to continue####

#Add here your settings for Gibbs sampling
ng <- 10000
burning <- 4000

#Add here you output from the GJAM analysis
selected.model <- output_Dilutions

plot(cumsum(selected.model$chains$bgibbs[,1]-
mean(selected.model$chains$bgibbs[,1])),
     type="l", xlab="iteration", ylab="y")
plot(selected.model$chains$bgibbsUn[,1]-
mean(selected.model$chains$bgibbsUn[,1]),
     type="l", xlab="iteration", ylab="y")
```

```
plot(selected.model$chains$bgibbs[,1])

# center with 'apply()'
center_apply <- function(x) {
  apply(x, 2, function(y) y - mean(y))
}

# apply it
dfplot <-
data.frame(center_apply(data.frame(selected.model$chains$bgibbs)))
#dfplot <-
data.frame(center_apply(data.frame(selected.model$chains$bgibbs[burning
:ng,])))
#dfplot <-
data.frame(center_apply(data.frame(selected.model$chains$sgibbs)))

plot(dfplot[,5],type="l", xlab="iteration", ylab="y")
dim(dfplot)

#bgibbs
DiagTab <- dfplot %>%
  #  data.frame() %>%
  mutate(Iteration = 1:nrow(dfplot)) %>%
  gather(key = "Parameter", value = "value", -Iteration) %>%
  # filter(Iteration>burning) %>%
  group_by(Parameter) %>%
  summarise(Median = mean(value, na.rm = TRUE), CIlow = quantile(value,
0.025, na.rm = TRUE),
            CIhigh = quantile(value, 0.975, na.rm = TRUE),IQR =
IQR(value)) %>%
  ungroup() %>%
  filter(IQR>quantile(IQR,0.975)) %>% #Q3
  # filter(IQR>10) %>%
  separate(Parameter, into = c("Taxa","Treatment"),sep = "_", remove =
FALSE)

#sgibbs
#DiagTab <- dfplot %>%
#  data.frame() %>%
# mutate(Iteration = 1:nrow(dfplot)) %>%
#gather(key = "Parameter", value = "value", -Iteration) %>%
#filter(Iteration>burning) %>%
#group_by(Parameter) %>%
#summarise(Median = mean(value, na.rm = TRUE), CIlow = quantile(value,
0.025, na.rm = TRUE),
#            CIhigh = quantile(value, 0.975, na.rm = TRUE),IQR =
IQR(value)) %>%
# ungroup() %>%
#filter(IQR>quantile(IQR,0.975))

dim(DiagTab)
View(DiagTab)    #DiagTab gives the samples with the highest divergent
summary(DiagTab)

summary(DiagTab$IQR)

hist(DiagTab$IQR)
```

```
boxplot(DiagTab$IQR)     #important to see in which range the variation happens; if small go on
length(unique(DiagTab$Parameter))
Notconverged <- unique(DiagTab$Parameter)
unique(DiagTab$Taxa) #?

plot(dfplot[,Notconverged[1]]-mean(dfplot[,Notconverged[1]]),type="l", xlab="iteration", ylab="y")
plot(cumsum(dfplot[,Notconverged[1]]),type="l", xlab="iteration", ylab="y")

View(selected.model$inputs$y[,unique(DiagTab$Taxa)])
heatmap(scale(selected.model$inputs$y[,unique(DiagTab$Taxa)]))
apply(selected.model$inputs$y[,unique(DiagTab$Taxa)],2,hist)

library(ggplot2)
#Looking to the chains that did not converged
dfplot %>%
  # mutate_all(list(cumsum)) %>%
  #  data.frame() %>%
  mutate(ng = 1:nrow(dfplot)) %>%
  # filter(ng>burning) %>%
  tidyr::gather(key = VarName, value = MCMC, -ng) %>%
  # filter(VarName %in% sample(Notconverged,20)) %>%
  filter(VarName %in% Notconverged) %>% #If smaller than 20
  ggplot(aes(x = ng, y = MCMC, color = VarName))+
  geom_line()+
  # scale_y_log10()+
  theme(panel.grid.major = element_blank(), panel.grid.minor = element_blank(), panel.background = element_blank(), axis.line = element_line(colour="black"))


#Randomly checking the converged chains
SampleRandom <- sample(colnames(dfplot),20)
dfplot %>%
  #  data.frame() %>%
  mutate(ng = 1:nrow(dfplot)) %>%
  filter(ng>burning) %>%
  tidyr::gather(key = VarName, value = MCMC, -ng) %>%
  #filter(VarName %in% SampleRandom) %>%
  ggplot(aes(x = ng, y = MCMC, color = VarName))+
  geom_line()+
  theme(panel.grid.major = element_blank(), panel.grid.minor = element_blank(), panel.background = element_blank(), axis.line = element_line(colour="black"))
#theme(legend.position = "none")

dfplot %>%
  #  data.frame() %>%
  mutate(ng = 1:nrow(dfplot)) %>%
  filter(ng>burning) %>%
  tidyr::gather(key = VarName, value = MCMC, -ng) %>%
  filter(VarName %in% SampleRandom) %>%
  ggplot(aes(x = VarName, y = MCMC, color = VarName))+
  geom_boxplot()+
  theme(legend.position = "none")
```

```
#if stable save data for next step
save(output_Dilutions,file="gjamOutput_Dilutions.RData")


####ONE WAY ANALYSIS####
#Gjam standard plot

pl  <- list(SAVEPLOTS = TRUE, GRIDPLOTS =TRUE)
gjamPlot( output_Dilutions, plotPars = pl)
selected.model <- output_Dilutions
selected.model$inputs$y
ynames <- colnames(selected.model$inputs$y)
ynames
View(TaxInfo[TaxInfo$GenCode %in% ynames,])

#Coefficients
heatmap(selected.model$parameters$betaStandXWmu)
library("gplots")
netTab <- selected.model$parameters$betaStandXWmu[,1:8]
hT <- heatmap.2(netTab, scale = "column", col = rev(bluered(100)),
cexRow =1, cexCol=1, #lhei=lhei, lwid=lwid,lmat =lmat,
                trace = "none", density.info = "none", labCol =
LabelsCode$AbbTax)


LabelsCode <- data.frame(GenCode = colnames(netTab)) %>%
  left_join(TaxInfo,by = c("GenCode")) %>%
  mutate(AbbTax = paste(Genus)) %>%
  mutate(AbbTax = gsub("NA","un",AbbTax)) %>%
  mutate(AbbTax = ifelse(AbbTax == "un", paste(Family), AbbTax)) %>%
  mutate(AbbTax = ifelse(AbbTax == "NA", paste(Order), AbbTax)) %>%
  mutate(AbbTax = ifelse(AbbTax == "NA", paste(Class), AbbTax)) %>%
  mutate(AbbTax = ifelse(AbbTax == "NA", paste(Phylum), AbbTax)) %>%
  mutate(AbbTax=ifelse(GenCode=="ppmp", paste("soluble P"), AbbTax))


heatmap.2(netTab, scale = "none", col = rev(bluered(100)), cexRow =1,
cexCol=1, #lhei=lhei, lwid=lwid,lmat =lmat,
          trace = "none", density.info = "none", labCol =
LabelsCode$AbbTax)


dendVar <- hT$colDendrogram
labels(dendVar) <- LabelsCode$AbbTax
plot(as.hclust(dendVar), hang = -1, cex = 0.6)



#Null significant effect
coeffTabfinal <- selected.model$parameters$betaStandXWTable
NullSigEff <- coeffTabfinal %>%
  mutate(VarEffect =
rownames(selected.model$parameters$betaStandXWTable)) %>%
  tidyr::separate(VarEffect, into = c("DepVar", "Treat"), sep = "_")
%>%
  #tidyr::separate(DepVar, into = c("Phylum", "Class")) %>%
  # mutate(DepVar = ifelse(DepVar == "HAl", "H_Al", DepVar)) %>% #View
  mutate(DepVar = factor(DepVar, levels = unique(DepVar))) %>%
```

```
  filter(sig95 == "*")

View(NullSigEff)

LabelsCode.sig<-LabelsCode %>%
  filter(GenCode %in% NullSigEff$DepVar)

unique(NullSigEff$DepVar)
heatmap.2(netTab[,unique(NullSigEff$DepVar)], scale = "none", col =
rev(bluered(100)),cexCol = 1, cexRow =1,#lhei=lhei, lwid=lwid,lmat
=lmat,
          trace = "none", density.info = "none", labCol =
LabelsCode.sig$AbbTax)

###Bubble Plot###
library(zCompositions)
selected.model<-output_Dilutions
ydata<-selected.model$inputs$y
d.czm <- cmultRepl(ydata[,1:7],  label=0, method="CZM")
colnames(d.czm)
# The table needs to be transposed again (samples as COLUMNS)
#log - log means division ; relative abudnance
d.clr <- t(apply(d.czm, 1, function(x){log(x) - mean(log(x))}))

ControlData <- cbind(output_Dilutions$inputs$xdata, d.clr) %>% #View
  gather(key = "GenCode", value = "CLR",
contains("Tax"),contains("other")) %>%
  left_join(TaxInfo, by = "GenCode") %>% #View
  filter(Dilution=="D0") %>%
  mutate(AbbTax = paste0(Genus,Species)) %>%
  mutate(AbbTax = gsub("g__","",AbbTax)) %>%
  mutate(AbbTax = gsub("s__"," ",AbbTax)) %>%
  mutate(AbbTax = gsub("NA"," un",AbbTax)) %>%
  mutate(AbbTax = ifelse(AbbTax == " un un", GenCode, AbbTax)) %>%
  dplyr::select(-SampleID) %>% #colnames()
  group_by(Kingdom, Phylum, Class, Order, Family, Genus, Species,
AbbTax,Dilution,  GenCode) %>%
  summarise(CLR.mean = mean(CLR)) %>%
  ungroup() %>%
  mutate(Dilution = gsub("S", "", Dilution)) %>%
  rename(DepVar=GenCode)
View(ControlData)


#Buble plot
#RtN0
#t(EstimateEffRtN0[,-c(1,8)])
CoeffValues <- NullSigEff %>%
  dplyr::select(Estimate,DepVar, Treat) %>%
  mutate(Treatment = gsub("Dilution", "", Treat)) %>%
  filter(Treatment != "D0")

CoeffValues %>%

  mutate(Size = ifelse(Estimate<0, -1*Estimate,Estimate)) %>%
  left_join(ControlData, by = "DepVar") %>% #View

```

```
  mutate(NameG = ifelse(Estimate %in%
tail(sort(CoeffValues$Estimate),5), AbbTax, NA)) %>% #View
  mutate(NameG = ifelse(Estimate %in%
head(sort(CoeffValues$Estimate),5), AbbTax, NameG)) %>% #View
  mutate(Treatment = factor(Treatment, levels = c("D1", "D2"))) %>%
  ggplot(aes(x=CLR.mean, y=Estimate, size=Size,
             color=NameG)) +
  geom_point(alpha=0.5) +
  #geom_text(aes(label=NameG, size = 1),hjust=0.1, vjust=0.1)+
  facet_wrap(~Treatment, scales = "free")+
  geom_hline(yintercept = 0, linetype = "dashed")+
  geom_vline(xintercept = 0, linetype = "dashed")+
  theme_bw()

#####GJAM environmental perturbation#######
####treating of PS###########
#7000taxa if we need all of them so we check

apply(tax_table(ps), 2, unique)
lapply(apply(tax_table(ps), 2, unique), length)
#Kingdom: 4
#Phylum: 37
#Class: 95
#Order: 182
#Family: 234
#Genus: 429
#Species:96

#filter from Kingoms
ps1<-subset_taxa(ps, Kingdom %in% c("Bacteria", "Archaea"))
apply(tax_table(ps1), 2, unique)

#filter further: NA in phylum, "Cyanobacteria" because they are likely
to be chloroplasts
ps2<-subset_taxa(ps1, Phylum!="Cyanobacteria")
ps3<-subset_taxa(ps2, !is.na(Phylum))

apply(tax_table(ps3), 2, unique)
lapply(apply(tax_table(ps3), 2, unique), length)
#Kingdom: 2
#Phylum: 35
#Class: 92
#Order: 178
#Family: 231
#Genus: 426
#Species:94

#different experiment ps data
ps_Fractions <- subset_taxa(ps3, Experiment=="F")
lapply(apply(tax_table(ps_Fractions), 2, unique), length)
#Kingdom: 2
#Phylum: 23
#Class: 57
#Order: 108
#Family: 135
#Genus: 173
#Species:26
```

```
ps_Breeding1 <- subset_taxa(ps3, Experiment=="B1")
lapply(apply(tax_table(ps_Breeding1), 2, unique), length)
#Kingdom: 2
#Phylum: 31
#Class: 76
#Order: 146
#Family: 184
#Genus: 316
#Species:56



pdata<-tax_glom(ps3, taxrank="Genus", NArm=FALSE)
View(data.frame(tax_table(pdata)))

#######GJAM modelling Breeding1######

###PR (as control) vs PB; single factor design####

library(gjam)

xdata  <- data.frame(sample_data(pdata)) %>%
  filter(Experiment == "B1") %>%
  filter(Generation=="G0") %>%
  dplyr::select(-ppmp, -Timepoint)
ppmp  <- c(1.577408118, -0.37270571, 1.783905181, 1.15156508,
           6.326840561, 0.87442494, 2.816390495, -0.23413564,
           2.093650775, 5.30866723, 2.713141963, 1.370911055,
           0.45871472, 1.887153712, 2.196899306, 1.370911055,
           0.18157458, 1.267662524, 1.783905181, 0.73585486,
           1.783905181, -0.09556557, 1.68065665, -0.09556557,
           1.990402244, 0.04300451, 2.196899306, 1.29013515,
           1.783905181, -1.61983636, 1.68065665, 0.32014465,
           2.196899306, 1.84441544, 5.810597904, 0.87442494,
           0.854668399, 0.04300451, 2.196899306, -1.20412614,
           3.745627277, 1.01299501, 1.990402244, 0.32014465)

Timepoint<- c(504, 1368, 648,
              1512, 576, 1440,
              720, 1584, 648,
              1512, 792, 720,
              1584, 864, 792,
              72, 936, 864,
              144, 1008, 72,
              936, 216, 1080,
              144, 1008, 288,
              1152, 216, 1080,
              360, 1224, 288,
              1152, 432, 1296,
              360, 1224, 504,
              1368, 432, 1296,
              576, 1440)

xdata<- cbind(xdata, ppmp)
xdata<-xdata %>%
  mutate(Performance=(gsub("R", "A", Performance)))
```

```
otu    <- data.frame(otu_table(pdata)) %>%
  filter(Experiment == "B1")
TaxInfo<-data.frame(tax_table(pdata)) %>%
  unite("GenInfor", Kingdom:Genus, sep="_", remove=FALSE) %>%
  mutate(GenCode=paste0("Tax", 1:nrow(. )))
colnames(otu)<-TaxInfo$GenCode

otu <- cbind(otu, ppmp)
otu_noT<-otu[!grepl("T", rownames(otu)),]
otu_noR<-otu_noNE[!grepl("T5", rownames(otu_noNE)),]
otu_NE<-otu[grepl("NE", rownames(otu)),]
otu<-otu_noT

otu    <- data.frame(otu_table(pdata)) %>%
  filter(Experiment == "B1")
otu_G0<-otu[!grepl("T", rownames(otu)),]
otu <- otu_G0
View(otu)


hist( as.matrix(otu), ylab = "Reads", main = "OTU's")

min.ob<-ifelse(nrow(otu)*0.05<5,5, round(nrow(otu)*0.05,0))
nobs <- gjamTrimY( otu, minObs = min.ob, OTHER = F )$nobs   #minObs
either 5 or 5% of samples
hist( nobs/ncol(otu), nclass=100, xlab = 'Fraction of observations',
      ylab = 'Frequency', main='Incidence' ) #how common to find in
relation to all number of species



#trim by abundance instead of number of observations
tmp <- gjamTrimY(otu, minObs = min.ob)
y   <- tmp$y

TaxInfo %>%
  filter(!GenCode %in% colnames(y)) %>%
  apply(.,2,unique)

dim(otu)                        # all OTUs; 701
dim(y)                          # trimmed data; 9
tail(colnames(y))               # 'other' class added

#type names to microbial composition and add ppmp to ydata
ppmp  <- as.vector(xdata$ppmp)
ydata <- cbind(y, ppmp)
head(ydata)
S     <- ncol(ydata)
typeNames    <- c(rep('CC',S-1), "CON")   # composition count data and
ppmp as continuous data


xdata$Generation <- factor(xdata$Generation,
levels=unique(xdata$Generation))
xdata$Performance<-factor(xdata$Performance,
levels=unique(xdata$Performance))
```

```
#set level for xdata; Performance
#run model
ml <- list( ng = 15000, burnin = 4000, typeNames = typeNames)
output <- gjam(~ Generation*Performance, xdata, ydata, modelList = ml)

View(output$parameters$betaTable)

#check model fit
fit <- gjamPlot(output, plotPars = list(GRIDPLOTS=T, SAVEPLOTS=TRUE) )

output_Breeding1<-output
fit_Breeding1<-fit

####diagnosis of MCMC chains ####
#to see if model is okay to continue

#Add here your settings for Gibbs sampling#
ng <- 10000
burning <- 2000

#Add here you output from the GJAM analysis
selected.model <- output_Breeding1_Timepoints

plot(cumsum(selected.model$chains$bgibbs[,1]-
mean(selected.model$chains$bgibbs[,1])),
     type="l", xlab="iteration", ylab="y")
#plot(selected.model$chains$bgibbsUn[,1]-
mean(selected.model$chains$bgibbsUn[,1]),
#    type="l", xlab="iteration", ylab="y")

plot(selected.model$chains$bgibbs[,1])

# center with 'apply()'
center_apply <- function(x) {
  apply(x, 2, function(y) y - mean(y))
}

# apply it
dfplot <-
data.frame(center_apply(data.frame(selected.model$chains$bgibbs)))
#dfplot <-
data.frame(center_apply(data.frame(selected.model$chains$bgibbs[burning
:ng,])))
#dfplot <-
data.frame(center_apply(data.frame(selected.model$chains$sgibbs)))

plot(dfplot[,5],type="l", xlab="iteration", ylab="y")
dim(dfplot)

#bgibbs
DiagTab <- dfplot %>%
  #  data.frame() %>%
  mutate(Iteration = 1:nrow(dfplot)) %>%
  gather(key = "Parameter", value = "value", -Iteration) %>%
  # filter(Iteration>burning) %>%
  group_by(Parameter) %>%
  summarise(Median = mean(value, na.rm = TRUE), CIlow = quantile(value,
0.025, na.rm = TRUE),
```

```
            CIhigh = quantile(value, 0.975, na.rm = TRUE),IQR =
IQR(value)) %>%
  ungroup() %>%
  filter(IQR>quantile(IQR,0.975)) %>% #Q3
  # filter(IQR>10) %>%
  separate(Parameter, into = c("Taxa","Treatment"),sep = "_", remove =
FALSE)

#sgibbs
#DiagTab <- dfplot %>%
#  data.frame() %>%
# mutate(Iteration = 1:nrow(dfplot)) %>%
#gather(key = "Parameter", value = "value", -Iteration) %>%
#filter(Iteration>burning) %>%
#group_by(Parameter) %>%
#summarise(Median = mean(value, na.rm = TRUE), CIlow = quantile(value,
0.025, na.rm = TRUE),
#            CIhigh = quantile(value, 0.975, na.rm = TRUE),IQR =
IQR(value)) %>%
# ungroup() %>%
#filter(IQR>quantile(IQR,0.975))

dim(DiagTab)
View(DiagTab)    #DiagTab gives the samples with the highest divergent
summary(DiagTab)

summary(DiagTab$IQR)

hist(DiagTab$IQR)
boxplot(DiagTab$IQR)     #important to see in which range the variation
happens; if small go on
length(unique(DiagTab$Parameter))
Notconverged <- unique(DiagTab$Parameter)
unique(DiagTab$Taxa) #1 not convered ppmp

plot(dfplot[,Notconverged[1]]-mean(dfplot[,Notconverged[1]]),type="l",
xlab="iteration", ylab="y")
plot(cumsum(dfplot[,Notconverged[1]]),type="l", xlab="iteration",
ylab="y")

View(selected.model$inputs$y[,unique(DiagTab$Taxa)])
heatmap(scale(selected.model$inputs$y[,unique(DiagTab$Taxa)]))
apply(selected.model$inputs$y[,unique(DiagTab$Taxa)],2,hist)

library(ggplot2)
#Looking to the chains that did not converged
dfplot %>%
  # mutate_all(list(cumsum)) %>%
  #  data.frame() %>%
  mutate(ng = 1:nrow(dfplot)) %>%
  # filter(ng>burning) %>%
  tidyr::gather(key = VarName, value = MCMC, -ng) %>%
  # filter(VarName %in% sample(Notconverged,20)) %>%
  filter(VarName %in% Notconverged) %>% #If smaller than 20
  ggplot(aes(x = ng, y = MCMC, color = VarName))+
  geom_line()+
  # scale_y_log10()+
```

```
  theme(panel.grid.major = element_blank(), panel.grid.minor =
element_blank(), panel.background = element_blank(), axis.line =
element_line(colour="black"))


#Randomly checking the converged chains
SampleRandom <- colnames(dfplot[,1:14])
dfplot %>%
  #  data.frame() %>%
  mutate(ng = 1:nrow(dfplot)) %>%
  filter(ng>burning) %>%
  tidyr::gather(key = VarName, value = MCMC, -ng) %>%
  filter(VarName %in% SampleRandom) %>%
  ggplot(aes(x = ng, y = MCMC, color = VarName))+
  geom_line()+
  #theme(panel.grid.major = element_blank(), panel.grid.minor =
element_blank(), panel.background = element_blank(), axis.line =
element_line(colour="black"))
  #theme(legend.position = "none")
  theme(panel.grid.major = element_blank(), panel.grid.minor =
element_blank(), panel.background = element_blank(), axis.line =
element_line(colour="black"))


dfplot %>%
  #  data.frame() %>%
  mutate(ng = 1:nrow(dfplot)) %>%
  filter(ng>burning) %>%
  tidyr::gather(key = VarName, value = MCMC, -ng) %>%
  filter(VarName %in% SampleRandom) %>%
  ggplot(aes(x = VarName, y = MCMC, color = VarName))+
  geom_boxplot()+
  theme(legend.position = "none")

#if stable save data for next step
save(output_Breeding1_PB_Cycles,file="gjamOutput_Breeding1_PB_Cycles.RD
ata")


#########Analysis of GJAM output####
#task split up betastandxwtable into two columns (intercept &
treatment) and plot it (dyplr and ggplot)
beta.table<- data.frame(output_Breeding1_EORvsEOB$parameters$betaTable)

#try to minimize code
beta.table %>%
  rownames_to_column(var = "row_name") %>%
  separate(row_name, into = c("Taxa","Treatment"),sep = "_") %>%
  dplyr::select(-c(CI_025,CI_975, SE, sig95)) %>%
  pivot_wider(names_from= "Treatment", values_from=Estimate) %>%
  filter(Taxa!="ppmp") %>%
  ggplot(aes(x=intercept, y=PerformanceB))+
  geom_point()


#filter for significance and link phylogenetic information
Intercept_df<-beta.table %>%
  rownames_to_column(var = "row_name") %>%
```

```
  separate(row_name, into = c("Taxa","Treatment"),sep = "_") %>%
  filter(Treatment=="intercept") %>%
  dplyr::select(-c(CI_025,CI_975, SE, sig95)) %>%
  pivot_wider(names_from= "Treatment", values_from=Estimate)

Taxa_Info<-TaxInfo %>%
  rownames_to_column(var = "row_name") %>%
  #select(c(GenInfor, Phylum, GenCode)) %>% #GenInfor for whole merged
phylogenetic info
  rename(Taxa = GenCode) %>%
  replace_na(list(Genus= "un")) %>%
  replace_na(list(Family= "un")) %>%
  unite(Family, Genus, col="AbbTax", sep=" ")


beta.table %>%
  rownames_to_column(var = "row_name") %>%
  separate(row_name, into = c("Taxa","Treatment"),sep = "_") %>%
  filter(Treatment=="PerformanceB") %>%
  dplyr::select(-c(CI_025,CI_975, SE)) %>%
  filter(sig95=="*") %>%
  pivot_wider(names_from= "Treatment", values_from=Estimate) %>%
  left_join(Intercept_df, by=c("Taxa")) %>%
  left_join(Taxa_Info, by=c("Taxa")) %>%
  dplyr::select(-c(GenInfor)) %>%
  filter(Taxa != "ppmp") %>%
  ggplot(aes(x=intercept, y=PerformanceB, color = Taxa))+
  geom_point(aes(size=PerformanceB))+
  geom_hline(aes(yintercept=0), linetype = "dashed")+
  geom_vline(aes(xintercept = 0), linetype = "dashed")+
  theme(panel.grid.major = element_blank(), panel.grid.minor =
element_blank(), panel.background = element_blank(), axis.line =
element_line(colour="black"))+
  labs(x="intercept", y="best performing", title="Best performing
significant offspring genera", fill="Phylum Genus")



####ONE WAY ANALYSIS####
#Gjam standard plot

pl  <- list(SAVEPLOTS = TRUE, GRIDPLOTS =TRUE)
gjamPlot( output_Breeding1_Timepoints, plotPars = pl)
selected.model <- output_Breeding1_PRvsPB
selected.model$inputs$y
ynames <- colnames(selected.model$inputs$y)
ynames
View(TaxInfo[TaxInfo$GenCode %in% ynames,])

#Coefficients
heatmap(selected.model$parameters$betaStandXWmu)
library("gplots")
netTab <- selected.model$parameters$betaStandXWmu
hT <- heatmap.2(netTab, scale = "column", Rowv= "none", col =
rev(bluered(100)), cexRow =1, cexCol=1, #lhei=lhei, lwid=lwid,lmat
=lmat,
                 trace = "none", density.info = "none" , labCol =
LabelsCode$AbbTax)
```

```
LabelsCode <- data.frame(GenCode = colnames(netTab)) %>%
  left_join(TaxInfo,by = c("GenCode")) %>%
  mutate(AbbTax = paste(Genus)) %>%
  mutate(AbbTax = gsub("NA","un",AbbTax)) %>%
  mutate(AbbTax = ifelse(AbbTax == "un", paste(Family), AbbTax)) %>%
  mutate(AbbTax = ifelse(AbbTax == "NA", paste(Order), AbbTax)) %>%
  mutate(AbbTax = ifelse(AbbTax == "NA", paste(Class), AbbTax)) %>%
  mutate(AbbTax = ifelse(AbbTax == "NA", paste(Phylum), AbbTax)) %>%
  mutate(AbbTax=ifelse(GenCode=="ppmp", paste("soluble P"), AbbTax))


dendVar <- hT$colDendrogram
labels(dendVar) <- LabelsCode$AbbTax
plot(as.hclust(dendVar), hang = -1, cex = 0.6)



#Null significant effect
coeffTabfinal <- selected.model$parameters$betaStandXWTable
NullSigEff <- coeffTabfinal %>%
  mutate(VarEffect =
rownames(selected.model$parameters$betaStandXWTable)) %>%
  tidyr::separate(VarEffect, into = c("DepVar", "Treat"), sep = "_")
%>%
  #tidyr::separate(DepVar, into = c("Phylum", "Class")) %>%
  # mutate(DepVar = ifelse(DepVar == "HAl", "H_Al", DepVar)) %>% #View
  mutate(DepVar = factor(DepVar, levels = unique(DepVar))) %>%
  filter(sig95 == "*")

View(NullSigEff)

LabelsCode.sig<-LabelsCode %>%
  filter(GenCode %in% NullSigEff$DepVar)

unique(NullSigEff$DepVar)
heatmap.2(netTab[,unique(NullSigEff$DepVar)], scale = "none", col =
rev(bluered(100)),cexCol = 1, cexRow =1,#lhei=lhei, lwid=lwid,lmat
=lmat,
          trace = "none", density.info = "none", labCol =
LabelsCode.sig$AbbTax)

###Bubble Plot###
library(zCompositions)
selected.model<-output_Breeding1_PRvsPB
ydata<-selected.model$inputs$y
d.czm <- cmultRepl(ydata[,1:6],  label=0, method="CZM")
colnames(d.czm)
# The table needs to be transposed again (samples as COLUMNS)
#log - log means division ; relative abudnance
d.clr <- t(apply(d.czm, 1, function(x){log(x) - mean(log(x))}))

ControlData <- cbind(selected.model$inputs$xdata, d.clr) %>% #View
  gather(key = "GenCode", value = "CLR",
contains("Tax"),contains("other")) %>%
  left_join(TaxInfo, by = "GenCode") %>% #View
  filter(Performance == "A") %>%
```

```
  mutate(AbbTax = paste0(Genus)) %>%
  mutate(AbbTax = gsub("NA","un",AbbTax)) %>%
  mutate(AbbTax = ifelse(AbbTax == "un", paste(Family), AbbTax)) %>%
  mutate(AbbTax = ifelse(AbbTax == "NA", paste(Order), AbbTax)) %>%
  mutate(AbbTax = ifelse(AbbTax == "NA", paste(Class), AbbTax)) %>%
  mutate(AbbTax = ifelse(AbbTax == "NA", paste(Phylum), AbbTax)) %>%
  dplyr::select(-SampleID) %>% #colnames()
  group_by(Kingdom, Phylum, Class, Order, Family, Genus, Species,
AbbTax,Performance,  GenCode) %>%
  summarise(CLR.mean = mean(CLR)) %>%
  ungroup() %>%
  mutate(Performance = gsub("S", "", Performance)) %>%
  dplyr::rename(DepVar=GenCode)
View(ControlData)


CoeffValues <- NullSigEff %>%
  dplyr::select(Estimate,DepVar, Treat) %>%
  mutate(Performance = gsub("Performance", "", Treat)) %>%
  filter(Treat != "PerformanceA")

CoeffValues %>%

  mutate(Size = ifelse(Estimate<0, -1*Estimate,Estimate)) %>%
  left_join(ControlData, by = "DepVar") %>% #View

  mutate(NameG = ifelse(Estimate %in%
tail(sort(CoeffValues$Estimate),5), AbbTax, NA)) %>% #View
  mutate(NameG = ifelse(Estimate %in%
head(sort(CoeffValues$Estimate),5), AbbTax, NameG)) %>% #View
  mutate(Performance.x = factor(Performance.x, levels = c("G1"))) %>%
  ggplot(aes(x=CLR.mean, y=Estimate, size=Size,
             color=NameG)) +
  geom_point(alpha=0.5) +
  geom_text(aes(label=NameG, size = 2),vjust=0.1) +#, vjust=0.1)+
  #facet_wrap(~Performance.x, scales = "free")+
  geom_hline(yintercept = 0, linetype = "dashed")+
  geom_vline(xintercept = 0, linetype = "dashed")+
  theme_bw()

####Factorial GJAM####
#no control
library(gjam)
selected.model <- output_Breeding1
dim(selected.model$chains$bgibbs)
ExName <- colnames(selected.model$parameters$betaMu)[1]
# paste0(TaxaSelected,Fact1Name,unique(fact1))
library(dplyr)
ng <- 15000
fact1 <- "Generation"
fact2 <- "Performance"
df.gibbs3 <- GJAM.Factorial(selected.model,F1Name = fact1,F2Name =
fact2,
                               Taxa=ExName, ng=ng,burning=ng-100)
colnames(df.gibbs3)

df.gibbs3 %>%
  gather(key = "VarName", value = "Coeff") %>%
```

```
  separate(VarName, into = c("Fact1", "Fact2"), sep = "_", extra =
"merge") %>%
  ggplot(aes(x = Fact1, y = Coeff, fill = Fact2)) +
  geom_boxplot()+
  geom_hline(yintercept = 0, linetype = "dashed")+
  # scale_x_discrete(limits =fact1)+
  theme_bw()

df.gibbs3 %>%
  gather(key = "VarName", value = "Coeff") %>%
  separate(VarName, into = c("Fact1", "Fact2"), sep = "_", extra =
"merge") %>%
  ggplot(aes(x = Fact1, y = Coeff, fill = Fact2)) +
  geom_boxplot(outlier.size = 0) +
  stat_summary(fun="mean", geom="point", shape=23, size=4,
fill="white") +
  #guides(fill=FALSE)+
  geom_hline(yintercept = 0, linetype = "dashed")+
  # scale_x_discrete(limits =fact1)+
  theme_bw()

quantiles_95 <- function(x) {
  r <- quantile(x, probs=c(0.025, 0.25, 0.5, 0.75, 0.975))
  names(r) <- c("ymin", "lower", "middle", "upper", "ymax")
  r
}

lvF1 <- levels(as.data.frame(selected.model$inputs$xdata)[,fact1])
lvF2 <- levels(as.data.frame(selected.model$inputs$xdata)[,fact2])

df.gibbs3 %>%
  gather(key = "VarName", value = "Coeff") %>%
  separate(VarName, into = c("Fact1", "Fact2"), sep = "_", extra =
"merge") %>%
  mutate(Fact1 = factor(Fact1, levels = lvF1)) %>%
  mutate(Fact2 = factor(Fact2, levels = lvF2)) %>%
  ggplot(aes(x = Fact2, y = Coeff, fill = Fact1)) +
  # guides(fill=F) +
  stat_summary(fun.data = quantiles_95, geom="boxplot",position =
"dodge")+
  stat_summary(fun="median", geom="point", shape=23, size=4,
fill="white") +
  stat_smooth(method="lm", formula=y~1, se=FALSE, aes(group = 1)) +
  # stat_summary(fun.y = "mean", color = "red", geom = "line",
aes(group = 1))+
  # geom_hline(yintercept = 0, linetype = "dashed")+
  # scale_x_discrete(limits =fact1)+
  theme_bw()

df.gibbs3 %>%
  gather(key = "VarName", value = "Coeff") %>%
  separate(VarName, into = c("Fact1", "Fact2"), sep = "_", extra =
"merge") %>%
  mutate(Fact1 = factor(Fact1, levels = lvF1)) %>%
  mutate(Fact2 = factor(Fact2, levels = lvF2)) %>%
  ggplot(aes(x = Fact1, y = Coeff, fill = Fact2)) +
  # guides(fill=F) +
```

```
  stat_summary(fun.data = quantiles_95, geom="boxplot",position =
"dodge")+
  stat_summary(fun="median", geom="point", shape=23, size=4,
fill="white") +
  stat_smooth(method="lm", formula=y~1, se=FALSE, aes(group = 1)) +
  # stat_summary(fun.y = "mean", color = "red", geom = "line",
aes(group = 1))+
  # geom_hline(yintercept = 0, linetype = "dashed")+
  # scale_x_discrete(limits =fact1)+
  theme_bw()

barplot(apply(df.gibbs3,2,mean))
barplot(output$parameters$betaMu[,1])

multinames <- function(varnames){
  print(varnames)
  res <- GJAM.Factorial(output = selected.model,
                        F1Name = fact1,F2Name = fact2,
                        Taxa=varnames, ng=ng,burning=ng-100)
  return(res)
}

Fact.Res <-
lapply(colnames(selected.model$parameters$betaMu),multinames)
names(Fact.Res) <- colnames(selected.model$parameters$betaMu)
titleName <- names(Fact.Res[2])
Fact.Res[[2]]  %>%
  gather(key = "VarName", value = "Coeff") %>%
  separate(VarName, into = c("Fact1", "Fact2"), sep = "_", extra =
"merge") %>%
  mutate(Fact1 = factor(Fact1, levels = lvF1)) %>%
  mutate(Fact2 = factor(Fact2, levels = lvF2)) %>%
  ggplot(aes(x = Fact2, y = Coeff, fill = Fact1)) +
  # guides(fill=F) +
  stat_summary(fun.data = quantiles_95, geom="boxplot",position =
"dodge")+
  stat_summary(fun="median", geom="point", shape=23, size=4,
fill="white") +
  stat_smooth(method="lm", formula=y~1, se=FALSE, aes(group = 1)) +
  # stat_summary(fun.y = "mean", color = "red", geom = "line",
aes(group = 1))+
  geom_hline(yintercept = 0, linetype = "dashed")+
  # ggtitle(titleName)+
  # scale_x_discrete(limits =fact1)+
  theme_bw()

Fact.Res[[2]]  %>%
  gather(key = "VarName", value = "Coeff") %>%
  separate(VarName, into = c("Fact1", "Fact2"), sep = "_", extra =
"merge") %>%
  mutate(Fact1 = factor(Fact1, levels = lvF1)) %>%
  mutate(Fact2 = factor(Fact2, levels = lvF2)) %>%
  ggplot(aes(x = Fact2, y = Coeff, fill = Fact1)) +
  stat_summary(fun.data = quantiles_95, geom="boxplot",position =
"dodge")+
  stat_summary(fun="median", geom="point", shape=23, size=4,
fill="white") +
  stat_smooth(method="lm", formula=y~1, se=FALSE, aes(group = 1)) +
```

```
  # stat_summary(fun.y = "mean", color = "red", geom = "line",
aes(group = 1))+
  geom_hline(yintercept = 0, linetype = "dashed")+
  # scale_x_discrete(limits =fact1)+
  theme_bw()

#All Variables
do.call(rbind,Fact.Res[1:2])
Fact.Res.DF <- data.frame(TaxVar =
rep(names(Fact.Res),each=nrow(data.frame(Fact.Res[1]))),
                          do.call(rbind,Fact.Res))
View(Fact.Res.DF)

#select specific taxas
TaxVarS <- unique(Fact.Res.DF$TaxVar)
Fact.Res.DF  %>%
  gather(key = "VarName", value = "Coeff", -TaxVar) %>%
  filter(TaxVar %in% TaxVarS[1:9]) %>% #analisa de 1 a 9
  separate(VarName, into = c("Fact1", "Fact2"), sep = "_", extra =
"merge") %>%
  mutate(Fact1 = factor(Fact1, levels = lvF1)) %>%
  mutate(Fact2 = factor(Fact2, levels = lvF2)) %>%
  ggplot(aes(x = Fact1, y = Coeff, fill = Fact2)) +
  #guides(fill="none") +
  stat_summary(fun.data = quantiles_95, geom="boxplot",position =
"dodge")+
  stat_summary(fun="median", geom="point", shape=23, size=4,
fill="white") +
  stat_smooth(method="lm", formula=y~1, se=FALSE, aes(group = 1)) +
  # stat_summary(fun.y = "mean", color = "red", geom = "line",
aes(group = 1))+
  geom_hline(yintercept = 0, linetype = "dashed")+
  # scale_x_discrete(limits =fact1)+
  facet_wrap(~TaxVar)+
  theme_bw()

Fact.Res.DF[,paste0(lvF1[1],"_",lvF2[1])]

tH <- Fact.Res.DF  %>% # estimate the coefficients
  gather(key = "VarName", value = "Coeff", -TaxVar) %>%
  separate(VarName, into = c("Fact1", "Fact2"), sep = "_", extra =
"merge", remove = FALSE) %>% #View
  group_by(TaxVar,Fact1,Fact2,VarName) %>%
  summarise(Mean = mean(Coeff),Q1 = quantile(Coeff, 0.025),
            Q3 = quantile(Coeff, 0.975)) %>%
  ungroup()

tH0 <- tH %>%
  group_by(TaxVar) %>%
  summarise(H0 = mean(Mean)) %>%
  ungroup()

tHF1 <- tH %>%
  group_by(TaxVar,Fact1) %>%
  summarise(H0F1 = mean(Mean)) %>%
  ungroup()

tHF2 <- tH %>%
```

```
  group_by(TaxVar,Fact2) %>%
  summarise(H0F2 = mean(Mean)) %>%
  ungroup()

tHcF1 <- Fact.Res.DF  %>% # estimate the coefficients
  gather(key = "VarName", value = "Coeff", -TaxVar) %>%
  separate(VarName, into = c("Fact1", "Fact2"), sep = "_", extra =
"merge", remove = FALSE) %>% #View
  group_by(TaxVar,Fact1,Fact2,VarName) %>%
  summarise(Mean = mean(Coeff),Q1 = quantile(Coeff, 0.025),
            Q3 = quantile(Coeff, 0.975)) %>%
  ungroup() %>%
  filter(Fact1 == lvF1[1]) %>%
  dplyr::select(TaxVar,Fact2,Mean) %>%
  rename(HcF1 = Mean)

tHcF2 <- Fact.Res.DF  %>% # estimate the coefficients
  gather(key = "VarName", value = "Coeff", -TaxVar) %>%
  separate(VarName, into = c("Fact1", "Fact2"), sep = "_", extra =
"merge", remove = FALSE) %>% #View
  group_by(TaxVar,Fact1,Fact2,VarName) %>%
  summarise(Mean = mean(Coeff),Q1 = quantile(Coeff, 0.025),
            Q3 = quantile(Coeff, 0.975)) %>%
  ungroup() %>%
  filter(Fact2 == lvF2[1]) %>%
  dplyr::select(TaxVar,Fact1,Mean) %>%
  rename(HcF2 = Mean)

tHF <- tH %>%
  left_join(tH0, by = "TaxVar") %>%
  left_join(tHF1, by = c("TaxVar","Fact1")) %>%
  left_join(tHF2, by = c("TaxVar","Fact2")) %>%
  left_join(tHcF1, by = c("TaxVar","Fact2")) %>%
  left_join(tHcF2, by = c("TaxVar","Fact1")) %>%
  mutate(SignH0 = ifelse(Q1<=H0 & Q3>=H0, "", "*")) %>%  #If any
difference between treatments
  mutate(SignH0F1 = ifelse(Q1<=H0F1 & Q3>=H0F1, "", "*")) %>% #if any
difference between Fact1 treatments
  mutate(SignH0F2 = ifelse(Q1<=H0F2 & Q3>=H0F2, "", "*")) %>%  #if any
difference between Fact2 treatments
  mutate(SignHcF1 = ifelse(Q1<=HcF1 & Q3>=HcF1, "", "*")) %>%  #if
treatments in Fact1 differs from the control within Fact2
  mutate(SignHcF2 = ifelse(Q1<=HcF2 & Q3>=HcF2, "", "*")) #if
treatments in Fact2 differs from the control within Fact1

tHF_Breeding1<-tHF

SigVarsH0 <- as.vector(unique(tHF[tHF$SignH0=="*","TaxVar"]))
SigVarsH0F1 <- as.vector(unique(tHF[tHF$SignH0F1=="*","TaxVar"]))
SigVarsH0F2 <- as.vector(unique(tHF[tHF$SignH0F2=="*","TaxVar"]))
SigVarsHcF1 <- as.vector(unique(tHF[tHF$SignHcF1=="*","TaxVar"]))
SigVarsHcF2 <- as.vector(unique(tHF[tHF$SignHcF2=="*","TaxVar"]))

#Take the first 9 significant ones
SignTax <- as.vector(SigVarsH0$TaxVar)
SignTax <- as.vector(SigVarsH0F1$TaxVar)
SignTax <- as.vector(SigVarsH0F2$TaxVar)
SignTax <- as.vector(SigVarsHcF1$TaxVar)
```

```
SignTax <- as.vector(SigVarsHcF2$TaxVar)
SignTax


SignSign <- tHF %>%
  mutate(Fact1 = factor(Fact1, levels = lvF1)) %>%
  mutate(Fact2 = factor(Fact2, levels = lvF2)) %>% #View
  #filter(TaxVar %in% SignTax) %>%
  filter(SignHcF1=="*") %>%
  mutate(SignPosition = ifelse(SignHcF2 == "*", Q3,NA))
summary(filteredSign)

Fact.Res.DF  %>%
  gather(key = "VarName", value = "Coeff", -TaxVar) %>%
  #filter(TaxVar %in% filteredTaxa) %>%
  separate(VarName, into = c("Fact1", "Fact2"), sep = "_", extra =
"merge") %>%
  left_join(filteredSign,by=c("TaxVar", "Fact1", "Fact2")) %>%
  mutate(Fact1 = factor(Fact1, levels = lvF1)) %>%
  mutate(Fact2 = factor(Fact2, levels = lvF2)) %>% #View
  ggplot(aes(x = Fact1, y = Coeff, fill = Fact2)) +
  #guides(fill="none") +
  stat_summary(fun.data = quantiles_95, geom="boxplot",position =
"dodge")+
  stat_summary(fun="mean", geom="point", shape=23, size=4,
fill="white") +
  stat_smooth(method="lm", formula=y~1, se=FALSE, aes(group = 1)) +
  # stat_summary(fun.y = "mean", color = "red", geom = "line",
aes(group = 1))+
  geom_hline(yintercept = 0, linetype = "dashed")+
  # scale_x_discrete(limits =fact1)+

#geom_text(data=filteredSign,aes(x=Fact1,group=Fact2,y=SignPosition,lab
el=SignHcF2),
    #        size=5, position = position_dodge(width=0.9))+
  # geom_text(aes(label = SignHcF1,x=Fact1, y=Q3),label="*", position =
"fill")+
  facet_wrap(~TaxVar,scales = "free_y")+
  theme_bw()

#Significant Effects within Factor 2
filteredTaxa <- SignTax[5:13]
filteredSign <- tHF %>%
  mutate(Fact1 = factor(Fact1, levels = lvF1)) %>%
  mutate(Fact2 = factor(Fact2, levels = lvF2)) %>% #View
  #filter(TaxVar %in% filteredTaxa) %>%
  mutate(SignPosition = ifelse(SignHcF1 == "*", Q3,NA))

Fact.Res.DF  %>%
  gather(key = "VarName", value = "Coeff", -TaxVar) %>%
  filter(TaxVar %in% filteredTaxa) %>%
  separate(VarName, into = c("Fact1", "Fact2"), sep = "_", extra =
"merge") %>%
  left_join(filteredSign,by=c("TaxVar", "Fact1", "Fact2")) %>%
  mutate(Fact1 = factor(Fact1, levels = lvF1)) %>%
  mutate(Fact2 = factor(Fact2, levels = lvF2)) %>% #View
  ggplot(aes(x = Fact2, y = Coeff, fill = Fact1)) +
  #guides(fill="none") +
```

```
  stat_summary(fun.data = quantiles_95, geom="boxplot",position =
"dodge")+
  stat_summary(fun="mean", geom="point", shape=23, size=4,
fill="white") +
  stat_smooth(method="lm", formula=y~1, se=FALSE, aes(group = 1)) +
  # stat_summary(fun.y = "mean", color = "red", geom = "line",
aes(group = 1))+
  geom_hline(yintercept = 0, linetype = "dashed")+
  # scale_x_discrete(limits =fact1)+

geom_text(data=filteredSign,aes(x=Fact2,group=Fact1,y=SignPosition,labe
l=SignHcF1),
            size=5, position = position_dodge(width=1))+
  #geom_text(aes(label = SignHcF1,x=Fact1, y=Q3),label="*", position =
"fill")+
  facet_wrap(~TaxVar,scales = "free_y")+
  theme_bw()

TaxCode <- TaxInfo %>%
  rename(TaxVar=GenCode)
Fact.Res.DF  %>%
  filter(TaxVar %in% unique(filteredSign$TaxVar)) %>% #dim()
  gather(key = "VarName", value = "Coeff", -TaxVar) %>% #View()
  group_by(TaxVar,VarName) %>%
  summarise(median = quantiles_95(Coeff)[3],
            ci_lower=quantiles_95(Coeff)[1],
            ci_upper=quantiles_95(Coeff)[5]) %>% #View
  separate(VarName, into = c("Fact1", "Fact2"), sep = "_", extra =
"merge") %>%
  # separate(TaxVar, into = c("Kingdom", "Phylum", "Class", "Order",
"Family", "Genus"), remove = FALSE,
  #          sep = "\\.", fill = "left", extra = "merge") %>%
  # mutate(AllVar = ifelse(Genus == "g",paste0(Family,Genus),Genus))
%>%
  # mutate(AllVar = ifelse(AllVar == "fg", paste0(Order,AllVar),
AllVar)) %>% #View
  mutate(Fact1 = factor(Fact1, levels = lvF1)) %>%
  mutate(Fact2 = factor(Fact2, levels = lvF2)) %>%
  ggplot(aes(x = TaxVar, y = median, fill = Fact2)) +
  #guides(fill=F) +
  # geom_boxplot() +
  #stat_summary(fun.data = quantiles_95, geom="boxplot",position =
"dodge")+
  geom_hline(yintercept = 0, linetype = "dashed")+
  geom_linerange(aes(ymin=ci_lower, ymax=ci_upper), position =
position_dodge(width = 0.75))+
  geom_point(size=1, position = position_dodge(width = 0.75))+
  # scale_x_discrete(limits =fact1)+

#geom_text(data=filteredSign,aes(x=TaxVar,group=Fact2,y=SignPosition,la
bel=SignHcF2),
  #         size=5, position = position_dodge(width=0.9))+
  facet_wrap(~Fact1, ncol = 2, scales = "free_x")+
  coord_flip()+
  theme_bw()

#regression coefficients with taxinfo
#all sig Taxa within factor 1
```

```
filteredSign <- tHF %>%
  mutate(Fact1 = factor(Fact1, levels = lvF1)) %>%
  mutate(Fact2 = factor(Fact2, levels = lvF2)) %>% #View
  filter(SignH0F1=="*")

filteredSignT <- tHF %>%
  mutate(SignHcF2 = ifelse(SignHcF2 == "*", "#", SignHcF2)) %>%
  mutate(SignHcF1F2 = paste0(SignHcF1,SignHcF2)) %>%
  filter(SignHcF1F2!="")

TaxCode <- TaxInfo %>%
  rename(TaxVar=GenCode) %>%
  #filter(TaxVar %in% unique(filteredSign$TaxVar)) %>%
  mutate(AbbTax = paste(Genus)) %>%
  mutate(AbbTax = gsub("NA","un",AbbTax)) %>%
  mutate(AbbTax = ifelse(AbbTax == "un", paste(Family), AbbTax)) %>%
  mutate(AbbTax = ifelse(AbbTax == "NA", paste(Order), AbbTax)) %>%
  mutate(AbbTax = ifelse(AbbTax == "NA", paste(Class), AbbTax)) %>%
  mutate(AbbTax = ifelse(AbbTax == "NA", paste(Phylum), AbbTax))

Fact.Res.DF  %>%
  filter(TaxVar %in% unique(filteredSign$TaxVar)) %>% #dim()
  gather(key = "VarName", value = "Coeff", -TaxVar) %>% #View()
  filter(TaxVar!="ppmp") %>%
  group_by(TaxVar,VarName) %>%
  summarise(median = quantiles_95(Coeff)[3],
            ci_lower=quantiles_95(Coeff)[1],
            ci_upper=quantiles_95(Coeff)[5]) %>%
  mutate(Sig = ifelse(ci_lower<=0 & ci_upper>=0, "", "*")) %>%
  filter(Sig=="*") %>%
  left_join(TaxCode, by = "TaxVar") %>%
  filter(AbbTax!="NA") %>%
  ggplot(aes(x = AbbTax, y = median)) +
  geom_hline(yintercept = 0, linetype = "dashed")+
  geom_linerange(aes(ymin=ci_lower, ymax=ci_upper, color = ci_upper<0),
position = position_dodge(width = 0.75))+
  geom_point(aes(color = median<0), size=1, position =
position_dodge(width = 0.75))+
  facet_wrap(~VarName, ncol = 4, scales = "free_x")+
  guides(color="none") +
  coord_flip()+
  labs(y="Regression coefficients", x= NULL)+
  theme(panel.grid.major = element_blank(), panel.grid.minor =
element_blank(),
        panel.background = element_blank(), axis.line =
element_line(colour="black"),
        axis.text = element_text(size=8))

##factorial gjam heatmaps
#heatmap for factor 1
heatmapTab <- tHF %>%
  dplyr::select(TaxVar,VarName,Mean) %>%
  spread(key = TaxVar,value = Mean, fill = 0) %>%
  dplyr::select(-other)
heatmapTabSign <- tHF %>%
  dplyr::select(TaxVar,VarName,SignHcF1) %>%
  spread(key = TaxVar,value = SignHcF1, fill = NA) %>%
  dplyr::select(-other)
```

```
heatmapTabSignT <- tHF %>%
  mutate(SignHcF2 = ifelse(SignHcF2 == "*", "#", SignHcF2)) %>%
  mutate(SignHcF1F2 = paste0(SignHcF1,SignHcF2)) %>%
  dplyr::select(TaxVar,VarName,SignHcF1F2) %>%
  spread(key = TaxVar,value = SignHcF1F2, fill = NA) %>%
  dplyr::select(-other)

LabelsCode <- data.frame(TaxVar = colnames(heatmapTab)) %>%
  left_join(TaxCode,by = c("TaxVar")) %>%
  filter(TaxVar!="VarName") %>%
  mutate(AbbTax = paste(Genus)) %>%
  mutate(AbbTax = gsub("NA","un",AbbTax)) %>%
  mutate(AbbTax = ifelse(AbbTax == "un", paste(Family), AbbTax)) %>%
  mutate(AbbTax = ifelse(AbbTax == "NA", paste(Order), AbbTax)) %>%
  mutate(AbbTax = ifelse(AbbTax == "NA", paste(Class), AbbTax)) %>%
  mutate(AbbTax = ifelse(AbbTax == "NA", paste(Phylum), AbbTax)) %>%
  mutate(AbbTax = ifelse(AbbTax == "NA", paste("ppmp"), AbbTax))

colnames(heatmapTab)
heatmap.2(as.matrix(heatmapTab[,-1]), scale = "column", col =
rev(bluered(100)), #lhei=lhei, lwid=lwid,lmat =lmat,
          trace = "none", density.info = "none", labRow =
heatmapTab$VarName,
          cellnote = as.matrix(heatmapTabSignT[,-1]),notecol = "black",
          labCol = LabelsCode$AbbTax)

#heatmap showing only significant taxa
matrixRes <- as.matrix(heatmapTab[,-1])
matrixSign <- as.matrix(heatmapTabSign[,-1])

matrixRes[matrixSign=="*"] <- 0
matrixRes <- matrixRes[,colSums(matrixRes)!=0]

heatmap.2(matrixRes[,unique(SigVarsHcF2$TaxVar)], scale = "column", col
= rev(bluered(100)), #lhei=lhei, lwid=lwid,lmat =lmat,
          trace = "none", density.info = "none", labRow =
heatmapTab$VarName,
          notecol = "black")

#bubble plot
#Converting the data to CLR; real 0 or due to seq
selected.model<-output_Breeding1
library(zCompositions)
d.czm <- cmultRepl(selected.model$inputs$y[,1:8],  label=0,
method="CZM")
colnames(d.czm)
# The table needs to be transposed again (samples as COLUMNS)
#log - log means division ; relative abudnance
d.clr <- t(apply(d.czm, 1, function(x){log(x) - mean(log(x))}))

ControlData <- cbind(selected.model$inputs$xdata, d.clr) %>% #View
  gather(key = "TaxVar", value = "CLR",
contains("Tax"),contains("other")) %>%
  left_join(TaxCode, by = "TaxVar") %>% #View
  filter(Generation != "G1") %>%
  filter(Performance!= "B") %>%
  mutate(AbbTax = paste(Genus)) %>%
```

```
  mutate(AbbTax = gsub("NA","un",AbbTax)) %>%
  mutate(AbbTax = ifelse(AbbTax == "un", paste(Family), AbbTax)) %>%
  mutate(AbbTax = ifelse(AbbTax == "NA", paste(Order), AbbTax)) %>%
  mutate(AbbTax = ifelse(AbbTax == "NA", paste(Class), AbbTax)) %>%
  dplyr::select(-SampleID) %>% #colnames()
  group_by(Kingdom, Phylum, Class, Order, Family, Genus, Species,
AbbTax, Generation,  TaxVar) %>%
  summarise(CLR.mean = mean(CLR)) %>%
  ungroup()
View(ControlData)

CoeffValues <- tHF %>%
  mutate(SignHcF2 = ifelse(SignHcF2 == "*", "#", SignHcF2)) %>%
  mutate(SignHcF1F2 = paste0(SignHcF1,SignHcF2)) %>%
  filter(SignHcF1F2!="") %>%
  filter(TaxVar!="ppmp") %>%
  filter(TaxVar!="other") %>%
  dplyr::select(TaxVar,VarName, Mean, SignHcF1F2)

CoeffValues %>%
  mutate(Size = ifelse(Mean<0, -1*Mean,Mean)) %>%
  left_join(ControlData, by = "TaxVar") %>% #View
  mutate(NameG = ifelse(Mean %in% tail(sort(CoeffValues$Mean),5),
AbbTax, NA)) %>% #View
  mutate(NameG = ifelse(Mean %in% head(sort(CoeffValues$Mean),5),
AbbTax, NameG)) %>% #View
  mutate(VarName = factor(VarName, levels = c("G0_A", "G0_B", "G1_A",
"G1_B"))) %>%
  #filter(VarName=="G0_B") %>%
  filter(Family!="NA") %>%
  ggplot(aes(x=CLR.mean, y=Mean, size=Size,
             color=AbbTax)) +
  geom_point(alpha=0.5) +
  geom_text(aes(label=AbbTax),hjust=0.1, vjust=0.1, check_overlap =
TRUE)+
  facet_wrap(~VarName, scales = "free")+
  geom_hline(yintercept = 0, linetype = "dashed")+
  geom_vline(xintercept = 0, linetype = "dashed")+
  theme_bw()

###save taxa for soil experiment###
Tax_Breeding1_R<- tHF %>%
  filter(Fact2=="A") %>%
  filter(TaxVar!="ppmp", TaxVar!="other")
Tax_Breeding1_B<-tHF %>%
  filter(Fact2=="B") %>%
  filter(TaxVar!="ppmp", TaxVar!="other")

Tax_Breeding1<-rbind(Tax_Breeding1_R, Tax_Breeding1_B)


####LOOK AT SEQUENCES PER TIMEPOINT####
selected.model<-output_Breeding1$inputs$y
y<-output_Breeding1$inputs$y[,1:8]
ppmp_df<-as.data.frame(output_Breeding1$inputs$y)
ppmp<-ppmp_df %>%
  dplyr::select(ppmp) %>%
  rownames_to_column(var = "VarName")
```

```
df_seqno<-as.data.frame(output_Breeding1$inputs$y[,1:8]) %>%
  rownames_to_column(var = "VarName") %>%
  separate(VarName, into = c("Community","Generation"),sep = "R3G0")
%>%
  filter(Generation!="NA") %>%
  filter(Community=="NE") %>%
  mutate(Generation = factor(Generation, levels = c("C1", "C2", "C3",
"C4", "C5",
                                                     "C6", "C7", "C8",
"C9", "C10",
                                                     "C11", "C12"))) %>%
  arrange(by_group=Generation)


d.czm <- cmultRepl(y,  label=0, method="CZM")
d.clr <- t(apply(d.czm, 1, function(x){log(x) - mean(log(x))}))
d.clr_Breeding1<-d.clr

TaxCode <- TaxInfo %>%
  dplyr::rename(Taxa=GenCode) %>%
  #filter(TaxVar %in% unique(filteredSign$TaxVar)) %>%
  mutate(AbbTax = paste(Genus)) %>%
  mutate(AbbTax = gsub("NA","un",AbbTax)) %>%
  mutate(AbbTax = ifelse(AbbTax == "un", paste(Family), AbbTax)) %>%
  mutate(AbbTax = ifelse(AbbTax == "NA", paste(Order), AbbTax)) %>%
  mutate(AbbTax = ifelse(AbbTax == "NA", paste(Class), AbbTax)) %>%
  mutate(AbbTax = ifelse(AbbTax == "NA", paste(Phylum), AbbTax))


z_score<-cbind(xdata, ppmp) %>%
  dplyr::select(ppmp) %>%
  rownames_to_column(var = "VarName")

fact<-max(EE_clr$ppmp)/max(EE_clr$CLR)


EE_clr<-as.data.frame(d.clr) %>%
  rownames_to_column(var = "VarName") %>%
  separate(VarName, into = c("Community","Generation"),sep = "R3G0")
%>%
  filter(Community=="EE") %>%
  mutate(Generation = factor(Generation, levels = c("C1", "C2", "C3",
"C4", "C5",
                                                     "C6", "C7", "C8",
"C9", "C10",
                                                     "C11", "C12"))) %>%
  arrange(by_group=Generation) %>%
  pivot_longer(cols=3:8, names_to = "Taxa", values_to = "CLR") %>%
  left_join(TaxCode, by = "Taxa") %>%
  ggplot(aes(x=Generation, y=CLR, fill=AbbTax))+
  #geom_boxplot()+
  geom_point(aes(colour=AbbTax))+
  geom_line(aes(group=AbbTax, colour=AbbTax))+
  #geom_line(aes(colour=Taxa))+
  #facet_wrap(~Cycle, scales="free_y")+
```

```
  theme(panel.grid.major = element_blank(), panel.grid.minor =
element_blank(), panel.background = element_blank(), axis.line =
element_line(colour="black"))

NE_clr<-as.data.frame(d.clr) %>%
  rownames_to_column(var = "VarName") %>%
  separate(VarName, into = c("Community","Generation"),sep = "R3G0")
%>%
  filter(Community=="NE") %>%
  mutate(Generation = factor(Generation, levels = c("C1", "C2", "C3",
"C4", "C5",
                                                    "C6", "C7", "C8",
"C9", "C10",
                                                    "C11", "C12"))) %>%
  arrange(by_group=Generation) %>%
  pivot_longer(cols=3:8, names_to = "Taxa", values_to = "CLR") %>%
  left_join(TaxCode, by = "Taxa") %>%
  ggplot(aes(x=Generation, y=CLR, fill=AbbTax))+
  #geom_boxplot()+
  geom_point(aes(colour=AbbTax))+
  geom_line(aes(group=AbbTax, colour=AbbTax))+
  #geom_line(aes(colour=Taxa))+
  #facet_wrap(~Cycle, scales="free_y")+
  theme(panel.grid.major = element_blank(), panel.grid.minor =
element_blank(), panel.background = element_blank(), axis.line =
element_line(colour="black"))

##environmental offspring
PB_clr<-as.data.frame(d.clr) %>%
  rownames_to_column(var = "VarName") %>%
  separate(VarName, into = c("Community","Cycle"),sep = "R3G0") %>%
  filter(Community=="EE") %>%
  mutate(Cycle = factor(Cycle, levels = c("C1", "C2", "C3", "C4", "C5",
                                                    "C6", "C7", "C8",
"C9", "C10",
                                                    "C11", "C12"))) %>%
  arrange(by_group=Cycle) %>%
  pivot_longer(cols=3:8, names_to = "Taxa", values_to = "CLR") %>%
  left_join(TaxCode, by = "Taxa") %>%
  ggplot(aes(x=Cycle, y=CLR, fill=AbbTax))+
  #geom_boxplot()+
  geom_point(aes(colour=AbbTax))+
  geom_line(aes(group=AbbTax, colour=AbbTax))+
  #geom_line(aes(colour=Taxa))+
  #facet_wrap(~Cycle, scales="free_y")+
  theme(panel.grid.major = element_blank(), panel.grid.minor =
element_blank(), panel.background = element_blank(), axis.line =
element_line(colour="black"))

PR_clr<-as.data.frame(d.clr) %>%
  rownames_to_column(var = "VarName") %>%
  separate(VarName, into = c("Community","Cycle"),sep = "R3G0") %>%
  filter(Community=="NE") %>%
  mutate(Cycle = factor(Cycle, levels = c("C1", "C2", "C3", "C4", "C5",
                                                    "C6", "C7", "C8",
"C9", "C10",
                                                    "C11", "C12"))) %>%
  arrange(by_group=Cycle) %>%
```

```
  pivot_longer(cols=3:8, names_to = "Taxa", values_to = "CLR") %>%
  left_join(TaxCode, by = "Taxa") %>%
  ggplot(aes(x=Cycle, y=CLR, fill=AbbTax))+
  #geom_boxplot()+
  geom_point(aes(colour=AbbTax))+
  geom_line(aes(group=AbbTax, colour=AbbTax))+
  #geom_line(aes(colour=Taxa))+
  #facet_wrap(~Cycle, scales="free_y")+
  theme(panel.grid.major = element_blank(), panel.grid.minor =
element_blank(), panel.background = element_blank(), axis.line =
element_line(colour="black"))

OBE_clr<-as.data.frame(d.clr) %>%
  rownames_to_column(var = "VarName") %>%
  separate(VarName, into = c("Community","Cycle"),sep = "G1") %>%
  filter(Community=="T1R4") %>%
  mutate(Cycle = factor(Cycle, levels = c("C1", "C2", "C3", "C4", "C5",
                                                  "C6", "C7", "C8",
"C9", "C10"))) %>%
  arrange(by_group=Cycle) %>%
  pivot_longer(cols=3:8, names_to = "Taxa", values_to = "CLR") %>%
  left_join(TaxCode, by = "Taxa") %>%
  ggplot(aes(x=Cycle, y=CLR, fill=AbbTax))+
  #geom_boxplot()+
  geom_point(aes(colour=AbbTax))+
  geom_line(aes(group=AbbTax, colour=AbbTax))+
  #geom_line(aes(colour=Taxa))+
  #facet_wrap(~Cycle, scales="free_y")+
  theme(panel.grid.major = element_blank(), panel.grid.minor =
element_blank(), panel.background = element_blank(), axis.line =
element_line(colour="black"))

ORE_clr<-as.data.frame(d.clr) %>%
  rownames_to_column(var = "VarName") %>%
  separate(VarName, into = c("Community","Cycle"),sep = "G1") %>%
  filter(Community=="T5R3") %>%
  mutate(Cycle = factor(Cycle, levels = c("C1", "C2", "C3", "C4", "C5",
                                                  "C6", "C7", "C8",
"C9", "C10"))) %>%
  arrange(by_group=Cycle) %>%
  pivot_longer(cols=3:8, names_to = "Taxa", values_to = "CLR") %>%
  left_join(TaxCode, by = "Taxa") %>%
  ggplot(aes(x=Cycle, y=CLR, fill=AbbTax))+
  #geom_boxplot()+
  geom_point(aes(colour=AbbTax))+
  geom_line(aes(group=AbbTax, colour=AbbTax))+
  #geom_line(aes(colour=Taxa))+
  #facet_wrap(~Cycle, scales="free_y")+
  theme(panel.grid.major = element_blank(), panel.grid.minor =
element_blank(), panel.background = element_blank(), axis.line =
element_line(colour="black"))

#clr after time publication
as.data.frame(d.clr) %>%
  rownames_to_column(var = "VarName") %>%
  left_join(ppmp, by="VarName") %>%
  separate(VarName, into = c("Community","Cycle"),sep = "G0") %>%
  mutate(Cycle = factor(Cycle, levels = c("C1", "C2", "C3", "C4", "C5",
```

```
                                                   "C6", "C7", "C8", "C9",
"C10", "C11", "C12"))) %>%
  filter(Cycle!="NA") %>%
  arrange(by_group=Cycle) %>%
  pivot_longer(cols=3:10, names_to = "Taxa", values_to = "CLR") %>%
  left_join(TaxCode, by = "Taxa") %>%
  mutate(AbbTax=(gsub("Allorhizobium-Neorhizobium-Pararhizobium-
Rhizobium", "Rhizobiaceae", AbbTax))) %>%
  ggplot()+
  geom_col(aes(x=Cycle, y=ppmp), alpha=0.75, colour="darkgreen",
shape=23,size=2, show.legend = TRUE)+
  #geom_line(aes(x=Cycle, y=ppmp, group=Taxa, fill="soluble P"),
alpha=0.3, size=0.75, linetype=4, colour="darkgreen", show.legend =
TRUE)+
  geom_point(aes(x=Cycle, y=CLR, colour=AbbTax), size=2.2, alpha=0.75)+
  geom_line(aes(x=Cycle, y=CLR, group=AbbTax, colour=AbbTax),
alpha=0.4, size=1)+
  geom_hline(yintercept = 0, linetype = "dashed", colour="darkgrey")+
  scale_y_continuous(sec.axis = sec_axis(trans = ~ ., name =
"solubilized P (z-score transformed)"))+
  facet_wrap(~Community, ncol=1, scales="free_y")+
  geom_hline(yintercept = -1.96, linetype = "dotted",
colour="lightgrey")+
  geom_hline(yintercept = 1.96, linetype = "dotted",
colour="lightgrey")+
  theme(panel.grid.major = element_blank(), panel.grid.minor =
element_blank(), panel.background = element_blank(), axis.line =
element_line(colour="black"))

clr.time <- as.data.frame(d.clr) %>%
  rownames_to_column(var = "VarName") %>%
  left_join(ppmp, by="VarName") %>%
  separate(VarName, into = c("Community","Cycle"),sep = "G0") %>%
  mutate(Cycle = factor(Cycle, levels = c("C1", "C2", "C3", "C4", "C5",
                                                   "C6", "C7", "C8", "C9",
"C10", "C11", "C12"))) %>%
  filter(Cycle!="NA") %>%
  arrange(by_group=Cycle) %>%
  pivot_longer(cols=3:10, names_to = "Taxa", values_to = "CLR") %>%
  left_join(TaxCode, by = "Taxa") %>%
  mutate(AbbTax=(gsub("Allorhizobium-Neorhizobium-Pararhizobium-
Rhizobium", "Rhizobiaceae", AbbTax))) %>%
  ggplot()+
  geom_col(aes(x=Cycle, y=ppmp), alpha=0.75,  size=1, show.legend =
TRUE)+
  #geom_line(aes(x=Cycle, y=ppmp, group=Taxa, fill="soluble P"),
alpha=0.3, size=0.75, linetype=4, colour="darkgreen", show.legend =
TRUE)+
  geom_point(aes(x=Cycle, y=CLR, colour=AbbTax), size=2.2, alpha=0.75)+
  geom_line(aes(x=Cycle, y=CLR, group=AbbTax, colour=AbbTax),
alpha=0.4, size=1)+
  geom_hline(yintercept = 0, linetype = "dashed", colour="darkgrey")+
  scale_y_continuous(sec.axis = sec_axis(trans = ~ ., name =
"solubilized P (z-score transformed)"))+
  facet_wrap(~Community, ncol=1, scales="free_y")+
  geom_hline(yintercept = -1.96, linetype = "dotted",
colour="lightgrey")+
```

```
  geom_hline(yintercept = 1.96, linetype = "dotted",
colour="lightgrey")+
  theme(panel.grid.major = element_blank(), panel.grid.minor =
element_blank(), panel.background = element_blank(), axis.line =
element_line(colour="black"))




as.data.frame(d.clr) %>%
  rownames_to_column(var = "VarName") %>%
  left_join(ppmp, by="VarName") %>%
  separate(VarName, into = c("Community","Cycle"),sep = "G1") %>%
  mutate(Cycle = factor(Cycle, levels = c("C1", "C2", "C3", "C4", "C5",
                                          "C6", "C7", "C8", "C9",
"C10", "C11", "C12"))) %>%
  filter(Cycle!="NA") %>%
  arrange(by_group=Cycle) %>%
  pivot_longer(cols=3:10, names_to = "Taxa", values_to = "CLR") %>%
  left_join(TaxCode, by = "Taxa") %>%
  ggplot(aes(x=Cycle, y=CLR, fill=Community))+
  geom_point(aes(colour=Community, shape=Community), size=2.2,
alpha=0.75)+
  geom_line(aes(group=Community, colour=Community), alpha=0.4, size=1)+
  facet_grid(AbbTax~., scales="free_y")+
  geom_hline(yintercept = 0, linetype = "dashed", colour="darkgrey")+
  theme(panel.grid.major = element_blank(), panel.grid.minor =
element_blank(), panel.background = element_blank(), axis.line =
element_line(colour="black"))

#######GJAM Propagation######
####treating of PS###########
#7000taxa if we need all of them so we check

apply(tax_table(ps), 2, unique)
lapply(apply(tax_table(ps), 2, unique), length)
#Kingdom: 4
#Phylum: 37
#Class: 95
#Order: 182
#Family: 234
#Genus: 429
#Species:96

#filter from Kingoms
ps1<-subset_taxa(ps, Kingdom %in% c("Bacteria", "Archaea"))
apply(tax_table(ps1), 2, unique)

#filter further: NA in phylum, "Cyanobacteria" because they are likely
to be chloroplasts
ps2<-subset_taxa(ps1, Phylum!="Cyanobacteria")
ps3<-subset_taxa(ps2, !is.na(Phylum))

apply(tax_table(ps3), 2, unique)
lapply(apply(tax_table(ps3), 2, unique), length)
#Kingdom: 2
#Phylum: 35
#Class: 92
```

```
#Order: 178
#Family: 231
#Genus: 426
#Species:94

ps_Breeding2 <- subset_taxa(ps3, Experiment=="B2")
lapply(apply(tax_table(ps_Breeding2), 2, unique), length)
#Kingdom: 2
#Phylum: 30
#Class: 68
#Order: 126
#Family: 163
#Genus: 269
#Species:41

#######GJAM modelling Breeding2######
library(gjam)

xdata <- data.frame(sample_data(pdata)) %>%
  filter(Experiment=="B2")

xdata  <- data.frame(sample_data(pdata)) %>%
  #filter(Experiment != "F") %>%
  #filter(Experiment != "D") %>%
  filter(Experiment != "SR") %>%
  filter(Performance!="neg_control") %>%
  filter(Performance!="pos_control") %>%
  filter(Performance!="BlankpcrCES") %>%
  #filter(Performance!="B2") %>%
  #mutate(Performance = (gsub("R", "A", Performance))) %>%
  #mutate(Performance = (gsub("B1", "B", Performance))) %>%
  #filter(Generation!="G0") %>%
  filter(Timepoint!="EG1C1", Timepoint!="EG1C2", Timepoint!="EG1C3",
         Timepoint!="EG1C4", Timepoint!="EG1C5", Timepoint!="EG1C6",
Timepoint!="EG1C7",
         Timepoint!="EG1C8", Timepoint!="EG1C9", Timepoint!="EG1C10")
%>%
  dplyr::select(-Generation) %>%
  dplyr::rename(Generation=Experiment) %>%
  mutate(Generation = (gsub("B1", "E", Generation))) %>%
  mutate(Generation = (gsub("B2", "P", Generation)))

otu    <- data.frame(otu_table(pdata)) %>%
  filter(Experiment == "B2")
colnames(otu)<-TaxInfo$GenCode
ppmp  <- as.vector(xdata$ppmp)
otu_B2<-cbind(otu, ppmp)
ppmp  <- c(1.577408118, -0.37270571, 1.783905181, 1.15156508,
           6.326840561, 0.87442494, 2.816390495, -0.23413564,
           2.093650775, 5.30866723, 2.713141963, 1.370911055,
           0.45871472, 1.887153712, 2.196899306, 1.370911055,
           0.18157458, 1.267662524, 1.783905181, 0.73585486,
           1.783905181, -0.09556557, 1.68065665, -0.09556557,
           1.990402244, 0.04300451, 2.196899306, 1.29013515,
           1.783905181, -1.61983636, 1.68065665, 0.32014465,
           2.196899306, 1.84441544, 5.810597904, 0.87442494,
           0.854668399, 0.04300451, 2.196899306, -1.20412614,
           3.745627277, 1.01299501, 1.990402244, 0.32014465)
```

```
otu <- cbind(otu, ppmp)
otu    <- data.frame(otu_table(pdata)) %>%
  filter(Experiment == "B1")
otu_G0<-otu[!grepl("T", rownames(otu)),]
TaxInfo<-data.frame(tax_table(pdata)) %>%
  unite("GenInfor", Kingdom:Genus, sep="_", remove=FALSE) %>%
  mutate(GenCode=paste0("Tax", 1:nrow(. )))
otu<-rbind(otu_G0, otu)
otu_no2ndB<-otu[!grepl("B2", rownames(otu)),]
otu<-otu_no2ndB
View(otu)

hist( as.matrix(otu), ylab = "Reads", main = "OTU's")
#histogram looks weird HELP

min.ob<-ifelse(nrow(otu)*0.05<5,5, round(nrow(otu)*0.05,0))
nobs <- gjamTrimY( otu, minObs = min.ob, OTHER = F )$nobs   #minObs
either 5 or 5% of samples
hist( nobs/ncol(otu), nclass=100, xlab = 'Fraction of observations',
      ylab = 'Frequency', main='Incidence' ) #how common to find in
relation to all number of species



#trim by abundance instead of number of observations
tmp <- gjamTrimY(otu, minObs = min.ob)
y   <- tmp$y

TaxInfo %>%
  filter(!GenCode %in% colnames(y)) %>%
  apply(.,2,unique)

dim(otu)                      # all OTUs; 701
dim(y)                        # trimmed data; 7
tail(colnames(y))             # 'other' class added

#type names to microbial composition
ppmp  <- as.vector(xdata$ppmp)
ydata <- cbind(y, ppmp)
head(ydata)
ydata<-y
S     <- ncol(ydata)
typeNames    <- c(rep('CC',S-2), "CON", "CC")   # composition count
data and ppmp as continuous data

#set level for xdata; density
#xdata$Generation <- factor(xdata$Generation, levels = c("PG1", "PG2",
"PG3", "PG4", "PG5", "PG6", "PG7"))
xdata$Performance<-factor(xdata$Performance, levels = c("A", "B"))
xdata$Generation<-factor(xdata$Generation, levels=c("F", "O"))

#run model
ml <- list( ng = 15000, burnin = 5000, typeNames = typeNames, random =
"Timepoint")
output <- gjam(~ Generation*Performance, xdata, ydata, modelList = ml)
#1st variable is intercept, Performance
#check GJAM output with
View(output$parameters$betaTable)
```

```
#check model fit
fit <- gjamPlot(output, plotPars = list(GRIDPLOTS=T, SAVEPLOTS=TRUE) )


output_Breeding2_PP<-output
fit_Breeding2_PP<-fit

###diagnosis of MCMC chains to see if model is okay to continue

#Add here your settings for Gibbs sampling
ng <- 15000
burning <- 5000

#Add here you output from the GJAM analysis
selected.model <- output_CompBreeding

plot(cumsum(selected.model$chains$bgibbs[,1]-
mean(selected.model$chains$bgibbs[,1])),
     type="l", xlab="iteration", ylab="y")
#plot(selected.model$chains$bgibbsUn[,1]-
mean(selected.model$chains$bgibbsUn[,1]),
#    type="l", xlab="iteration", ylab="y")

plot(selected.model$chains$bgibbs[,1])

# center with 'apply()'
center_apply <- function(x) {
  apply(x, 2, function(y) y - mean(y))
}

# apply it
dfplot <-
data.frame(center_apply(data.frame(selected.model$chains$bgibbs)))
#dfplot <-
data.frame(center_apply(data.frame(selected.model$chains$bgibbs[burning
:ng,])))
#dfplot <-
data.frame(center_apply(data.frame(selected.model$chains$sgibbs)))

plot(dfplot[,5],type="l", xlab="iteration", ylab="y")
dim(dfplot)

#bgibbs
DiagTab <- dfplot %>%
  #  data.frame() %>%
  mutate(Iteration = 1:nrow(dfplot)) %>%
  gather(key = "Parameter", value = "value", -Iteration) %>%
  # filter(Iteration>burning) %>%
  group_by(Parameter) %>%
  summarise(Median = mean(value, na.rm = TRUE), CIlow = quantile(value,
0.025, na.rm = TRUE),
            CIhigh = quantile(value, 0.975, na.rm = TRUE),IQR =
IQR(value)) %>%
  ungroup() %>%
  filter(IQR>quantile(IQR,0.975)) %>% #Q3
  # filter(IQR>10) %>%
```

```
  separate(Parameter, into = c("Taxa","Treatment"),sep = "_", remove =
FALSE)

#sgibbs
#DiagTab <- dfplot %>%
#  data.frame() %>%
# mutate(Iteration = 1:nrow(dfplot)) %>%
# gather(key = "Parameter", value = "value", -Iteration) %>%
# filter(Iteration>burning) %>%
# group_by(Parameter) %>%
# summarise(Median = mean(value, na.rm = TRUE), CIlow = quantile(value,
0.025, na.rm = TRUE),
#          CIhigh = quantile(value, 0.975, na.rm = TRUE),IQR =
IQR(value)) %>%
#ungroup() %>%
#filter(IQR>quantile(IQR,0.975))

dim(DiagTab)
View(DiagTab)    #DiagTab gives the samples with the highest divergent
summary(DiagTab)

summary(DiagTab$IQR)

hist(DiagTab$IQR)
boxplot(DiagTab$IQR)     #important to see in which range the variation
happens; if small go on
length(unique(DiagTab$Parameter))
Notconverged <- unique(DiagTab$Parameter)
unique(DiagTab$Taxa)

plot(dfplot[,Notconverged[1]]-mean(dfplot[,Notconverged[1]]),type="l",
xlab="iteration", ylab="y")
plot(cumsum(dfplot[,Notconverged[1]]),type="l", xlab="iteration",
ylab="y")

View(selected.model$inputs$y[,unique(DiagTab$Taxa)])
heatmap(scale(selected.model$inputs$y[,unique(DiagTab$Taxa)]))
apply(selected.model$inputs$y[,unique(DiagTab$Taxa)],2,hist)

library(ggplot2)
#Looking to the chains that did not converged
dfplot %>%
  # mutate_all(list(cumsum)) %>%
  #  data.frame() %>%
  mutate(ng = 1:nrow(dfplot)) %>%
  # filter(ng>burning) %>%
  tidyr::gather(key = VarName, value = MCMC, -ng) %>%
  # filter(VarName %in% sample(Notconverged,20)) %>%
  filter(VarName %in% Notconverged) %>% #If smaller than 20
  ggplot(aes(x = ng, y = MCMC, color = VarName))+
  geom_line()+
  # scale_y_log10()+
  theme(panel.grid.major = element_blank(), panel.grid.minor =
element_blank(), panel.background = element_blank(), axis.line =
element_line(colour="black"))


#Randomly checking the converged chains
```

```
SampleRandom <- sample(colnames(dfplot),20)
dfplot %>%
  #  data.frame() %>%
  mutate(ng = 1:nrow(dfplot)) %>%
  filter(ng>burning) %>%
  tidyr::gather(key = VarName, value = MCMC, -ng) %>%
  #filter(VarName %in% SampleRandom) %>%
  ggplot(aes(x = ng, y = MCMC, color = VarName))+
  geom_line()+
  #theme(panel.grid.major = element_blank(), panel.grid.minor =
element_blank(), panel.background = element_blank(), axis.line =
element_line(colour="black"))
  #theme(legend.position = "none")
  theme(panel.grid.major = element_blank(), panel.grid.minor =
element_blank(), panel.background = element_blank(), axis.line =
element_line(colour="black"))


dfplot %>%
  #  data.frame() %>%
  mutate(ng = 1:nrow(dfplot)) %>%
  filter(ng>burning) %>%
  tidyr::gather(key = VarName, value = MCMC, -ng) %>%
  filter(VarName %in% SampleRandom) %>%
  ggplot(aes(x = VarName, y = MCMC, color = VarName))+
  geom_boxplot()+
  theme(legend.position = "none")

#if stable save data for next step
save(output_Breeding2_PP,file="gjamOutput_Breeding2_PP.RData")

#########Analysis of GJAM output####
#task split up betastandxwtable into two columns (intercept &
treatment) and plot it (dyplr and ggplot)
beta.table<- data.frame(output_Breeding2_PP$parameters$betaTable)

#try to minimize code
new_beta<- beta.table %>%
  rownames_to_column(var = "row_name") %>%
  separate(row_name, into = c("Taxa","Treatment"),sep = "_") %>%
  dplyr::select(-c(CI_025,CI_975, SE, sig95)) %>%
  pivot_wider(names_from= "Treatment", values_from=Estimate) %>%
  filter(Taxa!="ppmp") %>%
  ggplot(aes(x=intercept, y=PerformanceB2))+
  geom_point()


#filter for significance and link phylogenetic information
Intercept_df<-beta.table %>%
  rownames_to_column(var = "row_name") %>%
  separate(row_name, into = c("Taxa","Treatment"),sep = "_") %>%
  filter(Treatment=="intercept") %>%
  dplyr::select(-c(CI_025,CI_975, SE, sig95)) %>%
  pivot_wider(names_from= "Treatment", values_from=Estimate)

Taxa_Info<-TaxInfo %>%
  rename(Taxa=GenCode) %>%
  #filter(TaxVar %in% unique(filteredSign$TaxVar)) %>%
```

```
  mutate(AbbTax = paste(Genus,Species)) %>%
  mutate(AbbTax = gsub("NA","un",AbbTax)) %>%
  mutate(AbbTax = ifelse(AbbTax == "un un", paste(Family, "un"),
AbbTax)) %>%
  mutate(AbbTax = ifelse(AbbTax == "NA un", paste(Order, "un"),
AbbTax)) %>%
  mutate(AbbTax = ifelse(AbbTax == "NA un", paste(Class, "un"),
AbbTax)) %>%
  mutate(AbbTax = ifelse(AbbTax == "NA un", paste(Phylum, "un"),
AbbTax))


beta.table %>%
  rownames_to_column(var = "row_name") %>%
  separate(row_name, into = c("Taxa","Treatment"),sep = "_") %>%
  filter(Treatment=="PerformanceB2") %>%
  dplyr::select(-c(CI_025,CI_975, SE)) %>%
  filter(sig95=="*") %>%
  pivot_wider(names_from= "Treatment", values_from=Estimate) %>%
  left_join(Intercept_df, by=c("Taxa")) %>%
  left_join(Taxa_Info, by=c("Taxa")) %>%
  filter(Taxa != "ppmp") %>%
  ggplot(aes(x=intercept, y=PerformanceB2, color = AbbTax))+
  geom_point(aes(size=PerformanceB2))+
  geom_hline(aes(yintercept=0), linetype = "dashed")+
  geom_vline(aes(xintercept = 0), linetype = "dashed")+
  theme(panel.grid.major = element_blank(), panel.grid.minor =
element_blank(), panel.background = element_blank(), axis.line =
element_line(colour="black"))+
  labs(x="intercept", y="best performing", title="significant second-
best performing genera")


####One way analysis####
selected.model<-output_Breeding2
selected.model$inputs$y
ynames <- colnames(selected.model$inputs$y)
ynames
View(TaxInfo[TaxInfo$GenCode %in% ynames,])

#Coefficients
heatmap(selected.model$parameters$betaStandXWmu)
library("gplots")
netTab <- selected.model$parameters$betaStandXWmu
hT <- heatmap.2(netTab, scale = "none", col = rev(bluered(100)), cexRow
=1, cexCol=1, #lhei=lhei, lwid=lwid,lmat =lmat,
                trace = "none", density.info = "none", labCol =
LabelsCode$AbbTax)


LabelsCode <- data.frame(GenCode = colnames(netTab)) %>%
  left_join(TaxInfo,by = c("GenCode")) %>%
  mutate(AbbTax = paste(Genus)) %>%
  mutate(AbbTax = gsub("NA","un",AbbTax)) %>%
  mutate(AbbTax = ifelse(AbbTax == "un", paste(Family), AbbTax)) %>%
  mutate(AbbTax = ifelse(AbbTax == "NA", paste(Order), AbbTax)) %>%
  mutate(AbbTax = ifelse(AbbTax == "NA", paste(Class), AbbTax)) %>%
  mutate(AbbTax = ifelse(AbbTax == "NA", paste(Phylum), AbbTax)) %>%
```

```
  mutate(AbbTax=ifelse(GenCode=="ppmp", paste("soluble P"), AbbTax))


dendVar <- hT$colDendrogram
labels(dendVar) <- LabelsCode$AbbTax
plot(as.hclust(dendVar), hang = -1, cex = 0.6)



#Null significant effect
coeffTabfinal <- selected.model$parameters$betaStandXWTable
NullSigEff <- coeffTabfinal %>%
  mutate(VarEffect =
rownames(selected.model$parameters$betaStandXWTable)) %>%
  tidyr::separate(VarEffect, into = c("DepVar", "Treat"), sep = "_")
%>%
  #tidyr::separate(DepVar, into = c("Phylum", "Class")) %>%
  # mutate(DepVar = ifelse(DepVar == "HAl", "H_Al", DepVar)) %>% #View
  mutate(DepVar = factor(DepVar, levels = unique(DepVar))) %>%
  filter(sig95 == "*") %>%
  filter(DepVar!="ppmp")

View(NullSigEff)

LabelsCode.sig<-LabelsCode %>%
  filter(GenCode %in% NullSigEff$DepVar)

unique(NullSigEff$DepVar)
heatmap.2(netTab[,unique(NullSigEff$DepVar)], scale = "none", col =
rev(bluered(100)),cexCol = 1, cexRow =1,#lhei=lhei, lwid=lwid,lmat
=lmat,
          trace = "none", density.info = "none", labCol =
LabelsCode.sig$AbbTax)

###Bubble Plot###
library(zCompositions)
selected.model<-output_Breeding2
ydata<-selected.model$inputs$y
d.czm <- cmultRepl(ydata[,1:6],  label=0, method="CZM")
colnames(d.czm)
# The table needs to be transposed again (samples as COLUMNS)
#log - log means division ; relative abudnance
d.clr <- t(apply(d.czm, 1, function(x){log(x) - mean(log(x))}))

ControlData <- cbind(output_Breeding2$inputs$xdata, d.clr) %>% #View
  gather(key = "GenCode", value = "CLR",
contains("Tax"),contains("other")) %>%
  left_join(TaxInfo, by = "GenCode") %>% #View
  filter(Performance=="A") %>%
  mutate(AbbTax = paste(Genus,Species)) %>%
  mutate(AbbTax = gsub("NA","un",AbbTax)) %>%
  mutate(AbbTax = ifelse(AbbTax == "un un", paste(Order, "un"),
AbbTax)) %>%
  mutate(AbbTax = ifelse(AbbTax == "NA un", paste(Class, "un"),
AbbTax)) %>%
  dplyr::select(-SampleID) %>% #colnames()
  group_by(Kingdom, Phylum, Class, Order, Family, Genus, Species,
AbbTax,Performance,  GenCode) %>%
```

```
  summarise(CLR.mean = mean(CLR)) %>%
  ungroup() %>%
  mutate(Performance = gsub("S", "", Performance)) %>%
  rename(DepVar=GenCode)
View(ControlData)


#Buble plot
#RtN0
#t(EstimateEffRtN0[,-c(1,8)])
CoeffValues <- NullSigEff %>%
  dplyr::select(Estimate,DepVar, Treat) %>%
  mutate(Treatment = gsub("Performance", "", Treat)) %>%
  filter(Treatment != "A")


CoeffValues %>%
  mutate(Size = ifelse(Estimate<0, -1*Estimate,Estimate)) %>%
  left_join(ControlData, by = "DepVar") %>% #View
  mutate(NameG = ifelse(Estimate %in%
tail(sort(CoeffValues$Estimate),5), AbbTax, NA)) %>% #View
  mutate(NameG = ifelse(Estimate %in%
head(sort(CoeffValues$Estimate),5), AbbTax, NameG)) %>% #View
  mutate(Treatment = factor(Treatment, levels = c("B1", "B2"))) %>%
  ggplot(aes(x=CLR.mean, y=Estimate, size=Size,
             color=AbbTax)) +
  geom_point(alpha=0.5) +
  facet_wrap(~Treatment, scales = "free")+
  geom_text(aes(label=NameG, size = 2),hjust=0.1, vjust=0.1)+
  geom_hline(yintercept = 0, linetype = "dashed")+
  geom_vline(xintercept = 0, linetype = "dashed")+
  theme_bw()


#####Factorial GJAM#####
library(gjam)
selected.model <- output_Breeding2_PP
dim(selected.model$chains$bgibbs)
ExName <- colnames(selected.model$parameters$betaMu)[1]
# paste0(TaxaSelected,Fact1Name,unique(fact1))
library(dplyr)
ng <- 15000
fact1 <- "Generation"
fact2 <- "Performance"
df.gibbs3 <- GJAM.Factorial(selected.model,F1Name = fact1,F2Name =
fact2,
                            Taxa=ExName, ng=ng,burning=ng-100)
colnames(df.gibbs3)

df.gibbs3 %>%
  gather(key = "VarName", value = "Coeff") %>%
  separate(VarName, into = c("Fact1", "Fact2"), sep = "_", extra =
"merge") %>%
  ggplot(aes(x = Fact1, y = Coeff, fill = Fact2)) +
  geom_boxplot()+
  geom_hline(yintercept = 0, linetype = "dashed")+
  # scale_x_discrete(limits =fact1)+
  theme_bw()
```

```
df.gibbs3 %>%
  gather(key = "VarName", value = "Coeff") %>%
  separate(VarName, into = c("Fact1", "Fact2"), sep = "_", extra =
"merge") %>%
  ggplot(aes(x = Fact1, y = Coeff, fill = Fact2)) +
  geom_boxplot(outlier.size = 0) +
  stat_summary(fun="mean", geom="point", shape=23, size=4,
fill="white") +
  #guides(fill=FALSE)+
  geom_hline(yintercept = 0, linetype = "dashed")+
  # scale_x_discrete(limits =fact1)+
  theme_bw()

quantiles_95 <- function(x) {
  r <- quantile(x, probs=c(0.025, 0.25, 0.5, 0.75, 0.975))
  names(r) <- c("ymin", "lower", "middle", "upper", "ymax")
  r
}

lvF1 <- levels(as.data.frame(selected.model$inputs$xdata)[,fact1])
lvF2 <- levels(as.data.frame(selected.model$inputs$xdata)[,fact2])

df.gibbs3 %>%
  gather(key = "VarName", value = "Coeff") %>%
  separate(VarName, into = c("Fact1", "Fact2"), sep = "_", extra =
"merge") %>%
  mutate(Fact1 = factor(Fact1, levels = lvF1)) %>%
  mutate(Fact2 = factor(Fact2, levels = lvF2)) %>%
  ggplot(aes(x = Fact2, y = Coeff, fill = Fact1)) +
  # guides(fill=F) +
  stat_summary(fun.data = quantiles_95, geom="boxplot",position =
"dodge")+
  stat_summary(fun="median", geom="point", shape=23, size=4,
fill="white") +
  stat_smooth(method="lm", formula=y~1, se=FALSE, aes(group = 1)) +
  # stat_summary(fun.y = "mean", color = "red", geom = "line",
aes(group = 1))+
  # geom_hline(yintercept = 0, linetype = "dashed")+
  # scale_x_discrete(limits =fact1)+
  theme_bw()

df.gibbs3 %>%
  gather(key = "VarName", value = "Coeff") %>%
  separate(VarName, into = c("Fact1", "Fact2"), sep = "_", extra =
"merge") %>%
  mutate(Fact1 = factor(Fact1, levels = lvF1)) %>%
  mutate(Fact2 = factor(Fact2, levels = lvF2)) %>%
  ggplot(aes(x = Fact1, y = Coeff, fill = Fact2)) +
  # guides(fill=F) +
  stat_summary(fun.data = quantiles_95, geom="boxplot",position =
"dodge")+
  stat_summary(fun="median", geom="point", shape=23, size=4,
fill="white") +
  stat_smooth(method="lm", formula=y~1, se=FALSE, aes(group = 1)) +
  # stat_summary(fun.y = "mean", color = "red", geom = "line",
aes(group = 1))+
  # geom_hline(yintercept = 0, linetype = "dashed")+
```

```
  # scale_x_discrete(limits =fact1)+
  theme_bw()

barplot(apply(df.gibbs3,2,mean))
barplot(selected.model$parameters$betaMu[,1])

multinames <- function(varnames){
  print(varnames)
  res <- GJAM.Factorial(output = selected.model,
                        F1Name = fact1,F2Name = fact2,
                        Taxa=varnames, ng=ng,burning=ng-100)
  return(res)
}

Fact.Res <-
lapply(colnames(selected.model$parameters$betaMu),multinames)
names(Fact.Res) <- colnames(selected.model$parameters$betaMu)
titleName <- names(Fact.Res[2])
Fact.Res[[2]]  %>%
  gather(key = "VarName", value = "Coeff") %>%
  separate(VarName, into = c("Fact1", "Fact2"), sep = "_", extra =
"merge") %>%
  mutate(Fact1 = factor(Fact1, levels = lvF1)) %>%
  mutate(Fact2 = factor(Fact2, levels = lvF2)) %>%
  ggplot(aes(x = Fact2, y = Coeff, fill = Fact1)) +
  # guides(fill=F) +
  stat_summary(fun.data = quantiles_95, geom="boxplot",position =
"dodge")+
  stat_summary(fun="median", geom="point", shape=23, size=4,
fill="white") +
  stat_smooth(method="lm", formula=y~1, se=FALSE, aes(group = 1)) +
  # stat_summary(fun.y = "mean", color = "red", geom = "line",
aes(group = 1))+
  geom_hline(yintercept = 0, linetype = "dashed")+
  # ggtitle(titleName)+
  # scale_x_discrete(limits =fact1)+
  theme_bw()

Fact.Res[[2]]  %>%
  gather(key = "VarName", value = "Coeff") %>%
  separate(VarName, into = c("Fact1", "Fact2"), sep = "_", extra =
"merge") %>%
  mutate(Fact1 = factor(Fact1, levels = lvF1)) %>%
  mutate(Fact2 = factor(Fact2, levels = lvF2)) %>%
  ggplot(aes(x = Fact2, y = Coeff, fill = Fact1)) +
  stat_summary(fun.data = quantiles_95, geom="boxplot",position =
"dodge")+
  stat_summary(fun="median", geom="point", shape=23, size=4,
fill="white") +
  stat_smooth(method="lm", formula=y~1, se=FALSE, aes(group = 1)) +
  # stat_summary(fun.y = "mean", color = "red", geom = "line",
aes(group = 1))+
  geom_hline(yintercept = 0, linetype = "dashed")+
  # scale_x_discrete(limits =fact1)+
  theme_bw()

#All Variables
do.call(rbind,Fact.Res[1:2])
```

```
Fact.Res.DF <- data.frame(TaxVar =
rep(names(Fact.Res),each=nrow(data.frame(Fact.Res[1]))),
                          do.call(rbind,Fact.Res))
View(Fact.Res.DF)

#select specific taxas
TaxVarS <- unique(Fact.Res.DF$TaxVar)
Fact.Res.DF  %>%
  gather(key = "VarName", value = "Coeff", -TaxVar) %>%
  filter(TaxVar %in% TaxVarS[1:9]) %>% #analisa de 1 a 9
  separate(VarName, into = c("Fact1", "Fact2"), sep = "_", extra =
"merge") %>%
  mutate(Fact1 = factor(Fact1, levels = lvF1)) %>%
  mutate(Fact2 = factor(Fact2, levels = lvF2)) %>%
  ggplot(aes(x = Fact1, y = Coeff, fill = Fact2)) +
  #guides(fill="none") +
  stat_summary(fun.data = quantiles_95, geom="boxplot",position =
"dodge")+
  stat_summary(fun="median", geom="point", shape=23, size=4,
fill="white") +
  stat_smooth(method="lm", formula=y~1, se=FALSE, aes(group = 1)) +
  # stat_summary(fun.y = "mean", color = "red", geom = "line",
aes(group = 1))+
  geom_hline(yintercept = 0, linetype = "dashed")+
  # scale_x_discrete(limits =fact1)+
  facet_wrap(~TaxVar)+
  theme_bw()

Fact.Res.DF[,paste0(lvF1[1],"_",lvF2[1])]

tH <- Fact.Res.DF  %>% # estimate the coefficients
  gather(key = "VarName", value = "Coeff", -TaxVar) %>%
  separate(VarName, into = c("Fact1", "Fact2"), sep = "_", extra =
"merge", remove = FALSE) %>% #View
  group_by(TaxVar,Fact1,Fact2,VarName) %>%
  summarise(Mean = mean(Coeff),Q1 = quantile(Coeff, 0.025),
            Q3 = quantile(Coeff, 0.975)) %>%
  ungroup()

tH0 <- tH %>%
  group_by(TaxVar) %>%
  summarise(H0 = mean(Mean)) %>%
  ungroup()

tHF1 <- tH %>%
  group_by(TaxVar,Fact1) %>%
  summarise(H0F1 = mean(Mean)) %>%
  ungroup()

tHF2 <- tH %>%
  group_by(TaxVar,Fact2) %>%
  summarise(H0F2 = mean(Mean)) %>%
  ungroup()

tHcF1 <- Fact.Res.DF  %>% # estimate the coefficients
  gather(key = "VarName", value = "Coeff", -TaxVar) %>%
  separate(VarName, into = c("Fact1", "Fact2"), sep = "_", extra =
"merge", remove = FALSE) %>% #View
```

```
  group_by(TaxVar,Fact1,Fact2,VarName) %>%
  summarise(Mean = mean(Coeff),Q1 = quantile(Coeff, 0.025),
            Q3 = quantile(Coeff, 0.975)) %>%
  ungroup() %>%
  filter(Fact1 == lvF1[1]) %>%
  dplyr::select(TaxVar,Fact2,Mean) %>%
  rename(HcF1 = Mean)

tHcF2 <- Fact.Res.DF  %>% # estimate the coefficients
  gather(key = "VarName", value = "Coeff", -TaxVar) %>%
  separate(VarName, into = c("Fact1", "Fact2"), sep = "_", extra =
"merge", remove = FALSE) %>% #View
  group_by(TaxVar,Fact1,Fact2,VarName) %>%
  summarise(Mean = mean(Coeff),Q1 = quantile(Coeff, 0.025),
            Q3 = quantile(Coeff, 0.975)) %>%
  ungroup() %>%
  filter(Fact2 == lvF2[1]) %>%
  dplyr::select(TaxVar,Fact1,Mean) %>%
  rename(HcF2 = Mean)

tHF <- tH %>%
  left_join(tH0, by = "TaxVar") %>%
  left_join(tHF1, by = c("TaxVar","Fact1")) %>%
  left_join(tHF2, by = c("TaxVar","Fact2")) %>%
  left_join(tHcF1, by = c("TaxVar","Fact2")) %>%
  left_join(tHcF2, by = c("TaxVar","Fact1")) %>%
  mutate(SignH0 = ifelse(Q1<=H0 & Q3>=H0, "", "*")) %>%  #If any
difference between treatments
  mutate(SignH0F1 = ifelse(Q1<=H0F1 & Q3>=H0F1, "", "*")) %>% #if any
difference between Fact1 treatments
  mutate(SignH0F2 = ifelse(Q1<=H0F2 & Q3>=H0F2, "", "*")) %>%  #if any
difference between Fact2 treatments
  mutate(SignHcF1 = ifelse(Q1<=HcF1 & Q3>=HcF1, "", "*")) %>%  #if
treatments in Fact1 differs from the control within Fact2
  mutate(SignHcF2 = ifelse(Q1<=HcF2 & Q3>=HcF2, "", "*")) #if
treatments in Fact2 differs from the control within Fact1

tHF_Breeding2<-tHF

SigVarsH0 <- as.vector(unique(tHF[tHF$SignH0=="*","TaxVar"]))
SigVarsH0F1 <- as.vector(unique(tHF[tHF$SignH0F1=="*","TaxVar"]))
SigVarsH0F2 <- as.vector(unique(tHF[tHF$SignH0F2=="*","TaxVar"]))
SigVarsHcF1 <- as.vector(unique(tHF[tHF$SignHcF1=="*","TaxVar"]))
SigVarsHcF2 <- as.vector(unique(tHF[tHF$SignHcF2=="*","TaxVar"]))

#Take the first 9 significant ones
SignTax <- as.vector(SigVarsH0$TaxVar)
SignTax <- as.vector(SigVarsH0F1$TaxVar)
SignTax <- as.vector(SigVarsH0F2$TaxVar)
SignTax <- as.vector(SigVarsHcF1$TaxVar)
SignTax <- as.vector(SigVarsHcF2$TaxVar)
SignTax

SignSign <- tHF %>%
  mutate(Fact1 = factor(Fact1, levels = lvF1)) %>%
  mutate(Fact2 = factor(Fact2, levels = lvF2)) %>% #View
  #filter(TaxVar %in% SignTax) %>%
  filter(SignHcF1=="*") %>%
```

```
  mutate(SignPosition = ifelse(SignHcF2 == "*", Q3,NA))
summary(filteredSign)

Fact.Res.DF  %>%
  gather(key = "VarName", value = "Coeff", -TaxVar) %>%
  #filter(TaxVar %in% filteredTaxa) %>%
  separate(VarName, into = c("Fact1", "Fact2"), sep = "_", extra =
"merge") %>%
  left_join(filteredSign,by=c("TaxVar", "Fact1", "Fact2")) %>%
  mutate(Fact1 = factor(Fact1, levels = lvF1)) %>%
  mutate(Fact2 = factor(Fact2, levels = lvF2)) %>% #View
  ggplot(aes(x = Fact1, y = Coeff, fill = Fact2)) +
  #guides(fill="none") +
  stat_summary(fun.data = quantiles_95, geom="boxplot",position =
"dodge")+
  stat_summary(fun="mean", geom="point", shape=23, size=4,
fill="white") +
  stat_smooth(method="lm", formula=y~1, se=FALSE, aes(group = 1)) +
  # stat_summary(fun.y = "mean", color = "red", geom = "line",
aes(group = 1))+
  geom_hline(yintercept = 0, linetype = "dashed")+
  # scale_x_discrete(limits =fact1)+

#geom_text(data=filteredSign,aes(x=Fact1,group=Fact2,y=SignPosition,lab
el=SignHcF2),
  #        size=5, position = position_dodge(width=0.9))+
  # geom_text(aes(label = SignHcF1,x=Fact1, y=Q3),label="*", position =
"fill")+
  facet_wrap(~TaxVar,scales = "free_y")+
  theme_bw()

#Significant Effects within Factor 2
filteredTaxa <- SignTax[5:13]
filteredSign <- tHF %>%
  mutate(Fact1 = factor(Fact1, levels = lvF1)) %>%
  mutate(Fact2 = factor(Fact2, levels = lvF2)) %>% #View
  #filter(TaxVar %in% filteredTaxa) %>%
  mutate(SignPosition = ifelse(SignHcF1 == "*", Q3,NA))

Fact.Res.DF  %>%
  gather(key = "VarName", value = "Coeff", -TaxVar) %>%
  filter(TaxVar %in% filteredTaxa) %>%
  separate(VarName, into = c("Fact1", "Fact2"), sep = "_", extra =
"merge") %>%
  left_join(filteredSign,by=c("TaxVar", "Fact1", "Fact2")) %>%
  mutate(Fact1 = factor(Fact1, levels = lvF1)) %>%
  mutate(Fact2 = factor(Fact2, levels = lvF2)) %>% #View
  ggplot(aes(x = Fact2, y = Coeff, fill = Fact1)) +
  #guides(fill="none") +
  stat_summary(fun.data = quantiles_95, geom="boxplot",position =
"dodge")+
  stat_summary(fun="mean", geom="point", shape=23, size=4,
fill="white") +
  stat_smooth(method="lm", formula=y~1, se=FALSE, aes(group = 1)) +
  # stat_summary(fun.y = "mean", color = "red", geom = "line",
aes(group = 1))+
  geom_hline(yintercept = 0, linetype = "dashed")+
  # scale_x_discrete(limits =fact1)+
```

```
geom_text(data=filteredSign,aes(x=Fact2,group=Fact1,y=SignPosition,labe
l=SignHcF1),
            size=5, position = position_dodge(width=1))+
  #geom_text(aes(label = SignHcF1,x=Fact1, y=Q3),label="*", position =
"fill")+
  facet_wrap(~TaxVar,scales = "free_y")+
  theme_bw()

TaxCode <- TaxInfo %>%
  rename(TaxVar=GenCode)
Fact.Res.DF  %>%
  filter(TaxVar %in% unique(filteredSign$TaxVar)) %>% #dim()
  gather(key = "VarName", value = "Coeff", -TaxVar) %>% #View()
  group_by(TaxVar,VarName) %>%
  summarise(median = quantiles_95(Coeff)[3],
            ci_lower=quantiles_95(Coeff)[1],
            ci_upper=quantiles_95(Coeff)[5]) %>% #View
  separate(VarName, into = c("Fact1", "Fact2"), sep = "_", extra =
"merge") %>%
  # separate(TaxVar, into = c("Kingdom", "Phylum", "Class", "Order",
"Family", "Genus"), remove = FALSE,
  #          sep = "\\.", fill = "left", extra = "merge") %>%
  # mutate(AllVar = ifelse(Genus == "g",paste0(Family,Genus),Genus))
%>%
  # mutate(AllVar = ifelse(AllVar == "fg", paste0(Order,AllVar),
AllVar)) %>% #View
  mutate(Fact1 = factor(Fact1, levels = lvF1)) %>%
  mutate(Fact2 = factor(Fact2, levels = lvF2)) %>%
  ggplot(aes(x = TaxVar, y = median, fill = Fact2)) +
  #guides(fill=F) +
  # geom_boxplot() +
  #stat_summary(fun.data = quantiles_95, geom="boxplot",position =
"dodge")+
  geom_hline(yintercept = 0, linetype = "dashed")+
  geom_linerange(aes(ymin=ci_lower, ymax=ci_upper), position =
position_dodge(width = 0.75))+
  geom_point(size=1, position = position_dodge(width = 0.75))+
  # scale_x_discrete(limits =fact1)+

#geom_text(data=filteredSign,aes(x=TaxVar,group=Fact2,y=SignPosition,la
bel=SignHcF2),
  #         size=5, position = position_dodge(width=0.9))+
  facet_wrap(~Fact1, ncol = 2, scales = "free_x")+
  coord_flip()+
  theme_bw()

#regression coefficients with taxinfo
#all sig Taxa within factor 1
filteredSign <- tHF %>%
  mutate(Fact1 = factor(Fact1, levels = lvF1)) %>%
  mutate(Fact2 = factor(Fact2, levels = lvF2)) %>% #View
  filter(SignH0F1=="*")

filteredSignT <- tHF %>%
  mutate(SignHcF2 = ifelse(SignHcF2 == "*", "#", SignHcF2)) %>%
  mutate(SignHcF1F2 = paste0(SignHcF1,SignHcF2)) %>%
  filter(SignHcF1F2!="")
```

```
TaxCode <- TaxInfo %>%
  rename(TaxVar=GenCode) %>%
  #filter(TaxVar %in% unique(filteredSign$TaxVar)) %>%
  mutate(AbbTax = paste(Genus)) %>%
  mutate(AbbTax = gsub("NA","un",AbbTax)) %>%
  mutate(AbbTax = ifelse(AbbTax == "un", paste(Family), AbbTax)) %>%
  mutate(AbbTax = ifelse(AbbTax == "NA", paste(Order), AbbTax)) %>%
  mutate(AbbTax = ifelse(AbbTax == "NA", paste(Class), AbbTax)) %>%
  mutate(AbbTax = ifelse(AbbTax == "NA", paste(Phylum), AbbTax))

Fact.Res.DF  %>%
  filter(TaxVar %in% unique(filteredSign$TaxVar)) %>% #dim()
  gather(key = "VarName", value = "Coeff", -TaxVar) %>% #View()
  filter(TaxVar!="ppmp") %>%
  group_by(TaxVar,VarName) %>%
  summarise(median = quantiles_95(Coeff)[3],
            ci_lower=quantiles_95(Coeff)[1],
            ci_upper=quantiles_95(Coeff)[5]) %>%
  mutate(Sig = ifelse(ci_lower<=0 & ci_upper>=0, "", "*")) %>%
  filter(Sig=="*") %>%
  left_join(TaxCode, by = "TaxVar") %>%
  filter(AbbTax!="NA") %>%
  ggplot(aes(x = AbbTax, y = median)) +
  geom_hline(yintercept = 0, linetype = "dashed")+
  geom_linerange(aes(ymin=ci_lower, ymax=ci_upper, color = ci_upper<0),
position = position_dodge(width = 0.75))+
  geom_point(aes(color = median<0), size=1, position =
position_dodge(width = 0.75))+
  facet_wrap(~VarName, ncol = 4, scales = "free_x")+
  guides(color="none") +
  coord_flip()+
  labs(y="Regression coefficients", x= NULL)+
  theme(panel.grid.major = element_blank(), panel.grid.minor =
element_blank(),
        panel.background = element_blank(), axis.line =
element_line(colour="black"),
        axis.text = element_text(size=8))

##factorial gjam heatmaps
#heatmap for factor 1
heatmapTab <- tHF %>%
  dplyr::select(TaxVar,VarName,Mean) %>%
  spread(key = TaxVar,value = Mean, fill = 0) %>%
  dplyr::select(-other)
heatmapTabSign <- tHF %>%
  dplyr::select(TaxVar,VarName,SignHcF1) %>%
  spread(key = TaxVar,value = SignHcF1, fill = NA) %>%
  dplyr::select(-other)

heatmapTabSignT <- tHF %>%
  mutate(SignHcF2 = ifelse(SignHcF2 == "*", "#", SignHcF2)) %>%
  mutate(SignHcF1F2 = paste0(SignHcF1,SignHcF2)) %>%
  dplyr::select(TaxVar,VarName,SignHcF1F2) %>%
  spread(key = TaxVar,value = SignHcF1F2, fill = NA) %>%
  dplyr::select(-other)

LabelsCode <- data.frame(TaxVar = colnames(heatmapTab)) %>%
```

```
  left_join(TaxCode,by = c("TaxVar")) %>%
  filter(TaxVar!="VarName") %>%
  mutate(AbbTax = paste(Genus)) %>%
  mutate(AbbTax = gsub("NA","un",AbbTax)) %>%
  mutate(AbbTax = ifelse(AbbTax == "un", paste(Family), AbbTax)) %>%
  mutate(AbbTax = ifelse(AbbTax == "NA", paste(Order), AbbTax)) %>%
  mutate(AbbTax = ifelse(AbbTax == "NA", paste(Class), AbbTax)) %>%
  mutate(AbbTax = ifelse(AbbTax == "NA", paste(Phylum), AbbTax)) %>%
  mutate(AbbTax = ifelse(AbbTax == "NA", paste("ppmp"), AbbTax))

colnames(heatmapTab)
heatmap.2(as.matrix(heatmapTab[,-1]), scale = "column", col =
rev(bluered(100)), #lhei=lhei, lwid=lwid,lmat =lmat,
          trace = "none", density.info = "none", labRow =
heatmapTab$VarName,
          cellnote = as.matrix(heatmapTabSignT[,-1]),notecol = "black",
          labCol = LabelsCode$AbbTax)

#heatmap showing only significant taxa
matrixRes <- as.matrix(heatmapTab[,-1])
matrixSign <- as.matrix(heatmapTabSign[,-1])


####LOOK AT SEQUENCES PER TIMEPOINT####
selected.model<-output_Breeding2$inputs$y
y<-output_Breeding2$inputs$y[,1:6]
ppmp_df<-as.data.frame(output_Breeding2$inputs$y)
ppmp<-ppmp_df %>%
  dplyr::select(ppmp) %>%
  rownames_to_column(var = "VarName")

df_seqno<-as.data.frame(output_Breeding2$inputs$y[,1:6]) %>%
  rownames_to_column(var = "VarName") %>%
  filter(VarName!="B1G22") %>%
  separate(VarName, into = c("Community","Generation"),sep = "G") %>%
  mutate(G="G") %>%
  unite(G, Generation, col="Generation", sep="") %>%
  filter(Community=="R") %>%
  mutate(Generation = factor(Generation, levels = c("G1", "G2", "G3",
"G4", "G5",
                                                   "G6", "G7"))) %>%
  arrange(by_group=Generation)

B2_otu<-t(as.data.frame(output_Breeding2$inputs$y[,1:6]))
write.csv(B2_otu, "~/R/B2_ASV.csv", row.names = TRUE)
B2_metadata<-output_Breeding2$inputs$xdata
write.csv(B2_metadata, "~/R/B2_B2_metadata.csv", row.names = TRUE)
B2_taxa_df<-as.data.frame(d.clr_Breeding2) %>%
  t() %>%
  as.data.frame() %>%
  rownames_to_column(var = "Taxonomy")
B2_taxonomy<-TaxInfo %>%
  rename(Taxonomy=GenCode) %>%
  filter(Taxonomy %in% unique(B2_taxa_df$Taxonomy)) %>%
  rownames_to_column(var = "Seq") %>%
  dplyr::select(-Seq, -GenInfor) %>%
  column_to_rownames("Taxonomy")
write.csv(B2_taxonomy, "~/R/B2_taxonomy.csv", row.names = TRUE)
```

```
d.czm <- cmultRepl(y,  label=0, method="CZM")
d.clr <- t(apply(d.czm, 1, function(x){log(x) - mean(log(x))}))
d.clr_Breeding2<-d.clr

TaxCode <- TaxInfo %>%
  rename(Taxa=GenCode) %>%
  #filter(TaxVar %in% unique(filteredSign$TaxVar)) %>%
  mutate(AbbTax = paste(Genus)) %>%
  mutate(AbbTax = gsub("NA","un",AbbTax)) %>%
  mutate(AbbTax = ifelse(AbbTax == "un", paste(Family), AbbTax)) %>%
  mutate(AbbTax = ifelse(AbbTax == "NA", paste(Order), AbbTax)) %>%
  mutate(AbbTax = ifelse(AbbTax == "NA", paste(Class), AbbTax)) %>%
  mutate(AbbTax = ifelse(AbbTax == "NA", paste(Phylum), AbbTax))

B1_clr<-as.data.frame(d.clr) %>%
  rownames_to_column(var = "VarName") %>%
  filter(VarName!="B1G22") %>%
  separate(VarName, into = c("Community","Generation"),sep = "G") %>%
  mutate(G="G") %>%
  unite(G, Generation, col="Generation", sep="") %>%
  filter(Community=="B1") %>%
  mutate(Generation = factor(Generation, levels = c("G1", "G2", "G3",
"G4", "G5",
                                                 "G6", "G7"))) %>%
  arrange(by_group=Generation) %>%
  pivot_longer(cols=3:8, names_to = "Taxa", values_to = "CLR") %>%
  left_join(TaxCode, by = "Taxa") %>%
  ggplot(aes(x=Generation, y=CLR, fill=AbbTax))+
  geom_point(aes(colour=AbbTax))+
  geom_line(aes(group=AbbTax, colour=AbbTax))+
  #facet_wrap(~Cycle, scales="free_y")+
  theme(panel.grid.major = element_blank(), panel.grid.minor =
element_blank(), panel.background = element_blank(), axis.line =
element_line(colour="black"))

B2_clr<-as.data.frame(d.clr) %>%
  rownames_to_column(var = "VarName") %>%
  filter(VarName!="B1G22") %>%
  separate(VarName, into = c("Community","Generation"),sep = "G") %>%
  mutate(G="G") %>%
  unite(G, Generation, col="Generation", sep="") %>%
  filter(Community=="B2") %>%
  mutate(Generation = factor(Generation, levels = c("G1", "G2", "G3",
"G4", "G5",
                                                 "G6", "G7"))) %>%
  arrange(by_group=Generation) %>%
  pivot_longer(cols=3:8, names_to = "Taxa", values_to = "CLR") %>%
  left_join(TaxCode, by = "Taxa") %>%
  filter(AbbTax=="Pseudomonas") %>%
  ggplot(aes(x=Generation, y=CLR, fill=AbbTax))+
  geom_point(aes(colour=AbbTax))+
  geom_line(aes(group=AbbTax, colour=AbbTax))+
  #facet_wrap(~Cycle, scales="free_y")+
  theme(panel.grid.major = element_blank(), panel.grid.minor =
element_blank(), panel.background = element_blank(), axis.line =
element_line(colour="black"))
```

```
R_clr<-as.data.frame(d.clr) %>%
  rownames_to_column(var = "VarName") %>%
  filter(VarName!="B1G22") %>%
  separate(VarName, into = c("Community","Generation"),sep = "G") %>%
  mutate(G="G") %>%
  unite(G, Generation, col="Generation", sep="") %>%
  filter(Community=="R") %>%
  mutate(Generation = factor(Generation, levels = c("G1", "G2", "G3",
"G4", "G5",
                                                  "G6", "G7"))) %>%
  arrange(by_group=Generation) %>%
  pivot_longer(cols=3:8, names_to = "Taxa", values_to = "CLR") %>%
  left_join(TaxCode, by = "Taxa") %>%
  ggplot(aes(x=Generation, y=CLR, fill=AbbTax))+
  geom_point(aes(colour=AbbTax))+
  geom_line(aes(group=AbbTax, colour=AbbTax))+
  #facet_wrap(~Cycle, scales="free_y")+
  theme(panel.grid.major = element_blank(), panel.grid.minor =
element_blank(), panel.background = element_blank(), axis.line =
element_line(colour="black"))

as.data.frame(d.clr_Breeding2) %>%
  rownames_to_column(var = "VarName") %>%
  left_join(ppmp, by = "VarName") %>%
  filter(VarName!="B1G22") %>%
  separate(VarName, into = c("Community","Generation"),sep = "G") %>%
  mutate(G="G") %>%
  unite(G, Generation, col="Generation", sep="") %>%
  #filter(Community=="R") %>%
  mutate(Generation = factor(Generation, levels = c("G1", "G2", "G3",
"G4", "G5",
                                                  "G6", "G7"))) %>%
  arrange(by_group=Generation) %>%
  pivot_longer(cols=3:8, names_to = "Taxa", values_to = "CLR") %>%
  left_join(TaxCode, by = "Taxa") %>%
  #mutate(AbbTax=factor(AbbTax, levels = unique(AbbTax))) %>%
  ggplot()+
  geom_point(aes(x=Generation, y=ppmp), alpha=0.75, colour="darkgreen",
shape=23,size=2, show.legend = TRUE)+
  geom_line(aes(x=Generation, y=ppmp, group=Taxa, fill="soluble P"),
alpha=0.3, size=0.75, linetype=4, colour="darkgreen", show.legend =
TRUE)+
  geom_point(aes(x=Generation, y=CLR, colour=AbbTax), size=2.2,
alpha=0.75)+
  geom_line(aes(x=Generation, y=CLR, group=AbbTax, colour=AbbTax),
alpha=0.4, size=1)+
  geom_hline(yintercept = 0, linetype = "dashed", colour="darkgrey")+
  scale_y_continuous(sec.axis = sec_axis(trans = ~ ., name =
"solubilized P (z-score transformed)"))+
  facet_wrap(~Community, ncol=1, scales="free_y")+
  geom_hline(yintercept = -1.96, linetype = "dotted",
colour="lightgrey")+
  geom_hline(yintercept = 1.96, linetype = "dotted",
colour="lightgrey")+
  theme(panel.grid.major = element_blank(), panel.grid.minor =
element_blank(), panel.background = element_blank(), axis.line =
element_line(colour="black"))
```

```
as.data.frame(d.clr_Breeding2) %>%
  rownames_to_column(var = "VarName") %>%
  left_join(ppmp, by = "VarName") %>%
  filter(VarName!="B1G22") %>%
  separate(VarName, into = c("Community","Generation"),sep = "G") %>%
  mutate(G="G") %>%
  unite(G, Generation, col="Generation", sep="") %>%
  #filter(Community=="R") %>%
  mutate(Generation = factor(Generation, levels = c("G1", "G2", "G3",
"G4", "G5",
                                                   "G6", "G7"))) %>%
  arrange(by_group=Generation) %>%
  pivot_longer(cols=3:9, names_to = "Taxa", values_to = "CLR") %>%
  left_join(TaxCode, by = "Taxa") %>%
  mutate(AbbTax = ifelse(Taxa == "ppmp", paste("soluble P"), AbbTax))
%>%
  #mutate(AbbTax=factor(AbbTax, levels = unique(AbbTax))) %>%
  ggplot(aes(x=Generation, y=CLR, colours=AbbTax))+
  geom_point(aes(colour=AbbTax), size=2.2, alpha=0.75)+
  geom_line(aes(group=AbbTax, colour=AbbTax), alpha=0.4, size=1)+
  geom_point(aes(fill="ppmp"), alpha=0.75, colour="darkgreen",
fill="darkgreen", shape=23, show.legend = FALSE)+
  geom_line(aes(x=Generation, y=ppmp, group=Taxa, fill="ppmp"),
alpha=0.3, size=0.75, linetype=4, colour="darkgreen", show.legend =
TRUE)+
  geom_hline(yintercept = 0, linetype = "dashed", colour="darkgrey")+
  scale_y_continuous(sec.axis = sec_axis(trans = ~ ., name =
"solubilized P (z-score transformed)"))+
  facet_wrap(~Community, scales="free_y", ncol=1)+
  geom_hline(yintercept = -1.96, linetype = "dotted",
colour="lightgrey")+
  geom_hline(yintercept = 1.96, linetype = "dotted",
colour="lightgrey")+
  theme(panel.grid.major = element_blank(), panel.grid.minor =
element_blank(), panel.background = element_blank(), axis.line =
element_line(colour="black"))


as.data.frame(d.clr_Breeding2) %>%
  rownames_to_column(var = "VarName") %>%
  filter(VarName!="B1G22") %>%
  separate(VarName, into = c("Community","Generation"),sep = "G") %>%
  mutate(G="G") %>%
  unite(G, Generation, col="Generation", sep="") %>%
  #filter(Community=="R") %>%
  mutate(Generation = factor(Generation, levels = c("G1", "G2", "G3",
"G4", "G5",
                                                   "G6", "G7"))) %>%
  arrange(by_group=Generation) %>%
  pivot_longer(cols=3:9, names_to = "Taxa", values_to = "CLR") %>%
  left_join(TaxCode, by = "Taxa") %>%
  #mutate(AbbTax = ifelse(Taxa == "ppmp", paste("soluble P"), AbbTax))
%>%
  #mutate(AbbTax=factor(AbbTax, levels = unique(AbbTax))) %>%
  ggplot(aes(x=Generation, y=CLR, fill=Community))+
  geom_point(aes(colour=Community, shape=Community), size=2.2,
alpha=0.75)+
  geom_line(aes(group=Community, colour=Community), alpha=0.4, size=1)+
```

```
  facet_grid(AbbTax~., scales="free_y")+
  geom_hline(yintercept = 0, linetype = "dashed", colour="darkgrey")+
  theme(panel.grid.major = element_blank(), panel.grid.minor =
element_blank(), panel.background = element_blank(), axis.line =
element_line(colour="black"))


#######GJAM comparison of artificial selection methods#####
xdata  <- data.frame(sample_data(pdata)) %>%
  filter(Experiment != "F") %>%
  filter(Experiment != "D") %>%
  filter(Experiment != "SR") %>%
  filter(Performance!="B2") %>%
  filter(Performance!="neg_control") %>%
  filter(Performance!="pos_control") %>%
  filter(Performance!="BlankpcrCES") %>%
  #filter(Performance!="B2") %>%
  #mutate(Performance = (gsub("R", "A", Performance))) %>%
  mutate(Performance = (gsub("B1", "B", Performance))) %>%
  filter(Generation!="G0") %>%
  filter(Timepoint!="EG1C1", Timepoint!="EG1C2", Timepoint!="EG1C3",
         Timepoint!="EG1C4", Timepoint!="EG1C5", Timepoint!="EG1C6",
Timepoint!="EG1C7") %>%
  dplyr::select(-Generation) %>%
  #rename(Method=Experiment) %>%
  mutate(Experiment = (gsub("B1", "E", Experiment))) %>%
  mutate(Experiment = (gsub("B2", "P", Experiment))) %>%
  unite(Experiment, Performance, sep = "", remove=FALSE,
col="Community")

otu_B2    <- data.frame(otu_table(pdata)) %>%
  filter(Experiment == "B2")
otu_P<-otu_B2[!grepl("B2", rownames(otu_B2)),]

otu_B1<-data.frame(otu_table(pdata)) %>%
  filter(Experiment == "B1")
otu_noG0<-otu_B1[!grepl("ER", rownames(otu_B1)),]
otu_C8<-otu_noG0[grepl("C8", rownames(otu_noG0)), ]
otu_C9<-otu_noG0[grepl("C9", rownames(otu_noG0)), ]
otu_C10<-otu_noG0[grepl("C10", rownames(otu_noG0)), ]

otu<-rbind(otu_P, otu_C8, otu_C9, otu_C10)

TaxInfo<-data.frame(tax_table(pdata)) %>%
  unite("GenInfor", Kingdom:Genus, sep="_", remove=FALSE) %>%
  mutate(GenCode=paste0("Tax", 1:nrow(. )))
colnames(otu)<-TaxInfo$GenCode
View(otu)


hist( as.matrix(otu), ylab = "Reads", main = "OTU's")
#histogram looks weird HELP

min.ob<-ifelse(nrow(otu)*0.05<5,5, round(nrow(otu)*0.05,0))
nobs <- gjamTrimY( otu, minObs = min.ob, OTHER = F )$nobs   #minObs
either 5 or 5% of samples
hist( nobs/ncol(otu), nclass=100, xlab = 'Fraction of observations',
```

```
      ylab = 'Frequency', main='Incidence' ) #how common to find in
relation to all number of species



#trim by abundance instead of number of observations
tmp <- gjamTrimY(otu, minObs = min.ob)
y   <- tmp$y

TaxInfo %>%
  filter(!GenCode %in% colnames(y)) %>%
  apply(.,2,unique)

dim(otu)                     # all OTUs; 701
dim(y)                       # trimmed data; 7
tail(colnames(y))            # 'other' class added

#type names to microbial composition
d.czm <- cmultRepl(y,  label=0, method="CZM")
d.clr <- t(apply(d.czm, 1, function(x){log(x) - mean(log(x))}))
ppmp  <- as.vector(xdata$ppmp)
ydata <- cbind(y, ppmp)
head(ydata)
S     <- ncol(ydata)
typeNames    <- c(rep('CON',S-1), "CON")   # composition count data and
ppmp as continuous data

xdata$Method<-factor(xdata$Method, levels = c("E", "P"))
xdata$Performance<-factor(xdata$Performance, levels=c("A", "B"))

#run model
ml <- list( ng = 8000, burnin = 4000, typeNames = typeNames, random =
"Timepoint")
output <- gjam(~ Method*Performance, xdata, ydata, modelList = ml)

fit <- gjamPlot(output, plotPars = list(GRIDPLOTS=T, SAVEPLOTS=TRUE) )


output_CompBreeding<-output
fit_CompBreeding<-fit

###diagnosis of MCMC chains to see if model is okay to continue

#Add here your settings for Gibbs sampling
ng <- 8000
burning <- 4000

#Add here you output from the GJAM analysis
selected.model <- output_CompBreeding

plot(cumsum(selected.model$chains$bgibbs[,1]-
mean(selected.model$chains$bgibbs[,1])),
     type="l", xlab="iteration", ylab="y")
#plot(selected.model$chains$bgibbsUn[,1]-
mean(selected.model$chains$bgibbsUn[,1]),
#    type="l", xlab="iteration", ylab="y")

plot(selected.model$chains$bgibbs[,1])
```

```
# center with 'apply()'
center_apply <- function(x) {
  apply(x, 2, function(y) y - mean(y))
}

# apply it
dfplot <-
data.frame(center_apply(data.frame(selected.model$chains$bgibbs)))
#dfplot <-
data.frame(center_apply(data.frame(selected.model$chains$bgibbs[burning
:ng,])))
#dfplot <-
data.frame(center_apply(data.frame(selected.model$chains$sgibbs)))

plot(dfplot[,5],type="l", xlab="iteration", ylab="y")
dim(dfplot)

#bgibbs
DiagTab <- dfplot %>%
  #  data.frame() %>%
  mutate(Iteration = 1:nrow(dfplot)) %>%
  gather(key = "Parameter", value = "value", -Iteration) %>%
  # filter(Iteration>burning) %>%
  group_by(Parameter) %>%
  summarise(Median = mean(value, na.rm = TRUE), CIlow = quantile(value,
0.025, na.rm = TRUE),
            CIhigh = quantile(value, 0.975, na.rm = TRUE),IQR =
IQR(value)) %>%
  ungroup() %>%
  filter(IQR>quantile(IQR,0.975)) %>% #Q3
  # filter(IQR>10) %>%
  separate(Parameter, into = c("Taxa","Treatment"),sep = "_", remove =
FALSE)

#sgibbs
#DiagTab <- dfplot %>%
#  data.frame() %>%
# mutate(Iteration = 1:nrow(dfplot)) %>%
# gather(key = "Parameter", value = "value", -Iteration) %>%
# filter(Iteration>burning) %>%
# group_by(Parameter) %>%
# summarise(Median = mean(value, na.rm = TRUE), CIlow = quantile(value,
0.025, na.rm = TRUE),
#           CIhigh = quantile(value, 0.975, na.rm = TRUE),IQR =
IQR(value)) %>%
#ungroup() %>%
#filter(IQR>quantile(IQR,0.975))

dim(DiagTab)
View(DiagTab)    #DiagTab gives the samples with the highest divergent
summary(DiagTab)

summary(DiagTab$IQR)

hist(DiagTab$IQR)
boxplot(DiagTab$IQR)     #important to see in which range the variation
happens; if small go on
```

```
length(unique(DiagTab$Parameter))
Notconverged <- unique(DiagTab$Parameter)
unique(DiagTab$Taxa)

plot(dfplot[,Notconverged[1]]-mean(dfplot[,Notconverged[1]]),type="l",
xlab="iteration", ylab="y")
plot(cumsum(dfplot[,Notconverged[1]]),type="l", xlab="iteration",
ylab="y")

View(selected.model$inputs$y[,unique(DiagTab$Taxa)])
heatmap(scale(selected.model$inputs$y[,unique(DiagTab$Taxa)]))
apply(selected.model$inputs$y[,unique(DiagTab$Taxa)],2,hist)

library(ggplot2)
#Looking to the chains that did not converged
dfplot %>%
  # mutate_all(list(cumsum)) %>%
  #  data.frame() %>%
  mutate(ng = 1:nrow(dfplot)) %>%
  # filter(ng>burning) %>%
  tidyr::gather(key = VarName, value = MCMC, -ng) %>%
  # filter(VarName %in% sample(Notconverged,20)) %>%
  filter(VarName %in% Notconverged) %>% #If smaller than 20
  ggplot(aes(x = ng, y = MCMC, color = VarName))+
  geom_line()+
  # scale_y_log10()+
  theme(panel.grid.major = element_blank(), panel.grid.minor =
element_blank(), panel.background = element_blank(), axis.line =
element_line(colour="black"))


#Randomly checking the converged chains
SampleRandom <- sample(colnames(dfplot),20)
dfplot %>%
  #  data.frame() %>%
  mutate(ng = 1:nrow(dfplot)) %>%
  filter(ng>burning) %>%
  tidyr::gather(key = VarName, value = MCMC, -ng) %>%
  #filter(VarName %in% SampleRandom) %>%
  ggplot(aes(x = ng, y = MCMC, color = VarName))+
  geom_line()+
  #theme(panel.grid.major = element_blank(), panel.grid.minor =
element_blank(), panel.background = element_blank(), axis.line =
element_line(colour="black"))
  #theme(legend.position = "none")
  theme(panel.grid.major = element_blank(), panel.grid.minor =
element_blank(), panel.background = element_blank(), axis.line =
element_line(colour="black"))


dfplot %>%
  #  data.frame() %>%
  mutate(ng = 1:nrow(dfplot)) %>%
  filter(ng>burning) %>%
  tidyr::gather(key = VarName, value = MCMC, -ng) %>%
  filter(VarName %in% SampleRandom) %>%
  ggplot(aes(x = VarName, y = MCMC, color = VarName))+
  geom_boxplot()+
```

```
  theme(legend.position = "none")

#if stable save data for next step
save(output_Breeding2_PP,file="gjamOutput_Breeding2_PP.RData")

#comparison of reads with CLR
y<-ydata[,1:6]
d.czm <- cmultRepl(y,  label=0, method="CZM")
d.clr <- t(apply(d.czm, 1, function(x){log(x) - mean(log(x))}))
d.clr_CompBreeding<-d.clr

TaxCode <- TaxInfo %>%
  rename(Taxa=GenCode) %>%
  #filter(TaxVar %in% unique(filteredSign$TaxVar)) %>%
  mutate(AbbTax = paste(Genus)) %>%
  mutate(AbbTax = gsub("NA","un",AbbTax)) %>%
  mutate(AbbTax = ifelse(AbbTax == "un", paste(Family), AbbTax)) %>%
  mutate(AbbTax = ifelse(AbbTax == "NA", paste(Order), AbbTax)) %>%
  mutate(AbbTax = ifelse(AbbTax == "NA", paste(Class), AbbTax)) %>%
  mutate(AbbTax = ifelse(AbbTax == "NA", paste(Phylum), AbbTax))

heatmap_xdata<-as.data.frame(xdata) %>%
  rownames_to_column(var="VarName") %>%
  dplyr::select(-SampleID)

heatmap_clr<-as.data.frame(d.clr) %>%
  rownames_to_column(var = "VarName") %>%
  left_join(heatmap_xdata, by = "VarName") %>%
  unite(Method, Performance, col="Community") %>%
  arrange(by_group=Community) %>%
  pivot_longer(cols=2:7, names_to = "Taxa", values_to = "CLR") %>%
  group_by(Community, Taxa) %>%
  summarise(CLR, avg=mean(CLR)) %>%
  distinct(avg) %>%
  pivot_wider(names_from = Taxa, values_from = avg) %>%
  filter(Community!="P_B2") %>%
  column_to_rownames("Community") %>%
  as.matrix()


hT <- heatmap.2(heatmap_clr, scale = "none", col = rev(bluered(100)),
cexRow =1, cexCol=1, #lhei=lhei, lwid=lwid,lmat =lmat,
              trace = "none", density.info = "none", labCol =
LabelsCode$AbbTax)

LabelsCode <- data.frame(GenCode = colnames(heatmap_clr)) %>%
  left_join(TaxInfo,by = c("GenCode")) %>%
  mutate(AbbTax = paste(Genus)) %>%
  mutate(AbbTax = gsub("NA","un",AbbTax)) %>%
  mutate(AbbTax = ifelse(AbbTax == "un", paste(Family), AbbTax)) %>%
  mutate(AbbTax = ifelse(AbbTax == "NA", paste(Order), AbbTax)) %>%
  mutate(AbbTax = ifelse(AbbTax == "NA", paste(Class), AbbTax)) %>%
  mutate(AbbTax = ifelse(AbbTax == "NA", paste(Phylum), AbbTax)) %>%
  mutate(AbbTax=ifelse(GenCode=="ppmp", paste("soluble P"), AbbTax))
```
