# Supplementary material for: Enhancing phosphate-solubilising microbial communities through artificial selection
Source: Nat Commun. 2024 Feb 23;15:1649. doi: 10.1038/s41467-024-46060-x (PMC10884399; doi:10.1038/s41467-024-46060-x)
Supplement: Supplementary file 3 — Reporting Summary [file 41467_2024_46060_MOESM3_ESM.pdf]

nature portfolio

Corresponding author(s): Eiko E. Kuramae

Last updated by author(s): Feb 11, 2024

# Reporting Summary

Nature Portfolio wishes to improve the reproducibility of the work that we publish. This form provides structure for consistency and transparency in reporting. For further information on Nature Portfolio policies, see our Editorial Policies and the Editorial Policy Checklist.

## Statistics

For all statistical analyses, confirm that the following items are present in the figure legend, table legend, main text, or Methods section.

| n/a | Confirmed | |
|---|---|---|
| ☐ | ☒ | The exact sample size (*n*) for each experimental group/condition, given as a discrete number and unit of measurement |
| ☐ | ☒ | A statement on whether measurements were taken from distinct samples or whether the same sample was measured repeatedly |
| ☐ | ☒ | The statistical test(s) used AND whether they are one- or two-sided<br>*Only common tests should be described solely by name; describe more complex techniques in the Methods section.* |
| ☐ | ☒ | A description of all covariates tested |
| ☐ | ☒ | A description of any assumptions or corrections, such as tests of normality and adjustment for multiple comparisons |
| ☐ | ☒ | A full description of the statistical parameters including central tendency (e.g. means) or other basic estimates (e.g. regression coefficient) AND variation (e.g. standard deviation) or associated estimates of uncertainty (e.g. confidence intervals) |
| ☐ | ☒ | For null hypothesis testing, the test statistic (e.g. *F*, *t*, *r*) with confidence intervals, effect sizes, degrees of freedom and *P* value noted<br>*Give P values as exact values whenever suitable.* |
| ☐ | ☒ | For Bayesian analysis, information on the choice of priors and Markov chain Monte Carlo settings |
| ☒ | ☐ | For hierarchical and complex designs, identification of the appropriate level for tests and full reporting of outcomes |
| ☒ | ☐ | Estimates of effect sizes (e.g. Cohen's *d*, Pearson's *r*), indicating how they were calculated |

*Our web collection on statistics for biologists contains articles on many of the points above.*

## Software and code

Policy information about availability of computer code

| | |
|---|---|
| Data collection | No software was used |
| Data analysis | R v4.2.0, DADA2 version 1.16, Generalized joint attribute modeling (GJAM, gjam package v2.6.2), DivNet, generalised linear models (GLM); linear mixed-effect models (LMM; lme4 package v1.1.34); estimated marginal means (EMMs, emmeans package v1.8.9), ExpDes R package (v1.2.2), multcomp R package (v1.4.25), gplots package (v3.1.3), qgraph package (v1.9.5) |

For manuscripts utilizing custom algorithms or software that are central to the research but not yet described in published literature, software must be made available to editors and reviewers. We strongly encourage code deposition in a community repository (e.g. GitHub). See the Nature Portfolio guidelines for submitting code & software for further information.

## Data

Policy information about availability of data

All manuscripts must include a data availability statement. This statement should provide the following information, where applicable:

- Accession codes, unique identifiers, or web links for publicly available datasets
- A description of any restrictions on data availability
- For clinical datasets or third party data, please ensure that the statement adheres to our policy

Raw sequencing data was deposited in ENA with accession number PRJEB64328 (ENA-FIRST-PUBLIC: 2024-01-09). The taxonomy was assigned to the amplicon sequencing variants (ASVs) with the SILVA database (v138) (https://www.arb-silva.de/no_cache/download/archive/release_138/). The source data generated in this study are provided as a Source Data file with this paper.

## Research involving human participants, their data, or biological material

Policy information about studies with human participants or human data. See also policy information about sex, gender (identity/presentation), and sexual orientation and race, ethnicity and racism.

| | |
|---|---|
| Reporting on sex and gender | N/A |
| Reporting on race, ethnicity, or other socially relevant groupings | N/A |
| Population characteristics | N/A |
| Recruitment | N/A |
| Ethics oversight | N/A |

Note that full information on the approval of the study protocol must also be provided in the manuscript.

# Field-specific reporting

Please select the one below that is the best fit for your research. If you are not sure, read the appropriate sections before making your selection.

☐ Life sciences ☐ Behavioural & social sciences ☒ Ecological, evolutionary & environmental sciences

For a reference copy of the document with all sections, see nature.com/documents/nr-reporting-summary-flat.pdf

# Ecological, evolutionary & environmental sciences study design

All studies must disclose on these points even when the disclosure is negative.

| | |
|---|---|
| Study description | Artificial selection of microbial communities with factorial design. Factors: Passage, Cycle, Fraction, Dilution, Performance, Selection Method. 3 replicates per microbial community in the first generation, 8 to 12 replicates within the selection lines in the offspring generation.<br>Plant experiment: randomized complete block design. Factors: Treatment. 3 replicates per treatment. 2 sterile control treatments (1 with soluble P, one with insoluble P) |
| Research sample | Soil microorganisms from Dutch natural grassland soil, separated by fractioning and diluting. The soil was chosen because it was part of another project that already detected some potential plant growth promoting traits. |
| Sampling strategy | At least 3 replicates were chosen to perform statistical analysis. In the plant experiment, every treatment was replicated in six biologically independent replicates each containing three Chrysanthemum cuttings. The number of replicates in the selection lines were chosen based on published recommendations (see Mueller and Sachs 2015, Trends in Microbiology). |
| Data collection | The first author of the manuscript collected the data through measurements (e.g., spectrophotometer, weight). |
| Timing and spatial scale | April 2021 to March 2022. Phosphate solubilization activity of the communities were measured after every 3 days (incubations: Monday to Thursday, Friday to Monday). In the plant experiment, the nutrient solution was sampled after 4 days (similar to in vitro incubation time), 7 days and 21 days. |
| Data exclusions | No data exclusion |
| Reproducibility | We showed reproducibility of the stabilization in multiple phases of the artificial selection experiment (e.g., stabilization of the parental phase and environmental perturbation phase). Phosphate solubilisation activity was measured in at least 3 replicates per seeding community. The hydroponics experiment had six biologically independent replicates per treatment. |
| Randomization | Plant experiment: randomized complete block design by treatments |

| | |
|---|---|
| | Artificial selection: communities were allocated to the active selection line based on performance (best-performing community seeds the offspring community). As a control, a random selection was conducted where the communities were selected and allocated to the selection line independent to their community function. Furthermore, the dilution due to serial passaging can be considered as somewhat random sampling caused by the large number of community members in microbial communities. To ensure that there was no effect of placement in the incubator, we also randomized the order of samples while incubating. |
| Blinding | The investigators did not implement blinding since our experimental setup involved objective and quantifiable measurements (e.g., soluble P concentration, plant biomass, etc.) minimizing the potential for subjective biases. |

Did the study involve field work? ☐ Yes ☒ No

# Reporting for specific materials, systems and methods

We require information from authors about some types of materials, experimental systems and methods used in many studies. Here, indicate whether each material, system or method listed is relevant to your study. If you are not sure if a list item applies to your research, read the appropriate section before selecting a response.

## Materials & experimental systems

| n/a | Involved in the study | |
|---|---|---|
| ☒ | ☐ | Antibodies |
| ☒ | ☐ | Eukaryotic cell lines |
| ☒ | ☐ | Palaeontology and archaeology |
| ☒ | ☐ | Animals and other organisms |
| ☒ | ☐ | Clinical data |
| ☒ | ☐ | Dual use research of concern |
| ☐ | ☒ | Plants |

## Methods

| n/a | Involved in the study | |
|---|---|---|
| ☒ | ☐ | ChIP-seq |
| ☒ | ☐ | Flow cytometry |
| ☒ | ☐ | MRI-based neuroimaging |

# Dual use research of concern

Policy information about dual use research of concern

## Hazards

Could the accidental, deliberate or reckless misuse of agents or technologies generated in the work, or the application of information presented in the manuscript, pose a threat to:

| No | Yes | |
|---|---|---|
| ☒ | ☐ | Public health |
| ☒ | ☐ | National security |
| ☒ | ☐ | Crops and/or livestock |
| ☒ | ☐ | Ecosystems |
| ☒ | ☐ | Any other significant area |

## Experiments of concern

Does the work involve any of these experiments of concern:

| No | Yes | |
|---|---|---|
| ☒ | ☐ | Demonstrate how to render a vaccine ineffective |
| ☒ | ☐ | Confer resistance to therapeutically useful antibiotics or antiviral agents |
| ☒ | ☐ | Enhance the virulence of a pathogen or render a nonpathogen virulent |
| ☒ | ☐ | Increase transmissibility of a pathogen |
| ☒ | ☐ | Alter the host range of a pathogen |
| ☒ | ☐ | Enable evasion of diagnostic/detection modalities |
| ☒ | ☐ | Enable the weaponization of a biological agent or toxin |
| ☒ | ☐ | Any other potentially harmful combination of experiments and agents |
